# Metallohelices that kill Gram-negative pathogens using intracellular antimicrobial peptide pathways†
†Electronic supplementary information (ESI) available: Data created during this study are openly available from the University of Warwick Research Archive Portal (WRAP) (http://wrap.warwick.ac.uk). CCDC 1904782–1904784. For ESI and crystallographic data in CIF or other electronic format see DOI: 10.1039/c9sc03532j


**DOI:** 10.1039/c9sc03532j

**Published:** 2019-09-05

**Authors:** Daniel H. Simpson, Alexia Hapeshi, Nicola J. Rogers, Viktor Brabec, Guy J. Clarkson, David J. Fox, Ondrej Hrabina, Gemma L. Kay, Andrew K. King, Jaroslav Malina, Andrew D. Millard, John Moat, David I. Roper, Hualong Song, Nicholas R. Waterfield, Peter Scott

**Affiliations:** a Department of Chemistry , University of Warwick , Gibbet Hill Road , Coventry , CV4 7AL , UK . Email: peter.scott@warwick.ac.uk; b Warwick Medical School , University of Warwick , Coventry , CV4 7AL , UK; c The Czech Academy of Sciences , Institute of Biophysics , Kralovopolska 135 , CZ-61265 Brno , Czech Republic; d School of Life Sciences , University of Warwick , Gibbet Hill Campus , Coventry , CV4 7AL , UK; e Department of Biophysics , Palacky University , Slechtitelu 27 , 783 71 Olomouc , Czech Republic

## Abstract

A range of new water-compatible optically pure metallohelices – made by self-assembly of simple non-peptidic organic components around Fe ions – exhibit similar architecture to some natural cationic antimicrobial peptides (CAMPs) and are found to have high, structure-dependent activity against bacteria, including clinically problematic Gram-negative pathogens. A key compound is shown to freely enter rapidly dividing *E. coli* cells without significant membrane disruption, and localise in distinct foci near the poles. Several related observations of CAMP-like mechanisms are made *via* biophysical measurements, whole genome sequencing of tolerance mutants and transcriptomic analysis. These include: high selectivity for binding of G-quadruplex DNA over double stranded DNA; inhibition of both DNA gyrase and topoisomerase I *in vitro*; curing of a plasmid that contributes to the very high virulence of the *E. coli* strain used; activation of various two-component sensor/regulator and acid response pathways; and subsequent attempts by the cell to lower the net negative charge of the surface. This impact of the compound on multiple structures and pathways corresponds with our inability to isolate fully resistant mutant strains, and supports the idea that CAMP-inspired chemical scaffolds are a realistic approach for antimicrobial drug discovery, without the practical barriers to development that are associated with natural CAMPS.

## Abstract 2

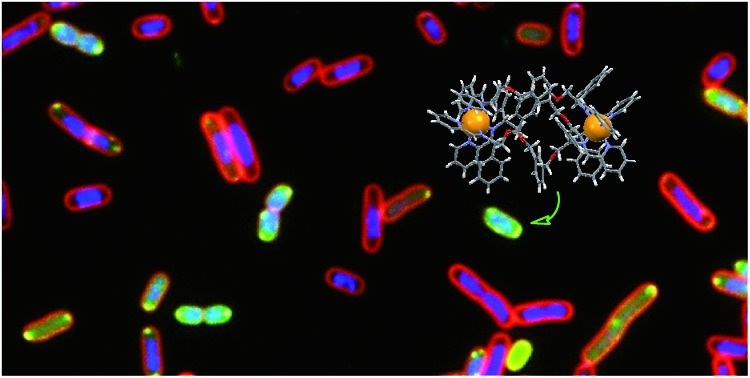
Iron-based self-assembled optically pure compounds mimic the mechanisms of small peptides, according to biophysical, genomic, transcriptomic and other analyses.

## Introduction

Bacteria naturally evolve resistance to most antimicrobial compounds, and the widespread careless use of antibiotics is exacerbating the situation. When coupled with a dearth of discovery of new antimicrobial drugs, antimicrobial resistance has emerged as a grave threat to current and future medical practice and socioeconomic activity, as recently highlighted by numerous organizations and governments.^1^,^2^

Antimicrobial peptides are part of the innate immune systems of plants and animals that evolved to defend against a range of microbes.^3^,^4^ They are typically small molecules, comprising 10–50 amino acids. The majority – the so-called cationic antimicrobial peptides (CAMPs) – have an excess of both cationic and hydrophobic subunits arranged into secondary structures, such that the folded assembly exhibits an amphipathic or patchy charge distribution. While the surfaces of eukaryotic cell membranes are typically neutral, those of bacteria are intrinsically anionic due to the incorporation of negatively charged phospholipids, teichoic acid or lipopolysaccharides (LPS).^5^,^6^ Therefore, CAMPs have strong electrostatic interactions with bacterial cell membranes but not those of the host cells, providing generalised specificity. In some cases, the site of action is the membrane itself, with the CAMP inducing fatal changes in membrane structure and fluidity, or the creation of ungated pores allowing uncontrolled solute exchange with the environment.^7^,^8^ Alternatively, CAMPs may cross the membrane barrier and interact with intracellular targets with lethal consequences^9^–^11^ or indeed have combinations of modes of action.^4^,^12^ CAMPs and derivatives have been investigated as clinical antibiotics,^13^–^15^ but unfortunately their pharmacokinetic profiles tend to be unfavourable and large scale manufacture presents a challenge.^16^ Nevertheless it is worth noting that a number of CAMPs are currently under commercial development, with attempts being made to enhance the properties of natural systems using strategies such as peptide stapling^17^ to improve structural integrity and enzymatic stability.

**Scheme 1:**
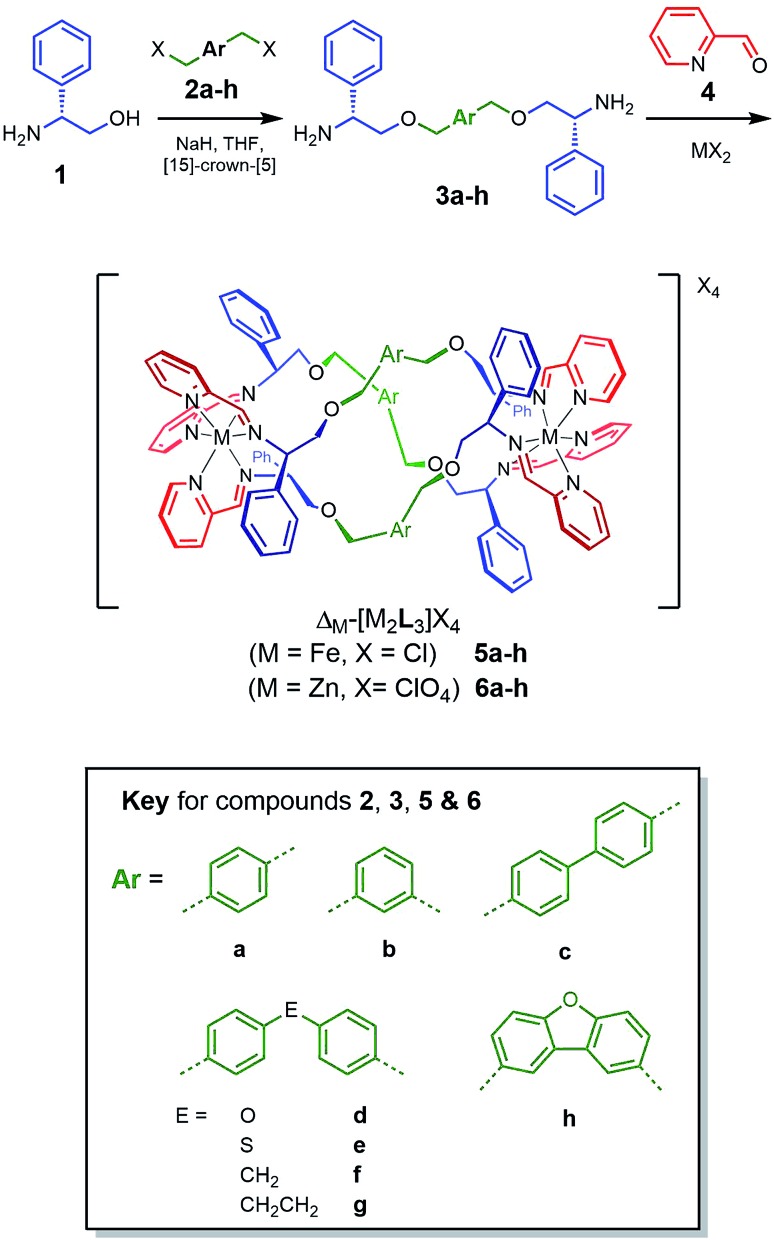
Synthesis and characterisation of new metallohelices. Self-assembly of series 5 and 6 incorporating a range of linker units *via* optically pure diamines 3.

Our recently-developed ranges of cationic metallohelices, based on helical arrays of fully-encapsulated Fe ions (*e.g.*Scheme 1), have similar diameter to CAMPs, are optically pure, water-soluble and are available *via* efficient self-assembly on a multi-gram scale. Recently we have shown that one class^18^,^19^ has a facially amphipathic architecture akin to that of natural antifreeze peptides^20^ and a similar structure binds to the central hydrophobic α-helical region of an amyloid β protein and attenuates toxicity.^21^ Members of another class^22^ have excellent and highly selective activity against a variety of cancer cell lines. In stark contrast, none of the above ranges have significant antimicrobial activity. Intriguingly however, a lone prototype compound of another class^23^**5a** (Scheme 1), was shown to have good activity against a limited panel of microbes. A new synthetic protocol has opened up this area of chemical space and we report here compounds with structure-dependent activity against Gram-positive and -negative bacteria. One potent compound is shown to freely enter *E. coli* cells and to exhibit antimicrobial peptide-like mechanisms of action.

## Results and discussion

We reported that optically pure phenylglycinol **1** (Scheme 1) reacts in the presence of NaH with benzylic bromide **2a** to give diamine **3a**.^24^ However, all other examples of such etherifications we have attempted led to inseparable mixtures containing the *N*-alkylated isomers. After extensive experimentation with solvents, bases and *N*-protection strategies we found that performing the reaction in the presence of [15]-crown-[5]^25^ gave clean products. The optical purity of the subsequent range of new diamines **3b–h** was established *via* synthesis of the (*R*)-(+)-Mosher's acid derivatives (ESI†). This achieved, the self-assembly of the various metallohelices incorporating 2-pyridinecarboxadlehyde **4** and M(ii) salts proceeded smoothly to give a range of structures.

With iron(ii) chloride as the metal ion source, both enantiomers of each of the water-soluble systems **5a–h** were produced while zinc(ii) perchlorate gave the isostructural analogues **6a–h**. All compounds were characterised by multinuclear NMR techniques, mass spectrometry, microanalysis, and where possible single crystal X-ray diffraction. Circular dichroism (CD) spectroscopy, thermo-gravimetric analysis (TGA), IR and UV-visible spectroscopies were additionally applied to the Fe compounds.

**Fig. 1:**
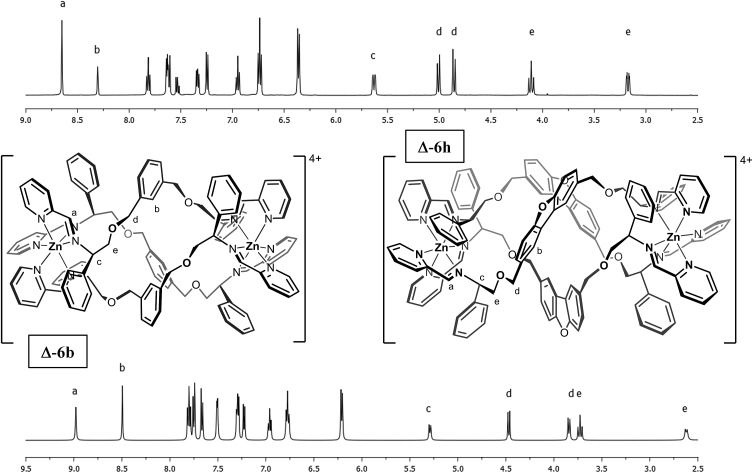
^1^H-NMR spectra of metallohelices **Δ-6b** and **Δ-6h** (500 MHz in *d*^3^-acetonitrile at 298 K). Note the presence of single diastereomers and the unusual chemical shifts of the nuclei at position b in both cations.

The complexes contained a single set of ligand peaks in the ^1^H and ^13^C NMR spectra (ESI) consistent with the presence of single diastereomers (Fig. 1), and since the ligands are optically pure, the complexes are single enantiomers. As we have described in detail,^18^,^23^,^25^,^26^ the mechanisms for the extremely reliable diastereoselection using our α-methylbenzyliminopydridine unit used in these compounds also contribute substantially to the unusual stability to hydrolysis (*vide infra*).

**Fig. 2:**
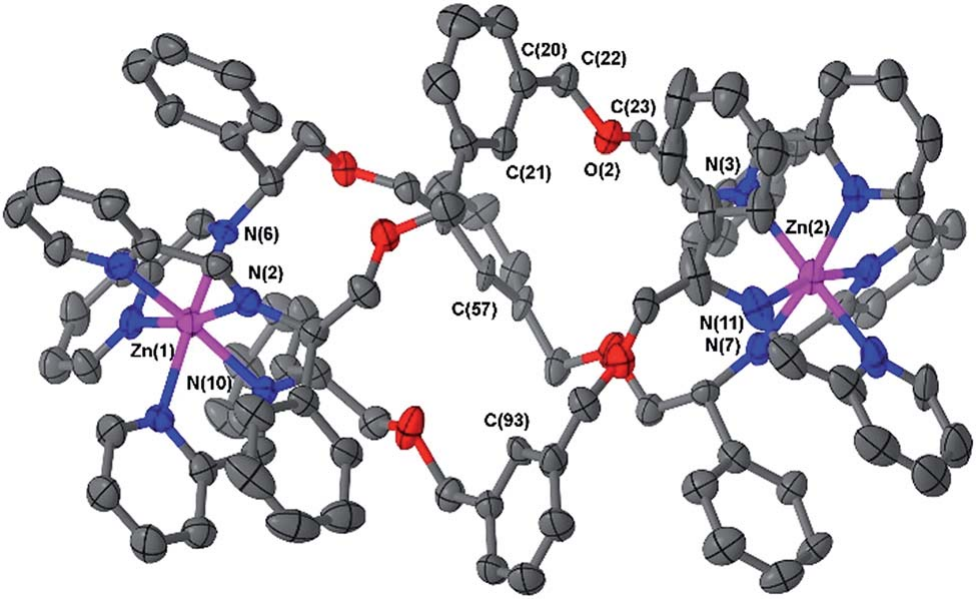
Structure of the cationic unit of **Δ_Zn_-6b**. Thermal ellipsoid plot (50% probability) with H atoms, solvent molecules and counterions removed for clarity.

Slow vapour diffusion of ethyl acetate into a concentrated acetonitrile solution of **6b** afforded single crystals suitable for XRD. The cationic unit of the compound (Fig. 2) shows the approximate octahedral coordination environments at the Zn centres, and the now familiar inter-ligand π–π stacking interactions, responsible in large part for the excellent diastereoselection.^18^,^23^,^26^

The overall size and shape of the **6b** cation is more markedly folded or “concertinaed” than **6a**.^26^ The inter-metallic distance is substantially shortened (12.4 Å *vs.* 14.0 Å) while the circumference of the molecule is increased, as evidenced by the average distance between the centroids of each xylenyl aromatic ring: 7.6 Å, *vs.* 6.3 Å for **6a**. Correspondingly the *m*-xylenyl bridge also folds with a more pronounced helical turn along the metal–metal axis: torsion angles between Zn–N(imine) bonds from the same ligand, along the Zn–Zn axis [*e.g.* N(6)–Zn(1)–Zn(2)–N(7)] range from 100.9° to 101.0°. It should be noted however that as with **6a** the *P*-helicity generated by each **Λ-Zn** centre is inverted at the C-stereogenic centre such that the bridge units fold with *M*-helicity. In **6b** all the *m*-xylenyl groups are oriented such that the C–H groups at C(21), C(57), and C(93) point towards the centre of the complex. Since the plane of each xylenyl group intersects this cavity, the centre of the complex is expected to be a deshielding environment as a result of the coinciding ring currents. This explains the unusually downfield-shifted ^1^H-NMR signal for these nuclei (8.30 ppm). The system **6h** (Fig. 1) has a similar feature.

**Fig. 3:**
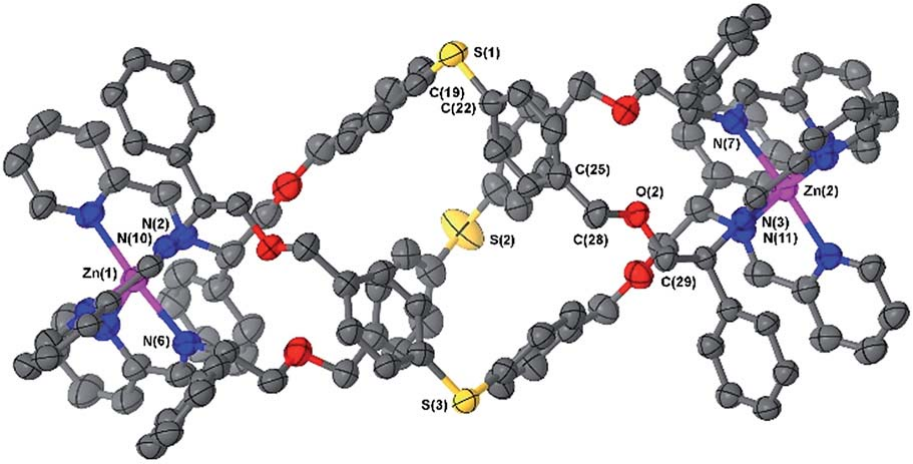
Structure of the cationic unit of **6e**. Thermal ellipsoid plot (50% probability) with H atoms, solvent molecules and counterions removed for clarity.

Naturally, the longer bridge of thioether **6e** results in a longer complex than **6a** or **b** with an inter-metal distance of 17.4 Å (Fig. 3). Interestingly, the ‘hinge’ created by the sulfur atoms appears to aid the folding process by allowing the bridge to arch outwards, with the two flanking *p*-tolyl units able to orient separately, and furthermore allowing the bridge a remarkable twist along the metal–metal axis.

The torsion angles between Zn–N(imine) bonds from the same ligand range from 167.3° to 179.1°. As a consequence of this arching of the bridge the sulfur atoms protrude from the helix and the system appears to have a larger circumference than earlier examples (S–S distances range 8.0 Å to 9.2 Å).

The structure does not appear to be distorted significantly from threefold symmetry; the C–S–C bond angles [*e.g.* C(19)–S(1)–C(22)] fall within a narrow and conventional range (103–104°). The C^Ph^–CH_2_–O–CH_2_ torsion angles [*e.g.* C(25)–C(28)–O(2)–C(29)] range from 162° to 171°; slightly lower than the essentially antiperiplanar arrangement in *meta*-bridged **6b**. An angle between imine N-atom planes [N(2)–N(6)–N(10) and N(3)–N(7)–N(11)] of 9.9° (comparable to **6a** and **6b**) is also supportive of there being little torsional strain in this complex.

**Fig. 4:**
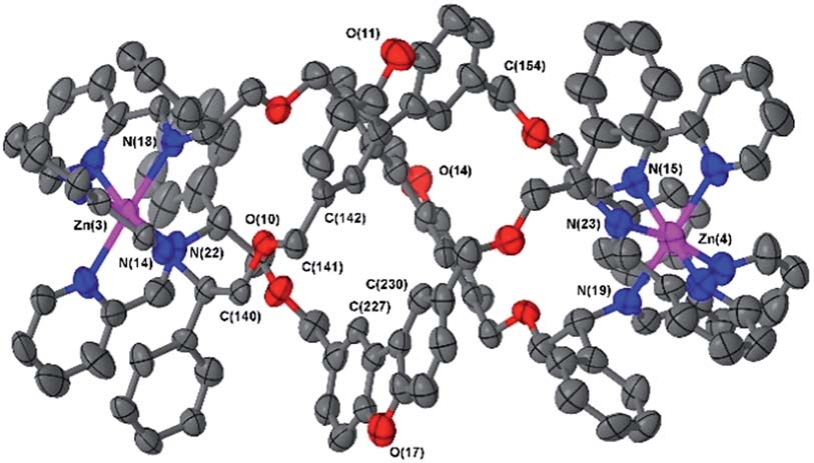
Structure of the cationic unit of 6 h. Thermal ellipsoid plot (50% probability) with H atoms, solvent molecules and counterions removed for clarity.

The Δ_Zn_ cationic unit of dibenzofuran-bridged **6h** is shown in Fig. 4. The intermetallic distance of 14.4 Å is rather smaller than that observed in the related structure **6e** corresponding to a more compact concertina fold. As expected, the circumference is slightly larger, with distances between ‘apex’ O atoms [O(11), O(14) and O(17)] falling into a narrow range (9.9–10.1 Å). These three atoms form an approximate equilateral triangle, indicating high symmetry of the system. There is also a lack of distortion arising from ring strain: each C^Ph^–CH_2_–O–CH_2_ torsion angle [*e.g.* C(142)–C(141)–O(10)–C(140)] falls into the range 173–180° (effectively antiperiplanar). As a consequence of this efficient helication the CH groups at C(227) and C(230) are directed inwards, similar to the observation for **6b**, leading to a strong downfield shift in the ^1^H-NMR spectrum (9.0 ppm, Fig. 1). In addition, the angle between imine N atom planes [N(2)–N(6)–N(10) and N(3)–N(7)–N(11)] is exceptionally shallow at 3.3°. Torsion angles between Zn–N(imine) bonds from the same ligand, along the Zn–Zn axis range 159.8° to 162.5°, greater than **6b** and less than in **6e**; this is in line with **6h** having the intermediate inter-metal distance of the three.

In summary, while the helical configurations of all these metallohelices are controlled by π-stacking and steric effects^23^,^27^ so as to be exclusively right or left handed (Δ or Λ) depending on choice of enantiomer **1**, the various diamines **3** cause different folding behaviours in the assemblies **5** and **6**. Overall the range of architectures is such that intermetallic distances – the relative positions of the seats of encapsulated positive charge – vary from 12.4 Å (**5**/**6b**) to over 17.4 Å (**5**/**6e**), and the van der Waals diameters of the structures are in a range from *ca.* 11.5 Å (**5**/**6a**) to 15.0 Å (**5**/**6h**).

**Fig. 5:**
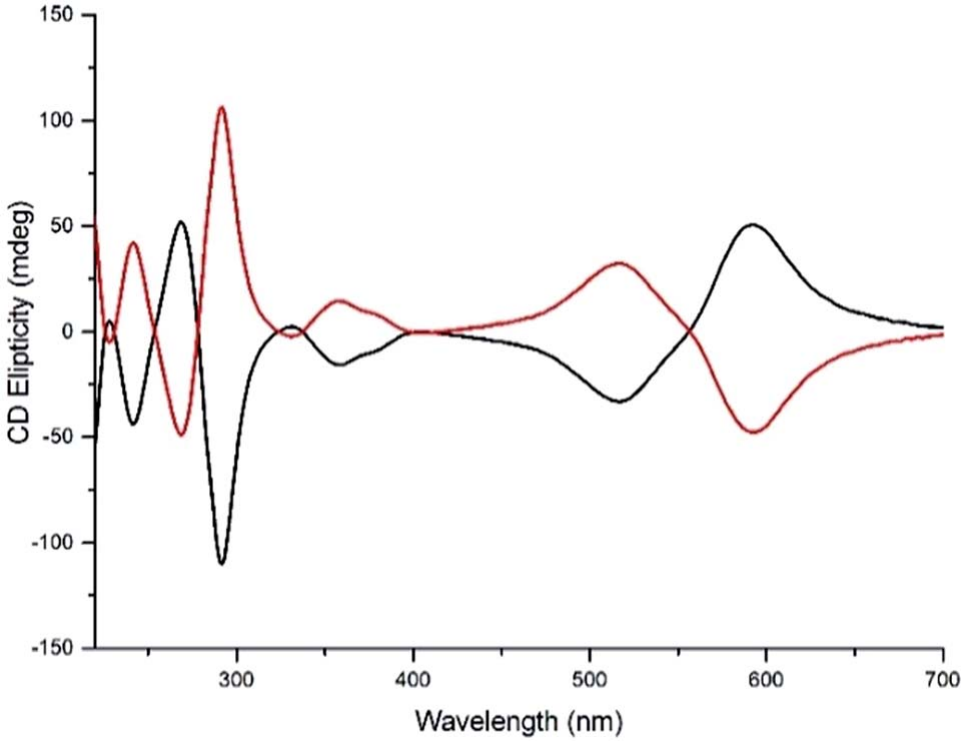
Circular dichroism spectra of enantiomers **Δ-5b** (black) and **Λ-5b** (0.03 mM in water).

Water compatible systems suitable for antimicrobial screening were prepared by similar self-assembly reactions (Scheme 1) using iron(ii) chloride in methanol. ^1^H and ^13^C NMR spectra were very similar to the Zn counterparts, suggesting strong structural similarity. Typically, ^1^H-NMR spectra were slightly broader, and all contained a large peak at *ca.* 4.9 ppm due to the presence of water of crystallization. Quantification of the latter was achieved through a combination of microanalysis and thermogravimetric analysis (TGA) – see ESI.† The Fe compounds **5** gave excellent electrospray-ionisation mass spectra (ESI-MS), whereby the [Fe_2_L_3_]^4+^ ion was readily observed at high resolution. Circular dichroism (CD) spectra of pairs of enantiomers **5** were mirror images within error (Fig. 5 and ESI†).

The diverse but efficient folding behaviours described above contribute to exceptional stability to hydrolysis in this class of metallohelix and *e.g.***5b** has a *t*_1/2_ of >10 d even at pH 1.5 (Fig. S8†).

### *In vitro* antibacterial activity

**Fig. 6:**
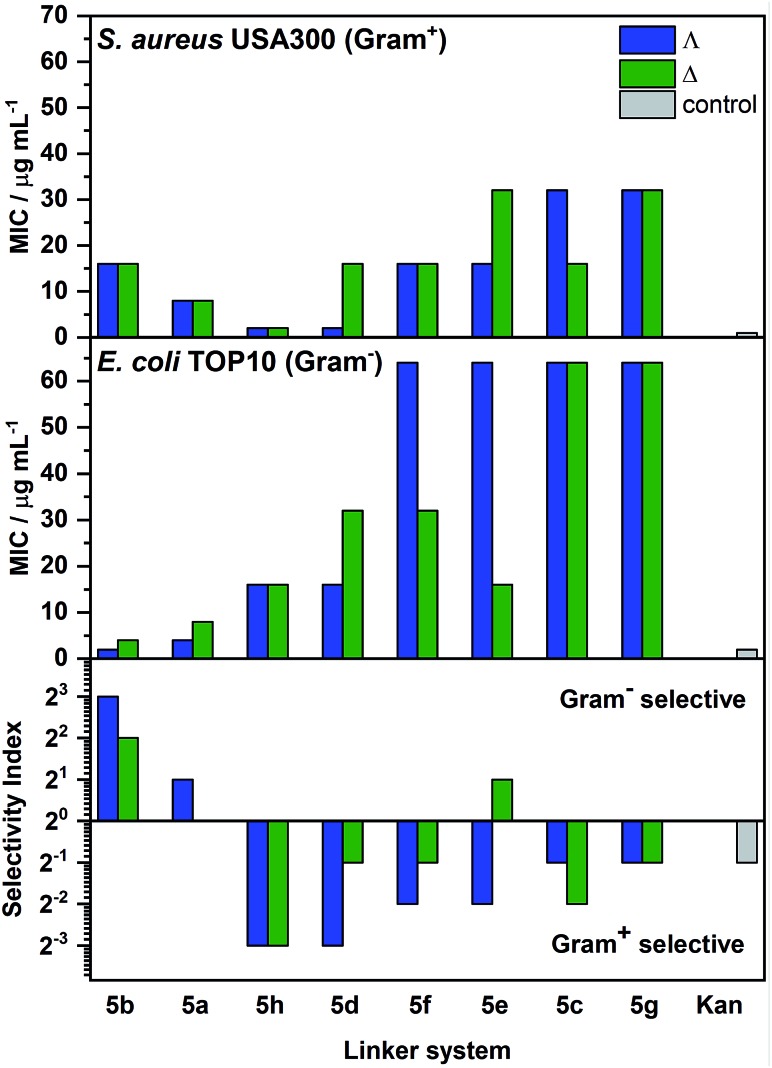
Activity of the metallohelices against *S. aureus* and *E. coli*, and corresponding selectivity. Pairs of enantiomers (blue and green bars) are ordered along the *x*-axis by intermetallic distance (increasing left to right). The selectivity index is defined as MIC_*S. aureus*_/MIC_*E. coli*_. Hence, while the mid-length **5h** and **5d** provide the most active and selective compounds against *S. aureus*, for *E. coli*. **Λ-5b** is most promising. Kanamycin (Kan) is included for reference.

The compounds were screened using standard microbial assays to provide minimum inhibitory concentrations (MIC). While most of the compounds showed relatively high activity [(MIC) 1–8 μg ml^–1^] against the Gram-positive microbe *B. subtilis* (168, ATCC 6051 – see Table S3†), perhaps due to the broad antimicrobial sensitivity of the species in liquid,^28^ a range of potencies was observed against the more challenging *E. coli* (TOP10 strain, ATCC PTA-10989) and methicillin resistant *S. aureus* (USA300, ATCC BAA-1717); see Fig. 6. Generally, the enantiomers with ΛFe helicity were more potent than their ΔFe counterparts, particularly against *E. coli*, though the differences were subtle. Compound **Λ-5b** gave a MIC of 2 μg ml^–1^ against *E. coli*; at least as good as kanamycin given that the molecular weight of the metallohelix is *ca.* four times higher. Additionally, the compounds gave minimum bactericidal concentration (MBC)/MIC ratios mostly in the range 1–2 against *E. coli* and *S. aureus*, making them by definition bactericidal [MBC/MIC ≤ 4].^29^ Indeed, the lethal effect of **Λ-5b** upon *E. coli* TOP10 could be observed within 20–40 min. Interestingly, stationary phase *E. coli* cells were much less susceptible to these compounds (see Fig. S12†), suggesting this compound is most toxic to actively growing or dividing cells (*vide infra*). Similar effects have been seen with well-established antibiotics.^30^,^31^ In addition, application of the LIVE/DEAD BacLight™ assay indicated that **Λ-5b** did not cause significant membrane damage to *E. coli* TOP10 at four times the MIC (Table S4†). This demonstrates that **Λ-5b** is not acting as a surfactant.

From Fig. 6 we see that the compound **Λ-5b**, with the shortest intermetallic distance (12.4 Å) has the highest selectivity for *E. coli*, as well as the highest activity. The nearest competitor is the enantiomer **Δ-5b**. In contrast, compounds with mid-range intermetallic distance especially those containing an ether O atom in the bridge, are more selective for Gram-positive *S. aureus*; the enantiomers **5h** and **Λ-5d** which have the highest selectivity are also the most active.

Given the ever more acute clinical need for novel antimicrobial systems active against Gram-negative pathogens, we focused on this area, and the most selective enantiomers **5a** and **5b** were screened against a wider panel of clinically relevant microbes (Table 1). The panel included the notable *Escherichia coli* pathogens: enterohemorrhagic *E. coli* (EHEC) strains of the serotype O157:H7, Sakai (ATCC BAA-460) Δ*stx1-2* and EDL933 (ATCC 700927) Δ*stx1-2*; uropathogenic *E. coli* (UPEC) O6:H1:K2 strain CFT073 (ATCC 700928); an archetypal K12 *E. coli* control strain, MG1655 (ATCC 700926). Added to this panel were examples of Gram-negative *ESKAPE* pathogens isolated from the clinic: *Klebsiella pneumoniae* strains K6 (ATCC 700603) exhibiting extended spectrum β-lactam resistance,^32^ and KP02 (NCTC 13442) exhibiting carbapenem resistance *via* the OXA-48 β-lactamase;^33^ an *Acinetobacter baumannii* reference strain (NCTC 13420) from an MDR UK outbreak;^34^,^35^ *Enterobacter cloacae* strain 684 (NCTC 13405) exhibiting extended spectrum β-lactam resistance *via* the AmpC enzyme.^36^ The compound **Λ-5b** demonstrated particularly good activity against the Enterobacteriaceae tested, especially strains of *E. coli*. Interestingly a striking difference in susceptibility is observed between the two *K. pneumoniae* isolates tested. We also note that none of the cationic metallohelices **5a–h** showed significant activity against *Pseudomonas aeruginosa*, which can deploy a highly negatively charged alginate-based capsule, and correspondingly none of the cationic metallohelices **5a–h** showed significant activity against this pathogen (*e.g.* strains PAO1, ATCC 15692). Overall, on the basis of the above performance, **Λ-5b** was selected for mechanistic study.

**Table 1:** *In vitro* antimicrobial activity (MICs) of metallohelices against Gram-negative strains. Kanamycin and tetracycline controls are also included for reference

Compound	MIC (μg ml^–1^)									
	*E. coli* MG1655	*E. coli* TOP10	UPEC CFT073	EHEC EDL933	EHEC Sakai	*K. pneumoniae* K6	*K. pneumonia* KP02	*E. cloacae* 684	*A. baumannii* 13420	*P. aeruginosa* PAO1
**Λ-5a**	8	4	16	4	16	8	32	64	64	64
**Δ-5a**	32	8	16	16	32	128	128	128	64	128
**Λ-5b**	8	2	4	2	2	4	64	8	64	>128
**Δ-5b**	8	4	4	4	4	8	128	16	64	>128
Kanamycin	2	2	8	4	4	64	>64	2	>64	16
Tetracycline	2	1	4	4	4	16	32	4	>64	4

### EHEC Sakai mutants exhibiting increased tolerance

Resistant strains may emerge when mutants in the bacterial population survive because they are less affected by the drug molecule. The *E. coli* strain EHEC O157:H7 Sakai, a notorious Gram-negative food pathogen, was exposed to inhibitory levels of **Λ-5b** in so-called selection experiments to determine if it was possible to isolate mutants, which had become spontaneously resistant to the antimicrobial compound.

While 17 independent bacterial isolates were recovered, they were found to have achieved only a slight increase in tolerance rather than genuine target site resistance (Table S5†); MICs were only 2–4 times that of the parent strain. Whole genome DNA sequencing (Illumina) was performed on the isolates, and comparison of all the mutant strain genome sequences with that of the original wild-type parent strain allowed the identification of the specific nucleotide changes that gave rise to their increases in MIC. This was achieved using a combination of genome assembly and sequence comparison methods, specifically Bowtie2 and VarScan2 and the web-based Enterobase service,^37^ allowing any single nucleotide polymorphisms (SNPs) and/or small sequence insertions or deletions (Table S5†) to be accurately identified. The mutants could be classed into four sub-types in respect of their differences to the wild-type organism, showing defects in genes involved in maintenance or modification of the outer membrane of the cell.

Two of the sub-types had acquired enzyme-related mutations which would alter the biophysical properties of the LPS outer membrane and thus create tolerance or resistance to the antimicrobial action of the metallohelix: (i) the WaaG enzyme, which has previously been identified as a potential antibiotic target,^38^,^39^ is located at the cytosolic side of the inner membrane and catalyzes the transfer of the first outer-core glucose to the inner core during LPS synthesis for the outer leaflet;^38^,^40^ (ii) *galU* – UTP-glucose-1-phosphate uridylyltransferase is also involved in LPS synthesis.^41^ The LPS O-antigen of EHEC O157:H7 comprises *N*-acetyl-d-perosamine, l-fucose, d-glucose, and *N*-acetyl-d-galactose.^42^ This normally has a defensive role against host antimicrobial peptides. As *N*-acetyl-d-galactose is synthesised from galactose by GalE, GalT, GalK, and GalU,^43^ these *galU* mutants will have defective O-antigen.

The third class of mutants had lost the ability to produce the vitamin B12 transporter protein encoded by the *btuB* gene. All these strains had acquired mutations that introduced a premature stop-codon in the *btuB* gene, which would have the effect of truncating the resulting BtuB protein, rendering it useless. The BtuB protein is responsible for the active translocation of vitamin B12 (cyanocobalamin) across the outer membrane to the periplasmic space. It derives its energy for transport by interacting with the *trans*-periplasmic membrane protein TonB.^44^ Coincidently, BtuB also acts as the receptor for the proteinaceous antibacterial A and E colicins.^45^,^46^ We speculate that this transporter could contribute to entry of **Λ-5b** into the periplasm. It should be noted that while these mutations could represent a defense against influx of the metallohelix, they would have the consequence of lowering the overall fitness of the strain, likely rendering it harmless.

In the fourth mutant sub-type we could identify no chromosomal SNPs. However, in these strains we did note that they had lost the extrachromosomal pO157 virulence plasmid.^47^ This plasmid is important as genes encoded on it are at least partly responsible for the very high virulence of this *E. coli* strain, so again these mutants would be incapable of causing disease. This finding is also intriguing because it has been previously reported that this plasmid has a very high level of inheritance stability,^46^ suggesting that the compound is interfering with plasmid stability. Furthermore, we note that pO157 encodes genes that are predicted to be involved in LPS modification (genes pO157p79–pO157p82).^48^,^49^ Moreover, products of the plasmid encoded *ecf* and *lpxM* genes also affect membrane fatty acid composition and lipid A structure.^50^ Thus we speculate that the compound not only destabilised the plasmid replication and/or inheritance mechanisms, but also provided an evolutionary selection pressure to lose the plasmid altogether, leading to limited increase in tolerance.

### EHEC Sakai transcriptomic response to compound **Λ-5b**

In addition to the experiments described above, which investigate genetic mutations that lower susceptibility to the compound, we also wanted to consider how a population of wild-type bacteria responds to intoxication. A very effective way to do this is to expose the bacteria to sub-lethal levels of the compound, and subsequently look at how the cell changes gene expression patterns in response. This is done using a so-called transcriptomic analysis (RNAseq), wherein all the mRNA gene transcripts from treated cells are compared to those from an untreated culture using next-generation sequencing techniques. Transcriptomic studies such as these can provide clues as to the target site and mechanism of action of novel drug molecules. Therefore, we performed a transcriptomic analysis of bacteria that had been exposed to **Λ-5b** for a limited time at MIC/4 in order to capture the immediate response rather than changes associated with cell death. Using this approach and paired sample analysis we were able to successfully define a limited number of statistically sound expression changes; an increase in transcription of 48 genes and a decrease in only 20. The full dataset can be seen in ESI file S1.† We will discuss here the key findings.

### Cationic antimicrobial peptide resistance and membrane maintenance

In *E. coli* the presence of compound **Λ-5b** induced expression of genes for sensors, regulators and LPS modification, known to be upregulated in response to attack by natural cationic antimicrobial peptides (CAMPs). These include the genes for PhoPQ two component (2C) sensor/regulator pair^51^ and MgrB, which modulates the PhoQ sensor response range.^52^ This 2C system is known to up regulate genes for LPS modification in response to CAMP intoxication,^53^ and indeed we also see an up regulation in transcription of the arnB gene, which encodes UDP-4-amino-4-deoxy-l-arabinose-oxoglutarate aminotransferase. This is an enzyme which acts in the lipid A modification pathway, causing an increase in the surface positive charge of the cell, thus reducing interaction with CAMPs, as demonstrated by resistance to polymyxin.^52^ Several other PhoPQ regulated genes are also up regulated upon exposure to **Λ-5b**. These include genes for the proteins PhoE (an outer membrane phosphoporin allowing for passive diffusion of small molecules), MgtA (a magnesium-transporting ATPase) and RstAB (a 2C sensor/regulator system which is itself regulated by Mg^2+^ levels). It should be noted that the *phoPQ* genes are also under the control of a Mg^2+^ responsive promoter, suggesting cross talk between several stimuli.^54^ While the PhoE porin showed transcriptional up regulation, we note that a second porin, OmpW showed a significant down regulation. Under microaerobic conditions OmpW functions in the transport of hydrophobic molecules across the outer membrane.^55^ It also represents a receptor for the antimicrobial protein colicin S4.^56^ As for the B12 gene mentioned above, this porin could be responsible for influx of the compound.

Increased transcription of the gene for the YbjG undecaprenyl pyrophosphate phosphatase is also relevant as YbjG family proteins can confer bacitracin resistance.^57^ Accordingly, disruption of ybjG causes increased bacitracin sensitivity, while overexpression causes increased resistance to bacitracin. Interestingly we also see an increase in transcription of the gene for FabR which is known to be involved in regulating genes responsible for unsaturated fatty acid (UFA) biosynthesis.^50^ By controlling UFA production, FabR may indirectly influence the physical properties of the membrane bilayer. Two further, tightly linked genes, also relevant to CAMP resistance that show an increase in transcription, are those for the EptA phosphoethanolamine transferase and its regulator sensor protein, PmrB. Like PhoPQ, the PmrAB 2C system is also known to regulate resistance to CAMPs.^58^ The PmrAB system upregulates EptA, which in turn catalyzes the addition of a phosphoethanolamine moiety to the KDO component of lipid A,^59^ facilitating resistance to polymyxin B. Interestingly the sensor domain of PmrB is known to respond to several stimuli including acidic pH, which provides a link to another group of up regulated genes, involved in coping with acid stress. These observations highlight the complex interrelationships and overlaps between different forms of stress-response often seen in bacteria.

### Acid stress response

It is interesting that a link between transcriptional acid stress response and resistance to various membrane active environmental stresses, including polymyxin B have been observed in other bacteria suggesting a mechanistic relationship.^60^,^61^ Indeed, an upregulated gene we observed, involved in both acid response and LPS synthesis is lpxT. LpxT is an enzyme, which is induced under acidic-aerobic conditions, that catalyses the phosphorylation of lipid A.^62^ Furthermore, also of relevance here is the upregulation of the gene asr, which is dependent upon PhoB and RstA (see above), and facilitates an acid-induced protective response in cells exposed to very low pH.^63^,^64^ Other upregulated genes observed that are known to be involved with acid stress response are those encoding for EfeB (a periplasmic deferrochelatase which extracts iron from exogenous heme), YdeP (an oxidoreductase) and RibB (involved in riboflavin biosynthesis in low pH). Note, we also observed the upregulation of response genes for other forms of stress including the heat shock Hsp90 and tellurite resistance TerW proteins. Upregulated oxidative stress response genes observed include; sodA superoxide dismutase, yeaR which is induced by nitric oxide, napF a ferredoxin-type protein predicted to play a role in the oxidative stress response and tatD (mttC) a DNase potentially involved with the repair of H_2_O_2_ induced DNA damage.

### Bacterial sub-cellular compound localization

We sought to investigate whether these large metallohelices could freely enter bacterial cells using fluorescence microscopy. A simple alkyne derivative of compound **Λ-5b***i.e.***Λ-5b′** (see ESI methods†) was synthesised for use in fluorescent “click” labelling chemistry using an Alexa Fluor® 488 azide (AF-488). Antimicrobial assays confirmed that **Λ-5b′** remained active against *E. coli*.

**Fig. 7:**
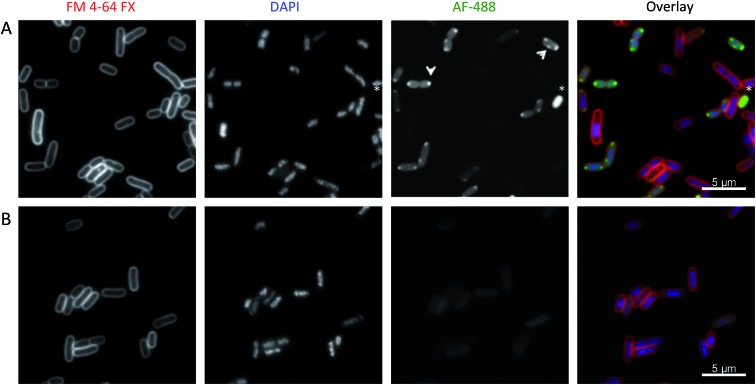
Sub-cellular localization of **Λ-5b′** in EHEC Sakai. Exponentially growing EHEC Sakai cells were treated with either (A) 8 μg ml^–1^**Λ-5b′** or (B) methanol and stained for membrane (FM 4-64 FX), nucleic acid (DAPI) and the **Λ-5b′** (*via* click reaction with AF-488 azide). The arrows indicate the punctate localisation pattern of **Λ-5b′**. The asterisk indicates an example of a bacterial cell with prominent **Λ-5b′** but lack of DAPI staining.

When cultured bacteria cease replication due to exhausting all the nutrients from the medium, they enter a quiescent state referred to as the stationary phase. We observed very little fluorescence staining of **Λ-5b′** in stationary phase *E. coli*, with the exception of a few cells with apparently compromised membranes. In contrast the compound was observed to enter the cytoplasm of exponentially growing cells and concentrate in punctate regions near the poles (Fig. 7A). Occasionally, the puncta were also positioned laterally. Importantly, we did not observe **Λ-5b′** localising preferentially in the membrane and the levels of staining for **Λ-5b′** were not homogeneous among cells suggesting differential uptake of the compound.

Several structures and phenomena are known to be associated with the poles of *E. coli* cells; these include anionic phospholipids such as cardiolipin that enrich in microdomains at regions of high negative membrane curvature^65^,^66^ and chromosome partitioning machinery.^67^–^69^ It is interesting to note that Keene and Collins^70^ have observed the accumulation of antimicrobial dinuclear ruthenium tetracationic polypyridyl complexes at the cell poles by luminescence microscopy of *E. coli*. They ascribe this to localisation in the RNA of ribosomes, following imaging studies in the presence of antibiotics that are known to disrupt RNA/ribosomal distributions. However, we observed no signs of protein synthesis inhibition in our transcriptomic data.

A control imaging study with cells treated without compound **Λ-5b′** showed no staining with AF-488 (Fig. 7B). Interestingly, we noticed that cells which stained brightly for the test compound had reduced fluorescence on staining with DAPI – a DNA binder. This was confirmed by image analysis, which demonstrated bimodal frequency distribution of pixel mean intensity in the DAPI and AF-488 channels (Fig. S13†), suggesting competitive DNA binding.

### DNA interactions and enzyme inhibition *in vitro*

Following our observations above on DAPI stain exclusion, we decided to look directly at the interaction of **Λ-5b** with DNA. In addition, we note previous studies have demonstrated interactions of related metallohelices with naked DNA.^23^,^71^,^72^ Ethidium bromide displacement studies on calf thymus DNA (CT-DNA) by **Λ-5b**, gave an apparent binding constant *K*_app_ of 1.2 × 10^8^ M^–1^ (±1.2 × 10^7^ M^–1^). Concentration-dependent increases in CT-DNA melting temperature [Δ*T*_m_ of +4.3(0.3) °C and +6.5(1.8) °C, at DNA base to metallohelix ratio 20 : 1 and 10 : 1 respectively] were observed.

Linear dichroism (LD) studies show that only **5a** and **5b** (Fig. S9†) bind in an aligned *i.e.* not simply electrostatic manner with DNA – probably in the major groove.^73^ Correspondingly, these LD studies also indicate concentration-dependent structural deformation of DNA, and this was confirmed by atomic force microscopy on linearised plasmid pSP73 DNA (Fig. S10†).

**Fig. 8:**
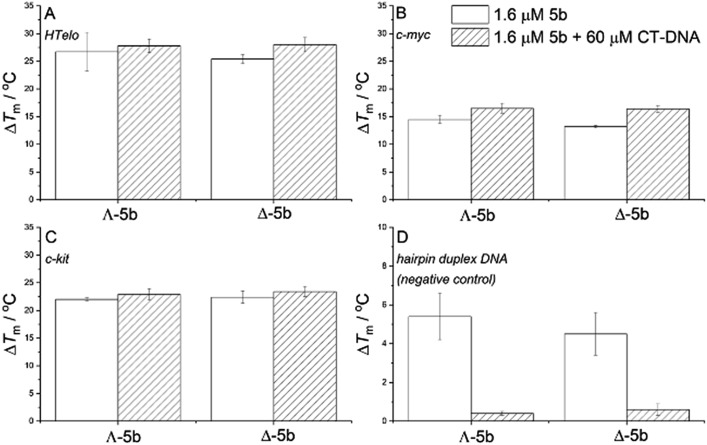
Selective stabilization of DNA G-quadruplexes over duplex DNA. FRET melting assay of fluorophore-labelled quadruplex-forming oligonucleotides (A) HTelo, (B) *c-myc*, (C) *c-kit*, and (D) the labelled oligonucleotide F26T_ds-hairpin, which does not form a quadruplex structure (negative control). Δ*T*_m_ values determined by FRET upon addition of **5b** (1.6 μM) in the absence and in the presence of 60 μM CT-DNA show strong preferential quadruplex binding in the presence of excess duplex CT-DNA (Δ*T*_m_ unchanged).

Since G-quadruplex motifs – four-stranded structures formed by guanine-rich DNA – have been shown to be important in the regulation of the transcription of certain genes and potentially other processes in bacteria including *E. coli*,^74^–^76^ we investigated their interactions with enantiomers **5b**. A FRET melting assay^77^ was used to assess the change in stability of G-quadruplex structures on addition of a metallohelix (Fig. 8). Three representative fluorophore labelled oligonucleotides were used, plus a negative control hairpin duplex which does not form a G-quadruplex motif. Further, by performing these studies in the presence and absence of an excess of calf thymus DNA (ctDNA) we were able to assess the selectivity of the compounds for binding the G-quadruplex motif over major groove binding (*vide supra*). In Fig. 8A–C we see that G-quadruplex structures were significantly stabilised by both enantiomers, as indicated by the change in melting temperatures (Δ*T*_m_) of *ca.* +26, 22 and 14 °C respectively, and that this was not affected by addition of 60 μM ctDNA. This indicates substantial selectivity. In Fig. 8D we see also that while the thermal stability of the control hairpin duplex was increased by both enantiomers, the effect disappeared in the presence of 60 μM ctDNA. Interestingly we subsequently demonstrated that *in vitro***Λ-5b** did not affect the endonuclease cleavage of plasmid pSP73KB DNA by EcoRI or DNA transcription (data not shown). However, the compound was shown to inhibit both relaxation and supercoiling of pBR322 plasmid DNA by *E. coli* topoisomerase I and DNA gyrase respectively (Fig. S11†).

## Conclusions

In this work we present a range of self-assembled helical metallo-organic compounds able to selectively kill – at low doses – a range of bacterial pathogens, including clinically problematic species such as members of the ESKAPE panel of microbes. A particularly useful property of this class of molecule is the ability to change the handedness, size, shape and charge-distribution in the structure by selection of sub-components for the self-assembly. Many other avenues are available including changes to substituent groups for modification of lipophilicity and functionality. This will also facilitate investigation of the basis of the intriguing specificities observed.

The cationic nature of the metallohelices would predispose them, as for CAMPs, to interaction with bacterial membranes, which are negatively charged. Given the size and charge of **Λ-5b** we may have expected to see it accumulate across the outer surface of the membrane or cause membrane disruption, like some CAMPs. However, membrane integrity is maintained and bacterial localisation experiments revealed that **Λ-5b′** was able to enter the cell cytoplasm, partially accumulating at distinct foci, typically at the poles of cells.

Given that microdomains of cardiolipin and chromosome partitioning machinery is known to accumulate at polar foci in cells,^67^–^69^ it is reasonable to suggest that **Λ-5b** could be interacting with either of these components. The latter would be consistent with our DNA binding studies. We showed that **Λ-5b** has a very high affinity for binding G-quadruplex DNA over double stranded DNA. The role of tertiary structure and the conformation of DNA during replication is highly complex^78^ and so it is difficult at this juncture to posit a specific point at which the metallohelix may be interfering. With this in mind we note this compound is able to inhibit the activity of both DNA gyrase and topoisomerase I *in vitro*. DNA topoisomerase enzymes play crucial roles in the supercoiling and relaxation of DNA, which is essential for both chromosomal replication and partitioning. It is perhaps relevant here to recall that the compound only shows activity against exponentially growing cells. Furthermore, **Λ-5b** also caused the curing of the pO157 plasmid in one sub-class of the tolerance mutants. pO157 is a single copy number plasmid, and like the chromosome itself, it relies upon a ParAB-like partitioning system (SopAB), which is an inner membrane associated mechanism.

When applied at sub-MIC levels, we observe a transcriptomic response consistent with the normal response of *E. coli* to a natural CAMP. The cell must be able to sense the influence of the metallohelix, possibly on membrane properties, leading to the activation of the various two-component sensor/regulator pathways, acid response pathways and subsequent attempts by the cell to lower the net negative charge of the surface.

The Gram negative outer membrane can act as an efficient intrinsic permeability barrier to the egress of certain antimicrobial agents such as vancomycin.^79^ In addition, surface charge modification, as indicated here, is a well-established mechanism of resistance to cationic antimicrobial peptides, such as the action of the ArnB enzyme on LPS to reduce the interaction of polymyxin.^52^ Furthermore, alterations in membrane permeability have also been established as a more general mechanism to control the influx of antibiotics.^80^

These defence responses by *E. coli* to the metallohelices are nevertheless insufficient at the MIC. Our inability to isolate fully resistant “target site” mutants also strongly suggests that the compound impacts on multiple structures or pathways.

The metallohelices thus represent functional emulators of key properties of their more exquisitely structured natural counterparts. Notably they are of similar size to short CAMPs but remain folded thereby exhibiting “patchy” hydrophobic regions on the surface. We propose that as highly stable and fully non-natural chemical entities, containing no amino acids or peptide bonds, the evolution or acquisition of novel enzyme functions that could mitigate the action of the metallohelix may well be rare events or entail high fitness costs for bacteria.

We note that the first report of antimicrobial activity of metal complex ions appeared in 1952,^81^ and arguably the most promising developments in this area^82^ have been the closely related ruthenium-based intercalators and oligonuclear ruthenium systems.^83^–^85^ Perhaps the ability to make optically pure compounds without the need for resolution, and the absence of toxic metals, further adds to attractiveness of the Fe-based metallohelices in this report.

Taken as a whole our findings suggest that the development of peptide-inspired scaffolds that emulate the structural features of CAMPs but have lower cost of synthesis and perhaps better pharmacological properties, is a realistic approach for the discovery of new antimicrobial drugs.

## Experimental section

### Synthesis of water soluble metallohelices

The appropriate optically pure diamine (3.0 eq.) and 2-pyridinecarboxaldehyde (6.0 eq.) were dissolved in methanol (50 ml) and stirred for 2 h at ambient temperature. Anhydrous iron(ii) chloride (2.0 eq.) was added, and an immediate color change to deep purple was observed. The solution was then heated at reflux (80 °C) for 48 hours. After filtering, the solvent was removed under reduced pressure to give the desired product as a dark purple solid, which was dried overnight at 50 °C *in vacuo*. Detailed synthesis and characterization methods can be found in the ESI and methods.†

### DNA quadruplex binding FRET melting assay

0.4 μM annealed double-FRET-labelled synthetic oli-godeoxyribonucleotides (Eurofins Genomics, Ebersberg, Germany) were mixed with 1.6 μM metallohelix in the absence of CT-DNA, and in the presence of 60 μM CT-DNA. Experiments with F21T_HTelo and F26T_ds-hairpin were performed at 40 mM K^+^ concentration (additional 30 mM KCl). Measurements were performed on a real-time PCR instrument RotorGene 6000 (Corbett Research) *λ*_exc_ = 470 ± 10 nm, *λ*_em_ = 510 ± 5 nm, at intervals of 0.7 °C min^–1^.

### Determination of minimum inhibitory concentrations (MIC) and minimum bactericidal concentrations (MBC)

The bacterial strains used in this study are listed in Table S2.† For MIC determination the standard broth microdilution method was employed, in agreement with the Clinical and Laboratory Standards Institute (CLSI) guidelines M07-A9 and M100-S24. Cation-adjusted Mueller Hinton Broth (CAMHB) was used as the media and a 1280 μg ml^–1^ stock solution of each metallohelix was prepared in water containing 10% methanol. **Λ-5b** and **Δ-5b** MICs were independently validated using an extended range of 0.007–256 μg ml^–1^. Determination of MBCs was carried out for strain/compound pairings where an MIC ≤ 128 μg ml^–1^ was determined, immediately following that assay. For each culture with compound concentration in the range 128 μg ml^–1^ to the MIC (inclusive), 100 μl of the bacteria/compound mix was recovered from the microtitre plate for analysis. Cell and/or debris was collected from samples by centrifugation and resuspended in 100 μl sterile PBS, which was streaked onto a sterile antimicrobial-free LB/agar plate. Upon overnight incubation (37 °C), plates were inspected and the MBC was determined to be the lowest concentration of compound at which this dilution/culturing assay showed no visible signs of bacterial growth. This was performed at least in duplicate for each pairing of compound concentration and bacterial strain.

### Isolation of tolerant mutants

7 independent biological replicates of EHEC Sakai were grown in CAMHB, and 25 μl of each culture was applied to MH agar containing 80 μg ml^–1^ of **Λ-5b** (a high concentration was used as the compound does not appear to diffuse well in agar) and incubated at 37 °C overnight. Four colonies from each replicate were re-streaked onto MH agar both with and without supplementation with **Λ-5b**. These were used to seed fresh cultures in MH broth to a cell density of 5 × 10^5^ cfu ml^–1^, incubated with 2 μg ml^–1^**Λ-5b** (the Wild Type (WT) MIC). The MIC of the **Λ-5b** for the isolates was measured and the bacteria were studied by whole-genome sequencing.

### Whole genome sequencing

Genomic DNA libraries were prepared using the Illumina Nextera® XT kit using 1 ng of input DNA. Following PCR clean up, the DNA concentration of each library was measured using the Qubit® high-sensitivity dsDNA and the libraries were normalised to 4 nM and pooled together. The pool was prepared for loading following the Illumina Nextera® XT guidelines. Sequencing was performed using a MiSeq Reagent Kit v2 (500-cycles) on an Illumina MiSeq™ instrument.

### Extraction and purification of microbial RNA

EHEC Sakai were incubated in CAMHB with a sub-MIC dose of metallohelix to a final concentration of 0.5 μg ml^–1^ for 40 min at 37 °C. Samples were added to RNAprotect reagent (Qiagen) and incubated for 5 min at room temperature. Enzymatic lysis was performed using TE buffer with proteinase K and 1 mg ml^–1^ lysozyme. RNA was then purified using the miRNeasy kit (Qiagen) with the inclusion of a double on-column DNA digestion. Absence of contaminating DNA was confirmed by PCR using primers for the *E. coli* 16S ribosomal subunit gene. RNA integrity was verified using the RNA 6000 pico kit (Agilent) on the Agilent 2100 Bioanalyzer. To deplete rRNA, the Ribo-Zero™ bacterial rRNA removal kit (Illumina) was used with an input RNA of 4 μg per sample.

### Generation of cDNA libraries and sequencing

rRNA-depleted samples were used as input for cDNA library preparation using the Illumina TruSeq™ stranded mRNA kit with a slightly modified library preparation protocol. cDNA libraries were quantified using the Qubit® High Sensitivity DNA assay kit and fragment sizes were determined using the High Sensitivity DNA kit on the Agilent 2100 Bioanalyzer. The libraries were normalised to 4 nM and pooled together. Paired-end sequencing was performed using two Miseq reagent kits v3 (150-cycle) on an Illumina MiSeq™ sequencer.

### Bacterial sub-cellular compound localization

Overnight culture of EHEC Sakai was diluted in cation-adjusted Mueller Hinton broth and grown to mid-exponential (OD_600_ = 0.52) in order to image dividing cells. For stationary phase cells, a 24 h culture of EHEC Sakai at an OD_600_ of 2.9 was used. The cultures were incubated with **Λ-5b′** for 30 min at 37 °C. Ten minutes before the end of the incubation period, 5 μg ml^–1^ FM™ 4-64FX (Invitrogen) was added to stain the cell membrane. For visualisation of the target compound we used the Click-IT cell reaction buffer kit (Invitrogen) as per the manufacturer's instructions. Finally, cells were stained with 1 μg ml^–1^ DAPI for 1 min, washed with PBS, and imaged using a Leica DMi8 inverted microscope.

## Conflicts of interest

There are no conflicts to declare.

## Supplementary Material

Supplementary information

Supplementary information

Crystal structure data
