# Supplementary material for: Metallohelices that kill Gram-negative pathogens using intracellular antimicrobial peptide pathways
Source: Chem Sci. 2019 Sep 5;10(42):9708–20. doi: 10.1039/c9sc03532j (PMC6977464; doi:10.1039/c9sc03532j)
Supplement: Supplementary file 1 [file SC-010-C9SC03532J-s001.pdf]

Electronic Supplementary Material (ESI) for Chemical Science.
This journal is © The Royal Society of Chemistry 2019

Supporting information

Metallohelices that kill Gram-negative pathogens using intracellular antimicrobial peptide pathways

Daniel H Simpson[1†], Alexia Hapeshi[2†], Nicola Rogers[1†], Viktor Brabec[4], Guy J Clarkson[1], David J. Fox[1], Ondrej Hrabina, Gemma L Kay[2], Andrew K King[1], Jaroslav Malina, Andrew D Millard[2], John Moat[3], David I Roper[3]*, Hualong Song[1], Nicholas R Waterfield[2]*, and Peter Scott[1]*.

[1] Department of Chemistry, University of Warwick, Gibbet Hill Road, Coventry, CV4 7AL, UK

[2] Warwick Medical School, University of Warwick, Coventry, CV4 7AL, UK

[3] School of Life Sciences, Gibbet Hill Campus, University of Warwick, Coventry, CV4 7AL, UK

[4] The Czech Academy of Sciences, Institute of Biophysics, Kralovopolska 135, CZ-61265 Brno, Czech Republic

*Correspondence to: peter.scott@warwick.ac.uk

†These authors contributed equally.

Contents

1. Synthesis & Characterization S3
1.1 Solvents and chemicals S3
1.2 Instrumentation S3
1.3 Synthetic methods S4
1.4 Optical purity of amines: synthesis and analysis of Mosher amides S45
1.5 Electronic spectra S47
1.6 Thermogravimetric analysis S50
1.7 Crystallography S52
1.8 Stability testing S62
2. DNA interaction assays S64
2.1 Ethidium bromide displacement S64
2.2 UV melting assays S64
2.3 Linear dichroism spectroscopy S64
2.4 Atomic Force Microscopy S66
2.5 DNA Quadruplex binding FRET melting assay S68
2.6 Topoisomerase I and gyrase inhibition assays S68
3. Microbiology methods S71
3.1 Determination of minimum inhibitory concentrations (MIC) and minimum bactericidal concentrations (MBC) S71
3.2 Λ-5b activity in stationary phase cells S72
3.3 *BacLight*™ LIVE/DEAD assay – further details S74
3.4 Isolation of tolerant mutants – further details S76
3.5 DNA extraction and whole genome sequencing S78
3.6 Extraction and purification of microbial RNA S78
3.7 Generation of cDNA Libraries and sequencing S79
3.8 Bacterial sub-cellular compound localization- further details S79
4. Bioinformatics S83
4.1 Whole genome analysis S83
4.2 Transcriptomic analysis S84
Data S1. (separate file) S84
5. References S85

1. Synthesis & Characterization

1.1 Solvents and chemicals

All solvents and chemicals purchased from commercial sources (Sigma-Aldrich, Acros, Fisher Scientific, Alfa Aesar or Invitrogen) were used without further purification unless otherwise stated. Sodium hydride dispersions in mineral oil were placed in a Schlenk vessel under an inert atmosphere and washed three times with diethyl ether to remove the oil, then dried and stored under argon in an MBraun dry box. Where necessary solvents were dried by heating to reflux for 3 d under dinitrogen over the appropriate drying agents (potassium for tetrahydrofuran, and calcium hydride for methanol and DCM) and degassed before use. Tetrahydrofuran was additionally pre-dried over sodium wire. Dried solvents were stored in glass ampoules under argon. Deuterated solvents were purchased from Sigma-Aldrich or Cambridge Isotope Laboratories and pre-dried over molecular sieves (3 Å for methanol, dimethyl sulfoxide and acetonitrile; 4 Å for chloroform), for 24 h prior to use. Perchlorate salts pose an explosion risk, particularly when heated, and were therefore only used on a small scale and never exposed to excess heat.

(*R*)- and (*S*)-2-phenylglycinol[1] was synthesised as previously described; see also dx.doi.org/10.1039/SP275. Details for the synthesis of some other literature compounds are given below where the method is modified.

1.2 Instrumentation

NMR spectra were recorded on Bruker Advance III HD300/400/500 spectrometers. Routine NMR assignments were confirmed by ^{1}H-^{1}H (COSY) and ^{13}C-^{1}H (HSQC) correlation experiments where necessary. The spectra were internally referenced using the residual protio solvent ($CDCl_3$, CD_3CN *etc.*) resonance relative to tetramethylsilane (δ = 0 ppm). ESI mass spectra were recorded in a methanol/water mixture (4:1) on either an Agilent Technologies 1260 Infinity spectrometer or a Bruker Daltonics MicroTOF spectrometer. Fourier-transformed infra-red (FTIR) spectra were measured using a Bruker Alpha-P FTIR spectrometer (undergraduate laboratory, University of Warwick). Elemental analyses were performed by Medac Ltd. Chobham, Surrey, UK GU24 8JB. Optical rotation measurements were performed on a Perkin Elmer Polarimeter 341 by Warwick Analytical Services, Coventry, UK. In all cases the

following parameters were used: solvent methanol, temperature 21 °C, pathlength 100 mm, wavelength 589 nm.

1.3 Synthetic methods

***bis*-4-(Bromomethyl)phenyl ether[2]**

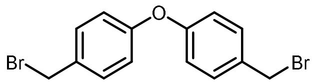

Diphenyl ether (5.67 g, 33.3 mmol) and paraformaldehyde (4.0 g, 0.13 mol, 4 eq.), were suspended in a solution of 33 wt% HBr in glacial acetic acid (36 ml). The mixture was stirred at ambient temperature for 48 h. The white solid formed was collected by filtration, washed with water (100 ml) and 2:1 *n*-hexane/ethyl acetate (80 ml), and dried in air. The product was recrystallised from hot toluene/*n*-hexane, to give a white crystalline solid, which was collected by filtration and dried overnight at 50°C *in vacuo*.

Yield: 6.74 g, 18.9 mmol, 57 %.
^{1}H NMR (300 MHz, 298 K, $CDCl_3$): δ_H 7.37 (4H, d, $^{3}J_{HH}$ = 8.5 Hz, Ph), 6.97 (4H, d, $^{3}J_{HH}$ = 8.5 Hz, Ph), 4.51 (4H, s, CH_2).
$^{13}C\{^{1}H\}$ NMR (75 MHz, 298 K, $CDCl_3$): δ_C 157.1 (Ph), 133.1 (Ph), 130.8 (Ph), 119.3 (Ph), 33.3 (CH_2).

MS (CGMS): m/z 277.7 $[M\text{-}Br]^+$, 275.7 $[M\text{-}Br]^+$, 197.4 $[M\text{-}2Br+H]^+$.
FTIR: ν cm^{-1} 1592 w, 1495 m, 1439 w, 1231 s, 1198 s, 1160 m, 1088 m, 1015 w, 869 m, 860 m, 839 s, 813 m.
Elemental analysis found (calculated for $C_{14}H_{12}Br_2O$): % C 46.76 (47.23) H 3.35 (3.40) N <0.1 (0).

***bis*-4-(bromomethyl)phenyl sulphide[2]**

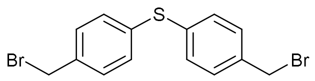

Diphenyl sulfide (2.8 ml, 3.1 g, 16.6 mmol, 1.0 eq.) and paraformaldehyde (1.8 g, 59.9 mmol, 3.6 eq.) were added to a solution of 33 wt% HBr in glacial acetic acid (18 ml). The mixture was stirred under reflux at 50 °C overnight. Upon cooling, the addition of water (100 ml) caused a white precipitate to form, which was collected by filtration, washed with water (100 ml) and 2:1 *n*-hexane/ethyl acetate (80 ml), and dried in air. The product was recrystallised from hot toluene/*n*-hexane, to give a white crystalline solid, which was collected by filtration and dried overnight at 50°C *in vacuo*.

Yield: 4.01 g, 10.8 mmol, 65 %.
^{1}H NMR (500 MHz, 298 K, $CDCl_3$): δ_H 7.34-7.29 (8H, m, Ar), 4.47 (4H, s, CH_2).
$^{13}C\{^{1}H\}$ NMR (125 MHz, 298 K, $CDCl_3$): δ_C 137.0 (Ph), 136.0 (Ph), 131.4 (Ph), 130.1 (Ph), 33.0 (CH_2).
MS (CGMS): m/z 372.9 $[M+H]^+$, 293.7 $[M-Br]^+$, 292.3 $[M-Br]^+$, 213.2 $[M-2Br+H]^+$.
FTIR: ν cm^{-1} 1487 w, 1222 m, 1196 m, 1086 w, 1016 w, 839 s, 804 w.
Elemental analysis found (calculated for $C_{14}H_{12}Br_2S$): % C 44.91 (45.19), H 3.30 (3.25), N <0.1 (0).

***bis*-4-(bromomethyl)phenylmethane[3]**

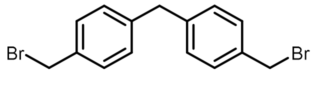

Diphenylmethane (5.0 g, 29.7 mmol, 1.0 eq) and paraformaldehyde (5.35 g 0.178 mol, 6.0 eq.) were suspended in a mixture of aqueous 48 wt% HBr solution (80 ml) and glacial acetic acid (25 ml). Tetradecyltrimethylammonium bromide (0.16 g) was added and the suspension was stirred under reflux at 125 °C overnight. Upon cooling, the yellow solid formed was collected by filtration, washed with water (100 ml) and dried in air. The product was recrystallised from hot

toluene/*n*-hexane, to give a white powder, which was collected by filtration and dried overnight at 70 °C *in vacuo*.

Yield: 3.85 g, 10.9 mmol, 37 %.
^{1}H NMR (300 MHz, 298 K, $CDCl_3$): δ_H 7.32 (4H, d, $^3J_{HH}$ = 7.4 Hz, Ph), 7.15 (4H, d, $^3J_{HH}$ = 7.4 Hz, Ph), 4.48 (4H, s, CH_2Br), 3.96 (2H, s, CH_2).
$^{13}C\{^1H\}$ NMR (75 MHz, 298 K, $CDCl_3$): δ_C 141.2 (Ph), 135.9 (Ph), 129.5 (Ph), 129.4 (Ph), 41.5 (CH_2), 33.6 (CH_2Br).
MS (CGMS): m/z 275.7 $[M-Br]^+$, 273.7 $[M-Br]^+$.
FTIR: ν cm^{-1} 1511 w, 1432 w, 1416 w, 1226 m, 1198 s, 1022 w, 864 m, 823 s, 765 m, 721 m, 715 s.
Elemental analysis found (calculated for $C_{15}H_{14}Br_2$): % C 51.53 (50.88), H 3.96 (3.99), N <0.1 (0).

1,2-*bis*-4-(bromomethyl)phenylethane

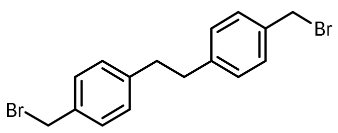

Bibenzyl (5.42 g, 29.7 mmol, 1.0 eq) and paraformaldehyde (5.35 g 0.178 mol, 6.0 eq.) were suspended in a mixture of aqueous 48 wt% HBr solution (80 ml) and glacial acetic acid (25 ml). Tetradecyltrimethylammonium bromide (0.16 g) was added and the suspension was stirred under reflux at 125 °C overnight. Upon cooling, the yellow solid formed was collected by filtration, washed with water (100 ml) and dried in air. The product was recrystallised from hot DCM/*n*-hexane, to give a yellow powder, which was collected by filtration and dried overnight at 50 °C *in vacuo*.

Yield: 1.95 g, 5.3 mmol, 18 %.
^{1}H NMR (300 MHz, 298 K, $CDCl_3$): 7.31 (4H, d, $^3J_{HH}$ = 7.3 Hz, Ph), 7.17 (4H, d, $^3J_{HH}$ = 7.3 Hz, Ph), 4.49 (4H, s, CH_2Br), 2.91 (4H, s, CH_2).

$^{13}C\{^1H\}$ NMR (75 MHz, 298 K, $CDCl_3$): δ_C 142.1 (Ph), 135.7 (Ph), 129.3 (Ph), 129.0 (Ph), 37.5 (CH_2), 33.8 (CH_2Br).
MS (CGMS): m/z 289.8 $[M-Br]^+$, 287.8 $[M-Br]^+$, 243.8 $[M-2Br+Cl]^+$, 184.9 $[C_8H_8Br]^+$, 182.9 $[C_8H_8Br]^+$, 139.5 $[C_8H_8Cl]^+$, 104.5 $[C_8H_8]^+$.
FTIR: ν cm^{-1} 1611 w, 1511 m, 1437 w, 1418 m, 1226 s, 1211 s, 1200 s, 1081 w, 1017 w, 830 s, 749 m.
Elemental analysis found (calculated for $C_{16}H_{16}Br_2$): % C 52.88 (52.21), H 4.46 (4.38), N <0.1 (0).

2,8-*bis*(bromomethyl)dibenzofuran

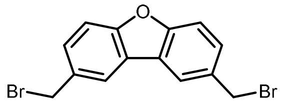

Dibenzofuran (1.0 g, 5.9 mmol, 1.0 eq) and paraformaldehyde (0.78 g, 26.0 mol, 4.38 eq) were suspended in a solution of 33 wt% HBr in glacial acetic acid (10 ml). 90 wt% phosphoric acid (5 ml) was added and the mixture stirred under reflux at 65 °C for 1 h, then at ambient temperature overnight, before cooling. The reaction contents were added to ice-cold water (150 ml), and the white solid collected by filtration and dried in air. The crude product was dissolved into a minimal volume of hot toluene, before adding an excess (~100 ml) of *n*-hexane. The product was recrystallised from hot toluene/*n*-hexane, to give a white powder, which was collected by filtration and dried overnight at 50°C *in vacuo*.

Yield: 1.00 g, 2.8 mmol, 48 %.
^1H NMR (300 MHz, 298 K, $CDCl_3$): δ_H 7.98 (2H, s, Ph), 7.56-7.48 (4H, m, Ph), 4.69 (4H, s, CH_2).
$^{13}C\{^1H\}$ NMR (100 MHz, 298 K, $CDCl_3$): δ_C 156.6 (Ph), 132.9 (Ph), 128.9 (Ph), 124.4 (Ph), 121.6 (Ph), 112.3 (Ph), 34.0 (CH_2Br).
MS (CGMS): m/z 275.7 $[M-Br]^+$, 274.3 $[M-Br]^+$, 229.8 $[M-2Br+Cl]^+$, 194.9 $[M-2Br]^+$.
FTIR: ν cm^{-1} 1601 w, 1486 w, 1458 w, 1219 s, 1205 s, 1187 s, 1126 m, 1024 m, 882 m, 822 s, 736 m.

Elemental analysis found (calculated for $C_{14}H_{10}Br_2O$): % C 47.16 (47.50), H 2.88 (2.85), N <0.1 (0).

(*R*,*R*)-α,α'-*bis*(2-amino-2-phenylethoxy)-*p*-xylene [(*R*,*R*)-3a]

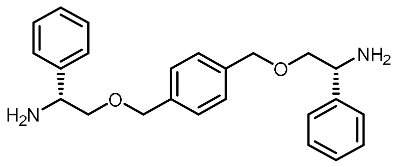

Under inert atmosphere, (*R*)-2-phenylglycinol (2.0 g, 14.6 mmol, 2.2 eq.) was dissolved in anhydrous THF (50 ml), to which 1 ml (1.11 g, 5.0 mmol, 0.8 eq.) [15]-crown-[5] was added by injection. The solution was then added dropwise to neat sodium hydride (0.72 g, 31.3 mmol, 4.8 eq.). The effervescent mixture was carefully placed under static vacuum and stirred at ambient temperature for 1 h. A solution of α,α'-dibromo-*p*-xylene (1.73 g, 6.6 mmol, 1 eq.) in anhydrous THF (40 ml) was then added dropwise. The reaction mixture was then stirred under static vacuum; for 1 h at ambient temperature, then 5 h at 65 °C. The bright yellow reaction mixture was allowed to cool before quenching with 2:1 saturated KCl aq./water (60 ml). The crude product was extracted using diethyl ether (3 × 100 ml), dried over sodium sulfate, filtered through celite, and the solvent removed under reduced pressure to leave a yellow oil. This crude product was purified by Kügelrohr distillation (150 °C, 45 min) to remove [15]-crown-[5] and unreacted excess phenylglycinol.

Yield: 1.63 g, 4.3 mmol, 66 %.
^{1}H NMR (400 MHz, 298 K, $CDCl_3$): δ_H 7.36-7.20 (14H, m, Ph), 4.51 (4H, s, OC*H*$_2$Ph), 4.20 (2H, dd, $^{3}J_{HH}$ = 9.0 Hz, 3.8 Hz, CH), 3.57 (2H, dd, $^{2}J_{HH}$ = 9.0 Hz, $^{3}J_{HH}$ = 3.8 Hz, OC*H*$_2$CH), 3.41 (2H, t, $^{2}J_{HH}$/$^{3}J_{HH}$ = 9.0, OC*H*$_2$CH), 1.70 (4H, br s, NH_2).
^{13}C{^{1}H} NMR (100 MHz, 298 K, $CDCl_3$): δ_C 142.6 (Ph), 137.8 (Ph), 128.5 (Ph), 127.9 (Ph), 127.5 (Ph), 127.0 (Ph), 76.8 (O*C*H$_2$CH), 73.1 (O*C*H$_2$Ph), 55.7 (CH).
MS (ESI): m/z 377.2 $[M+H]^+$, 189.1 $[M+2H]^{2+}$.

FTIR: ν cm^{-1} 2873 m, 1602 w, 1515 w, 1471 m, 1452 w, 1420 w, 1354 m, 1308 w, 1212 w, 1088 s, 1020 m, 848 m, 759 s, 701 s.
Elemental analysis found (calculated for $C_{24}H_{28}N_2O_2$): % C 75.95 (76.56) H 7.34 (7.50) N 7.07 (7.44).

(*S*,*S*)-α,α'-*bis*(2-amino-2-phenylethoxy)-*p*-xylene [(*S*,*S*)-3a]

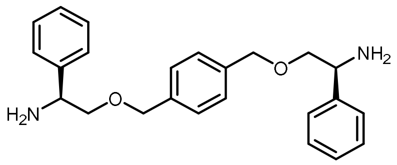

Synthesised according to the procedure described for (*R*,*R*)-**3a**; substituting (*R*)-2-phenylglycinol for (*S*)-2-phenylglycinol.

Yield: 1.71 g, 4.5 mmol, 69 %.
^{1}H NMR (400 MHz, 298 K, $CDCl_3$): δ_H 7.36-7.20 (14H, m, Ph), 4.51 (4H, s, OC*H*$_2$Ph), 4.20 (2H, dd, $^3J_{HH}$ = 9.0 Hz, 3.8 Hz, CH), 3.57 (2H, dd, $^2J_{HH}$ = 9.0 Hz, $^3J_{HH}$ = 3.8 Hz, OC*H*$_2$CH), 3.41 (2H, t, $^2J_{HH}$/$^3J_{HH}$ = 9.0, OC*H*$_2$CH), 1.70 (4H, br s, NH_2).
^{13}C{^{1}H} NMR (100 MHz, 298 K, $CDCl_3$): δ_C 142.6 (Ph), 137.8 (Ph), 128.5 (Ph), 127.9 (Ph), 127.5 (Ph), 127.0 (Ph), 76.8 (O*C*H$_2$CH), 73.1 (O*C*H$_2$Ph), 55.7 (CH).
MS (ESI): m/z 399.2 [M+Na]$^+$, 377.2 [M+H]$^+$, 189.1 [M+2H]$^{2+}$.
FTIR: ν cm^{-1} 2873 m, 1602 w, 1515 w, 1471 m, 1452 w, 1420 w, 1354 m, 1308 w, 1212 w, 1088 s, 1020 m, 848 m, 759 s, 701 s.
Elemental analysis found (calculated for $C_{24}H_{28}N_2O_2$): % C 75.95 (76.56) H 7.99 (7.50) N 6.94 (7.44).

(*R*,*R*)-α,α'-*bis*(2-amino-2-phenylethoxy)-*m*-xylene [(*R*,*R*)-3b]

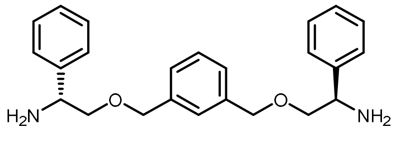

Under inert atmosphere, (*R*)-2-phenylglycinol (1.14 g, 8.3 mmol, 2.2 eq.) was dissolved in anhydrous THF (50 ml), to which 1.2 ml (1.33 g, 6.0 mmol, 1.6 eq.) [15]-crown-[5] was added by injection. The solution was then added dropwise to neat sodium hydride (0.42 g, 17.5 mmol, 4.6 eq.). The effervescent mixture was carefully placed under static vacuum and stirred at ambient temperature for 1 h. A solution of α,α'-dibromo-*m*-xylene (1.0 g, 3.8 mmol, 1 eq.) in anhydrous THF (40 ml) was then added dropwise. The reaction mixture was then stirred under static vacuum; for 1 h at ambient temperature, then 5 h at 65 °C. The pink reaction mixture was allowed to cool before quenching with 2:1 saturated KCl aq./water (60 ml). The crude product was extracted using diethyl ether (3 × 100 ml), dried over sodium sulfate, filtered through celite, and the solvent removed under reduced pressure to leave a yellow oil. This crude product was purified by Kügelrohr distillation (150 °C, 45 min) to remove [15]-crown-[5] and unreacted excess phenylglycinol.

Yield: 1.22 g, 3.2 mmol, 81 %.
^{1}H NMR (300 MHz, 298 K, $CDCl_3$): δ_H 7.40-7.23 (14H, m, Ph), 4.55 (4H, s, OCH_2Ph), 4.24 (2H, dd, $^{3}J_{HH}$ = 9.0 Hz, 3.8 Hz, CH), 3.61 (2H, dd, $^{2}J_{HH}$ = 9.0 Hz, $^{3}J_{HH}$ = 3.8 Hz, OCH_2CH), 3.46 (2H, t, $^{2}J_{HH}$/$^{3}J_{HH}$ = 9.0, OCH_2CH), 1.75 (4H, br s, NH_2).
^{13}C{^{1}H} NMR (75 MHz, 298 K, $CDCl_3$): δ_C 142.6 (Ph), 138.5 (Ph), 128.7 (Ph), 128.6 (Ph), 127.5 (Ph), 127.2 (Ph), 127.1 (Ph), 127.0 (Ph), 76.9 (O*C*H_2CH), 73.3 (O*C*H_2Ph), 55.7 (CH).
MS (ESI): m/z 399.2 [M+Na]$^{+}$, 377.2 [M+H]$^{+}$, 189.1 [M+2H]$^{2+}$.
FTIR: ν cm^{-1} 2853 m, 1602 w, 1492 m, 1452 m, 1254 m, 1155 m, 1084 s, 1027 m, 860 m, 791 w, 759 s, 701 s.
Elemental analysis found (calculated for $C_{24}H_{28}N_2O_2$): % C 75.74 (76.56) H 7.94 (7.50) N 7.30 (7.44).

(*S*,*S*)-α,α'-*bis*(2-amino-2-phenylethoxy)-*m*-xylene [(*S*,*S*)-3b]

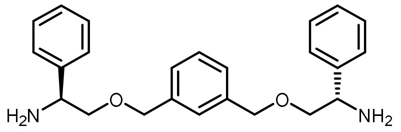

Synthesised according to the procedure described for (*R*,*R*)-**3b**; substituting (*R*)-2-phenylglycinol for (*S*)-2-phenylglycinol.

Yield: 1.16 g, 3.1 mmol, 85 %.
^{1}H NMR (300 MHz, 298 K, $CDCl_3$): δ_H 7.40-7.23 (14H, m, Ph), 4.55 (4H, s, OC*H*$_2$Ph), 4.24 (2H, dd, $^{3}J_{HH}$ = 9.0 Hz, 3.8 Hz, CH), 3.61 (2H, dd, $^{2}J_{HH}$ = 9.0 Hz, $^{3}J_{HH}$ = 3.8 Hz, OC*H*$_2$CH), 3.46 (2H, t, $^{2}J_{HH}$/$^{3}J_{HH}$ = 9.0, OC*H*$_2$CH), 1.75 (4H, br s, NH_2).
^{13}C{^{1}H} NMR (75 MHz, 298 K, $CDCl_3$): δ_C 142.6 (Ph), 138.5 (Ph), 128.7 (Ph), 128.6 (Ph), 127.5 (Ph), 127.2 (Ph), 127.1 (Ph), 127.0 (Ph), 76.9 (O*C*H$_2$CH), 73.3 (O*C*H$_2$Ph), 55.7 (CH).
MS (ESI): m/z 399.2 $[M+Na]^+$, 377.2 $[M+H]^+$, 189.1 $[M+2H]^{2+}$.
FTIR: ν cm^{-1} 2853 m, 1602 w, 1492 m, 1452 m, 1254 m, 1155 m, 1084 s, 1027 m, 860 m, 791 w, 759 s, 701 s.
Elemental analysis found (calculated for $C_{24}H_{28}N_2O_2$): % C 75.72 (76.56) H 7.61 (7.50) N 7.17 (7.44).

(*R*,*R*)-4,4'-*bis*[(2-amino-2-phenylethoxy)methyl]-1,1'-biphenyl [(*R*,*R*)-3c]

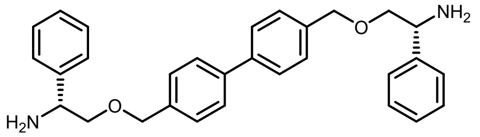

Under inert atmosphere, (*R*)-2-phenylglycinol (0.86 g, 6.3 mmol, 2.1 eq.) was dissolved in anhydrous THF (50 ml), to which 0.6 ml (0.67 g, 3.0 mmol, 1.0 eq.) [15]-crown-[5] was added by injection. The solution was then added dropwise to neat sodium hydride (0.31 g, 12.9 mmol, 4.3 eq.). The effervescent mixture was carefully placed under static vacuum and stirred at ambient temperature for 1 h. A solution of 4,4'-*bis*(chloromethyl)-1,1'-biphenyl (0.75 g, 3.0 mmol, 1 eq.) in anhydrous THF (40 ml) was then added dropwise. The reaction mixture was then stirred under static vacuum; for 1 h at ambient temperature, then 5 h at 65 °C. The yellow reaction mixture was allowed to cool before quenching with 2:1 saturated KCl aq./water (60 ml). The crude product was extracted using diethyl ether (3 × 100 ml), dried over sodium sulfate,

filtered through celite, and the solvent removed under reduced pressure to leave a yellow oil. This crude product was purified by Kügelrohr distillation (150 °C, 45 min) to remove [15]-crown-[5] and unreacted excess phenylglycinol. Upon standing, the product solidified to give a pale yellow wax.

Yield: 0.82 g, 1.8 mmol, 61 %.
^{1}H NMR (300 MHz, 298 K, $CDCl_3$): δ_H 7.49 (4H, d, $^3J_{HH}$ = 8.1 Hz, Ph), 7.34-7.16 (14H, m, Ph), 4.52 (4H, s, OC*H*$_2$Ph), 4.19 (2H, dd, $^2J_{HH}$ = 8.9 Hz, $^3J_{HH}$ = 3.8 Hz, CH), 3.57 (2H, dd, $^2J_{HH}$ = 8.9 Hz, $^3J_{HH}$ = 3.8 Hz, OC*H*$_2$CH), 3.41 (2H, t, $^2J_{HH}/^3J_{HH}$ = 9.0, OC*H*$_2$CH), 1.73 (4H, br s, NH_2).
^{13}C{^{1}H} NMR (75 MHz, 298 K, $CDCl_3$): δ_C 142.6 (Ph), 140.4 (Ph), 137.4 (Ph), 128.5 (Ph), 128.3 (Ph), 127.5 (Ph), 127.2 (Ph), 127.0 (Ph), 76.8 (O*C*H$_2$CH), 73.0 (O*C*H$_2$Ph), 55.7 (CH).
MS (ESI): m/z 475. 2 $[M+Na]^+$, 453.2 $[M+H]^+$, 227.1 $[M+2H]^{2+}$.
FTIR: ν cm^{-1} 3383 w, 2853 m, 1561 w, 1502 w, 1492 m, 1454 m, 1396 w, 1373 w, 1348 m, 1305 m, 1210 w, 1183 w, 1108 s, 1015 m, 1003 m, 936 w, 860 m, 801 s, 758 s, 700 s.
Elemental analysis found (calculated for $C_{30}H_{32}N_2O_2$): % C 79.27 (79.61) H 7.25 (7.13) N 5.93 (6.19).

(*S*,*S*)-4,4'-*bis*[(2-amino-2-phenylethoxy)methyl]-1,1'-biphenyl [(*S*,*S*)-3c]

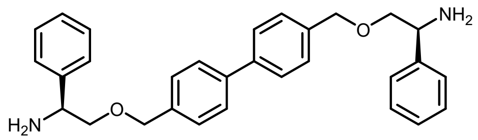

Synthesised according to the procedure described for (*R*,*R*)-**3c**; substituting (*R*)-2-phenylglycinol for (*S*)-2-phenylglycinol.

Yield: 1.05 g, 2.3 mmol, 78 %.
^{1}H NMR (300 MHz, 298 K, $CDCl_3$): δ_H 7.49 (4H, d, $^3J_{HH}$ = 8.1 Hz, Ph), 7.34-7.16 (14H, m, Ph), 4.52 (4H, s, OC*H*$_2$Ph), 4.19 (2H, dd, $^2J_{HH}$ = 8.9 Hz, $^3J_{HH}$ = 3.8 Hz, CH), 3.57 (2H, dd, $^2J_{HH}$ = 8.9 Hz, $^3J_{HH}$ = 3.8 Hz, OC*H*$_2$CH), 3.41 (2H, t, $^2J_{HH}/^3J_{HH}$ = 8.9, OC*H*$_2$CH), 1.73 (4H, br s, NH_2).

$^{13}C\{^1H\}$ NMR (75 MHz, 298 K, $CDCl_3$): δ_C 142.6 (Ph), 140.4 (Ph), 137.4 (Ph), 128.5 (Ph), 128.3 (Ph), 127.5 (Ph), 127.2 (Ph), 127.0 (Ph), 76.8 (O*C*H_2CH), 73.0 (O*C*H_2Ph), 55.7 (CH).
MS (ESI): m/z 475. 2 $[M+Na]^+$, 453.2 $[M+H]^+$, 227.1 $[M+2H]^{2+}$.
FTIR: ν cm^{-1} 3383 w, 2853 m, 1561 w, 1502 w, 1492 m, 1454 m, 1396 w, 1373 w, 1348 m, 1305 m, 1210 w, 1183 w, 1108 s, 1015 m, 1003 m, 936 w, 860 m, 801 s, 758 s, 700 s.
Elemental analysis found (calculated for $C_{30}H_{32}N_2O_2$): % C 79.27 (79.61) H 7.31 (7.13) N 5.92 (6.19).

(*R*,*R*)-4,4'-*bis*[(2-amino-2-phenylethoxy)methyl]-diphenyl ether [(*R*,*R*)-3d]

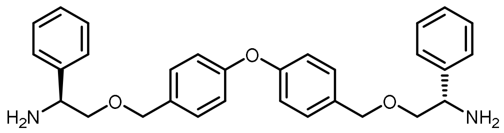

Under inert atmosphere, (*R*)-2-phenylglycinol (0.66 g, 4.8 mmol, 2.1 eq.) was dissolved in anhydrous THF (50 ml), to which 0.6 ml (0.67 g, 3.0 mmol, 1.3 eq.) [15]-crown-[5] was added by injection. The solution was then added dropwise to neat sodium hydride (0.25 g, 10.4 mmol, 4.6 eq.). The effervescent mixture was carefully placed under static vacuum and stirred at ambient temperature for 1 h. A solution of *bis*-4-(Bromomethyl)phenyl ether (see above for synthesis, 0.8 g, 2.3 mmol, 1 eq.) in anhydrous THF (40 ml) was then added dropwise. The reaction mixture was then stirred under static vacuum; for 1 h at ambient temperature, then 5 h at 65 °C. The brick red reaction mixture was allowed to cool before quenching with 2:1 saturated KCl aq./water (60 ml). The crude product was extracted using diethyl ether (3 × 100 ml), dried over sodium sulfate, filtered through celite, and the solvent removed under reduced pressure to leave a yellow oil. This crude product was purified by Kügelrohr distillation (150 °C, 45 min) to remove [15]-crown-[5] and unreacted excess phenylglycinol.

Yield: 0.78 g, 1.6 mmol, 72 %.
^1H NMR (300 MHz, 298 K, $CDCl_3$): δ_H 7.40-7.22 (14H, m, Ph), 6.96 (4H, d, $^3J_{HH}$ = 8.3 Hz, Ph), 4.51 (4H, s, OC*H*$_2$Ph), 4.23 (2H, dd, $^2J_{HH}$ = 8.9 Hz, $^3J_{HH}$ = 3.7 Hz, CH), 3.61 (2H, dd, $^2J_{HH}$ = 8.9 Hz, $^3J_{HH}$ = 3.7 Hz, OC*H*$_2$CH), 3.45 (2H, t, $^2J_{HH}/^3J_{HH}$ = 8.9, OC*H*$_2$CH), 1.74 (4H, br s, NH_2).

$^{13}C\{^1H\}$ NMR (75 MHz, 298 K, $CDCl_3$): δ_C 156.8 (Ph), 142.6 (Ph), 133.2 (Ph), 129.4 (Ph), 128.5 (Ph), 127.5 (Ph), 126.9 (Ph), 118.9 (Ph), 76.7 (O*C*H_2CH), 72.9 (O*C*H_2Ph), 55.7 (CH).
MS (ESI): m/z 491.2 $[M+Na]^+$, 469.2 $[M+H]^+$, 235.1 $[M+2H]^{2+}$.
FTIR: ν cm^{-1} 3027 w, 2855 w, 1601 m, 1500 s, 1452 w, 1354 w, 1236 s, 1166 w, 1087 s, 1014 w, 874 m, 853 m, 760 s, 701 s.
Elemental analysis found (calculated for $C_{30}H_{32}N_2O_3$): % C 76.47 (76.90) H 7.14 (6.88) N 5.65 (5.98).

(*S*,*S*)-4,4'-*bis*[(2-amino-2-phenylethoxy)methyl]-diphenyl ether [(*S*,*S*)-3d]

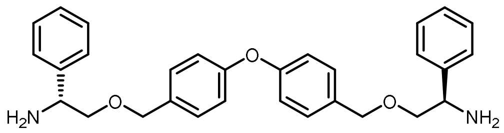

Synthesised according to the procedure described for (*R*,*R*)-**3d**; substituting (*R*)-2-phenylglycinol for (*S*)-2-phenylglycinol.

Yield: 0.77 g, 1.6 mmol, 73 %.
^1H NMR (300 MHz, 298 K, $CDCl_3$): δ_H 7.40-7.22 (14H, m, Ph), 6.96 (4H, d, $^3J_{HH}$ = 8.3 Hz, Ph), 4.51 (4H, s, OC*H_2*Ph), 4.23 (2H, dd, $^2J_{HH}$ = 8.9 Hz, $^3J_{HH}$ = 3.7 Hz, CH), 3.61 (2H, dd, $^2J_{HH}$ = 8.9 Hz, $^3J_{HH}$ = 3.7 Hz, OC*H_2*CH), 3.45 (2H, t, $^2J_{HH}/^3J_{HH}$ = 8.9, OC*H_2*CH), 1.74 (4H, br s, NH_2).
$^{13}C\{^1H\}$ NMR (75 MHz, 298 K, $CDCl_3$): δ_C 156.8 (Ph), 142.6 (Ph), 133.2 (Ph), 129.4 (Ph), 128.5 (Ph), 127.5 (Ph), 126.9 (Ph), 118.9 (Ph), 76.7 (O*C*H_2CH), 72.9 (O*C*H_2Ph), 55.7 (CH).
MS (ESI): m/z 491.2 $[M+Na]^+$, 469.2 $[M+H]^+$, 235.1 $[M+2H]^{2+}$.
FTIR: ν cm^{-1} 3027 w, 2855 w, 1601 m, 1500 s, 1452 w, 1354 w, 1236 s, 1166 w, 1087 s, 1014 w, 874 m, 853 m, 760 s, 701 s.

Elemental analysis found (calculated for $C_{30}H_{32}N_2O_3$): % C 76.48 (76.90) H 7.22 (6.88) N 5.65 (5.98).

(*R*,*R*)-4,4'-*bis*[(2-amino-2-phenylethoxy)methyl]-diphenyl sulfide [(*R*,*R*)-3e]

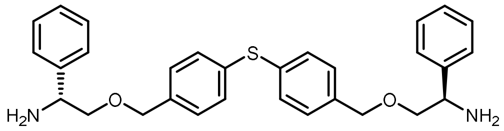

Under inert atmosphere, (*R*)-2-phenylglycinol (0.64 g, 4.7 mmol, 2.1 eq.) was dissolved in anhydrous THF (50 ml), to which 0.6 ml (0.67 g, 3.0 mmol, 1.4 eq.) [15]-crown-[5] was added by injection. The solution was then added dropwise to neat sodium hydride (0.24 g, 10.0 mmol, 4.7 eq.). The effervescent mixture was carefully placed under static vacuum and stirred at ambient temperature for 1 h. A solution of *bis*-4-(bromomethyl)phenyl sulfide (see above for synthesis, 0.8 g, 2.2 mmol, 1 eq.) in anhydrous THF (40 ml) was then added dropwise. The reaction mixture was then stirred under static vacuum; for 1 h at ambient temperature, then 5 h at 65 °C. The dark purple reaction mixture was allowed to cool before quenching with 2:1 saturated KCl aq./water (60 ml). The crude product was extracted using diethyl ether (3 × 100 ml), dried over sodium sulfate, filtered through celite, and the solvent removed under reduced pressure to leave a yellow oil. This crude product was purified by Kügelrohr distillation (150 °C, 45 min) to remove [15]-crown-[5] and unreacted excess phenylglycinol.

Yield: 0.88 g, 1.8 mmol, 84 %.
^{1}H NMR (300 MHz, 298 K, $CDCl_3$): δ_H 7.41-7.23 (18H, m, Ph), 4.53 (4H, s, OCH_2Ph), 4.25 (2H, dd, $^2J_{HH}$ = 8.8 Hz, $^3J_{HH}$ = 3.7 Hz, CH), 3.62 (2H, dd, $^2J_{HH}$ = 8.8 Hz, $^3J_{HH}$ = 3.7 Hz, OCH_2CH), 3.46 (2H, t, $^2J_{HH}$/$^3J_{HH}$ = 8.8, OCH_2CH), 1.76 (4H, br s, NH_2).
^{13}C{^{1}H} NMR (75 MHz, 298 K, $CDCl_3$): δ_C 142.6 (Ph), 137.4 (Ph), 135.1 (Ph), 131.2 (Ph), 128.6 (Ph), 128.5 (Ph), 127.5 (Ph), 126.9 (Ph), 76.9 (O*C*H_2CH), 72.9 (O*C*H_2Ph), 55.7 (CH).
MS (ESI): m/z 507.2 $[M+Na]^+$, 485.2 $[M+H]^+$, 243.1 $[M+2H]^{2+}$.
FTIR: ν cm^{-1} 2855 m, 1602 w, 1492 w, 1452 m, 1421 w, 1354 m, 1212 w, 1084 s, 1084 m, 848 m, 757 m, 700 s.
Elemental analysis found (calculated for $C_{30}H_{32}N_2O_2S$): % C 73.53 (74.35) H 6.83 (6.66) N 5.42 (5.78).

(*S*,*S*)-4,4'-*bis*[(2-amino-2-phenylethoxy)methyl]-diphenyl sulfide [(*S*,*S*)-3e]

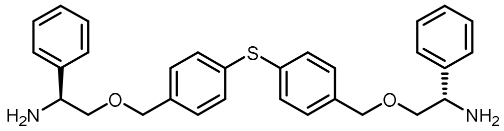

Synthesised according to the procedure described for (*R*,*R*)-**3e**; with the substitution of (*R*)-2-phenylglycinol for (*S*)-2-phenylglycinol.

Yield: 0.58 g, 1.2 mmol, 56 %.
^{1}H NMR (300 MHz, 298 K, $CDCl_3$): δ_H 7.41-7.23 (18H, m, Ph), 4.53 (4H, s, OC*H*$_2$Ph), 4.25 (2H, dd, $^2J_{HH}$ = 8.8 Hz, $^3J_{HH}$ = 3.7 Hz, CH), 3.62 (2H, dd, $^2J_{HH}$ = 8.8 Hz, $^3J_{HH}$ = 3.7 Hz, OC*H*$_2$CH), 3.46 (2H, t, $^2J_{HH}$/$^3J_{HH}$ = 8.8, OC*H*$_2$CH), 1.76 (4H, br s, NH_2).
$^{13}C\{^1H\}$ NMR (75 MHz, 298 K, $CDCl_3$): δ_C 142.6 (Ph), 137.4 (Ph), 135.1 (Ph), 131.2 (Ph), 128.6 (Ph), 128.5 (Ph), 127.5 (Ph), 126.9 (Ph), 76.9 (O*C*H$_2$CH), 72.9 (O*C*H$_2$Ph), 55.7 (CH).
MS (ESI): m/z 507.2 $[M+Na]^+$, 485.2 $[M+H]^+$, 243.1 $[M+2H]^{2+}$.
FTIR: ν cm^{-1} 2855 m, 1602 w, 1492 w, 1452 m, 1421 w, 1354 m, 1212 w, 1084 s, 1084 m, 848 m, 757 m, 700 s.
Elemental analysis found (calculated for $C_{30}H_{32}N_2O_2S$): % C 73.96 (74.35) H 6.89 (6.66) N 5.42 (5.78).

(*R*,*R*)-4,4'-*bis*[(2-amino-2-phenylethoxy)methyl]-diphenylmethane [(*R*,*R*)-3f]

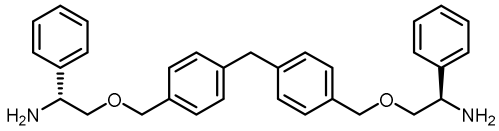

Under inert atmosphere, (*R*)-2-phenylglycinol (0.67 g, 4.9 mmol, 2.2 eq.) was dissolved in anhydrous THF (50 ml), to which 0.6 ml (0.67 g, 3.0 mmol, 1.3 eq.) [15]-crown-[5] was added by injection. The solution was then added dropwise to neat sodium hydride (0.24 g, 10.0 mmol, 4.4 eq.). The effervescent mixture was carefully placed under static vacuum and stirred at ambient temperature for 1 h. A solution of *bis*-4-(bromomethyl)phenylmethane (see above for

synthesis, 0.8 g, 2.3 mmol, 1 eq.) in anhydrous THF (40 ml) was then added dropwise. The reaction mixture was then stirred under static vacuum; for 1 h at ambient temperature, then 5 h at 65 °C. The orange-brown reaction mixture was allowed to cool before quenching with 2:1 saturated KCl aq./water (60 ml). The crude product was extracted using diethyl ether (3 × 100 ml), dried over sodium sulfate, filtered through celite, and the solvent removed under reduced pressure to leave a yellow oil. This crude product was purified by Kügelrohr distillation (150 °C, 45 min) to remove [15]-crown-[5] and unreacted excess phenylglycinol.

Yield: 0.74 g, 1.6 mmol, 70 %.

^{1}H NMR (300 MHz, 298 K, $CDCl_3$): δ_H 7.41-7.14 (18H, m, Ph), 4.52 (4H, s, OC*H*$_2$Ph), 4.24 (2H, dd, $^{2}J_{HH}$ = 8.9 Hz, $^{3}J_{HH}$ = 3.7 Hz, CH), 3.97 (2H, s, PhC*H*$_2$Ph), 3.61 (2H, dd, $^{2}J_{HH}$ = 8.9 Hz, $^{3}J_{HH}$ = 3.7 Hz, OC*H*$_2$CH), 3.45 (2H, t, $^{2}J_{HH}$/$^{3}J_{HH}$ = 8.9, OC*H*$_2$CH), 1.72 (4H, br s, NH_2).

^{13}C{^{1}H} NMR (75 MHz, 298 K, $CDCl_3$): δ_C 142.7 (Ph), 140.7 (Ph), 136.1 (Ph), 129.1 (Ph), 128.5 (Ph), 128.1 (Ph), 127.5 (Ph), 127.0 (Ph), 76.9 (O*C*H$_2$CH), 73.3 (O*C*H$_2$Ph), 55.7 (CH), 41.5 (Ph*C*H$_2$Ph).

MS (ESI): m/z 489.1 $[M+Na]^+$, 467.1 $[M+H]^+$.

FTIR: ν cm^{-1} 3057 w, 2896 w, 2853 m, 1603 w, 1511 w, 1492 w, 1452 m, 1418 w, 1354 w, 1182 w, 1088 s, 1020 m, 849 m, 804 w, 758 s, 701 s.

Elemental analysis found (calculated for $C_{31}H_{34}N_2O_2$): % C 80.20 (79.80) H 7.22 (7.34) N 5.23 (6.00).

(*S*,*S*)-4,4'-*bis*[(2-amino-2-phenylethoxy)methyl]-diphenylmethane [(*S*,*S*)-3f]

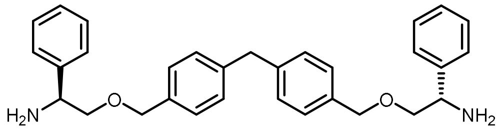

Synthesised according to the procedure described for (*R*,*R*)-**3f**; with the substitution of (*R*)-2-phenylglycinol for (*S*)-2-phenylglycinol.

Yield: 0.75 g, 1.6 mmol, 71 %.

^{1}H NMR (300 MHz, 298 K, $CDCl_3$): δ_H 7.41-7.14 (18H, m, Ph), 4.52 (4H, s, OCH_2Ph), 4.24 (2H, dd, $^2J_{HH}$ = 8.9 Hz, $^3J_{HH}$ = 3.7 Hz, CH), 3.97 (2H, s, PhCH_2Ph), 3.61 (2H, dd, $^2J_{HH}$ = 8.9 Hz, $^3J_{HH}$ = 3.7 Hz, OCH_2CH), 3.45 (2H, t, $^2J_{HH}$/$^3J_{HH}$ = 8.9, OCH_2CH), 1.72 (4H, br s, NH_2).
^{13}C{^{1}H} NMR (75 MHz, 298 K, $CDCl_3$): δ_C 142.7 (Ph), 140.7 (Ph), 136.1 (Ph), 129.1 (Ph), 128.5 (Ph), 128.1 (Ph), 127.5 (Ph), 127.0 (Ph), 76.9 (O*C*H_2CH), 73.3 (O*C*H_2Ph), 55.7 (CH), 41.5 (Ph*C*H_2Ph).
MS (ESI): m/z 489.1 $[M+Na]^+$, 467.1 $[M+H]^+$.
FTIR: ν cm^{-1} 3057 w, 2896 w, 2853 m, 1603 w, 1511 w, 1492 w, 1452 m, 1418 w, 1354 w, 1182 w, 1088 s, 1020 m, 849 m, 804 w, 758 s, 701 s.
Elemental analysis found (calculated for $C_{31}H_{34}N_2O_2$): % C 80.34 (79.80) H 7.55 (7.34) N 5.27 (6.00).

(*R*,*R*)-4,4'-bis[(2-amino-2-phenylethoxy)methyl]-1,2-diphenylethane [(*R*,*R*)-3g]

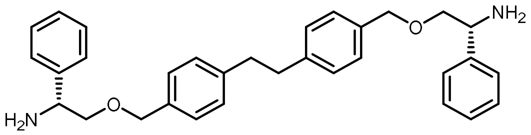

Under inert atmosphere, (*R*)-2-phenylglycinol (0.66 g, 4.8 mmol, 2.2 eq.) was dissolved in anhydrous THF (50 ml), to which 0.6 ml (0.67 g, 3.0 mmol, 1.4 eq.) [15]-crown-[5] was added by injection. The solution was then added dropwise to neat sodium hydride (0.24 g, 10.0 mmol, 4.6 eq.). The effervescent mixture was carefully placed under static vacuum and stirred at ambient temperature for 1 h. A solution of 1,2-*bis*-4-(bromomethyl)phenylethane (see above for synthesis, 0.8 g, 2.2 mmol, 1 eq.) in anhydrous THF (40 ml) was then added dropwise. The reaction mixture was then stirred under static vacuum; for 1 h at ambient temperature, then 5 h at 65 °C. The grey-brown reaction mixture was allowed to cool before quenching with 2:1 saturated KCl aq./water (60 ml). The crude product was extracted using diethyl ether (3 × 100 ml), dried over sodium sulfate, filtered through celite, and the solvent removed under reduced pressure to leave a yellow oil. This crude product was purified by Kügelrohr distillation (150 °C, 45 min) to

remove [15]-crown-[5] and unreacted excess phenylglycinol. Upon standing, the product solidified to give a yellow wax.

Yield: 0.8 g, 1.7 mmol, 77 %.

^{1}H NMR (300 MHz, 298 K, $CDCl_3$): δ_H 7.41-7.14 (18H, m, Ph), 4.53 (4H, s, OC*H*$_2$Ph), 4.24 (2H, dd, $^2J_{HH}$ = 8.9 Hz, $^3J_{HH}$ = 3.7 Hz, CH), 3.61 (2H, dd, $^2J_{HH}$ = 8.9 Hz, $^3J_{HH}$ = 3.7 Hz, OC*H*$_2$CH), 3.45 (2H, t, $^2J_{HH}/^3J_{HH}$ = 8.9, OC*H*$_2$CH), 2.91 (4H, s, CH_2CH_2), 1.73 (4H, br s, NH_2).

^{13}C{^{1}H} NMR (75 MHz, 298 K, $CDCl_3$): δ_C 142.7 (Ph), 141.4 (Ph), 135.9 (Ph), 128.6 (Ph), 128.5 (Ph), 128.0 (Ph), 127.5 (Ph), 127.0 (Ph), 76.8 (O*C*H$_2$CH), 73.3 (O*C*H$_2$Ph), 55.7 (CH), 37.7 (CH_2CH_2).

MS (ESI): m/z 481.2 $[M+H]^+$.

FTIR: ν cm^{-1} 3384 w, 3058 w, 3027 w, 2854 m, 1601 m, 1514 w, 1491 w, 1451 m, 1421 w, 1352 w, 1308 w, 1291 w, 1114 s, 1091 s, 1020 m, 820 s, 753 s, 700 s.

Elemental analysis found (calculated for $C_{32}H_{36}N_2O_2$): % C 79.71 (79.97) H 7.69 (7.55) N 5.69 (5.83).

(*S*,*S*)-4,4'-bis[(2-amino-2-phenylethoxy)methyl]-1,2-diphenylethane [(*S*,*S*)-3g]

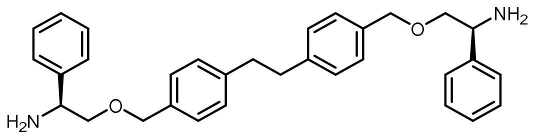

Synthesised according to the procedure described for (*R*,*R*)-**3g**; with the substitution of (*R*)-2-phenylglycinol for (*S*)-2-phenylglycinol.

Yield: 0.84 g, 1.7 mmol, 80 %.

^{1}H NMR (300 MHz, 298 K, $CDCl_3$): δ_H 7.41-7.14 (18H, m, Ph), 4.53 (4H, s, OC*H*$_2$Ph), 4.24 (2H, dd, $^2J_{HH}$ = 8.9 Hz, $^3J_{HH}$ = 3.7 Hz, CH), 3.61 (2H, dd, $^2J_{HH}$ = 8.9 Hz, $^3J_{HH}$ = 3.7 Hz, OC*H*$_2$CH), 3.45 (2H, t, $^2J_{HH}/^3J_{HH}$ = 8.9, OC*H*$_2$CH), 2.91 (4H, s, CH_2CH_2), 1.73 (4H, br s, NH_2).

$^{13}C\{^{1}H\}$ NMR (75 MHz, 298 K, $CDCl_3$): δ_C 142.7 (Ph), 141.4 (Ph), 135.9 (Ph), 128.6 (Ph), 128.5 (Ph), 128.0 (Ph), 127.5 (Ph), 127.0 (Ph), 76.8 (O*C*H_2CH), 73.3 (O*C*H_2Ph), 55.7 (CH), 37.7 (CH_2CH_2).
MS (ESI): m/z 481.2 $[M+H]^+$.
FTIR: ν cm^{-1} 3384 w, 3058 w, 3027 w, 2854 m, 1601 m, 1514 w, 1491 w, 1451 m, 1421 w, 1352 w, 1308 w, 1291 w, 1114 s, 1091 s, 1020 m, 820 s, 753 s, 700 s.
Elemental analysis found (calculated for $C_{32}H_{36}N_2O_2$): % C 80.05 (79.97) H 7.66 (7.55) N 5.69 (5.83).

(*R*,*R*)-2,8-*bis*[(2-amino-2-phenylethoxy)methyl]-dibenzofuran [(*R*,*R*)-3h]

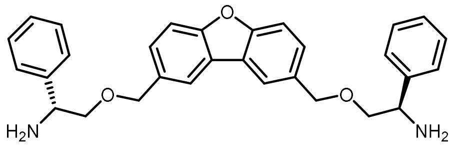

Under inert atmosphere, (*R*)-2-phenylglycinol (0.67 g, 6.0 mmol, 2.2 eq.) was dissolved in anhydrous THF (50 ml), to which 0.6 ml (0.67 g, 3.0 mmol, 1.3 eq.) [15]-crown-[5] was added by injection. The solution was then added dropwise to neat sodium hydride (0.4 g, 10.0 mmol, 4.4 eq.). The effervescent mixture was carefully placed under static vacuum and stirred at ambient temperature for 1 h. A solution of 2,8-*bis*(bromomethyl)dibenzofuran (see above for synthesis, 0.8 g, 2.3 mmol, 1 eq.) in anhydrous THF (40 ml) was then added dropwise. The reaction mixture was then stirred under static vacuum; for 1 h at ambient temperature, then 5 h at 65 °C. The dark green-grey reaction mixture was allowed to cool before quenching with 2:1 saturated KCl aq./water (60 ml). The crude product was extracted using diethyl ether (3 × 100 ml), dried over sodium sulfate, filtered through celite, and the solvent removed under reduced pressure to leave a yellow oil. This crude product was purified by Kügelrohr distillation (150 °C, 45 min) to remove [15]-crown-[5] and unreacted excess phenylglycinol.

Yield: 0.78 g, 1.7 mmol, 74 %.

^{1}H NMR (300 MHz, 298 K, $CDCl_3$): δ_H 7.81 (2H, s, Ph), 7.45 (2H, d, $^{3}J_{HH}$ = 8.5 Hz, Ph), 7.36-7.17 (12H, m, Ph), 4.62 (4H, s, OC*H*$_2$Ph), 4.20 (2H, dd, $^{2}J_{HH}$ = 8.9 Hz, $^{3}J_{HH}$ = 3.7 Hz, CH), 3.59 (2H, dd, $^{2}J_{HH}$ = 8.9 Hz, $^{3}J_{HH}$ = 3.7 Hz, OC*H*$_2$CH), 3.44 (2H, t, $^{2}J_{HH}$/$^{3}J_{HH}$ = 8.9, OC*H*$_2$CH), 1.74 (4H, br s, NH_2).
^{13}C{^{1}H} NMR (75 MHz, 298 K, $CDCl_3$): δ_C 156.2 (Ph), 142.6 (Ph), 132.9 (Ph), 128.5 (Ph), 127.5 (Ph), 127.3 (Ph), 126.9 (Ph), 124.3 (Ph), 120.3 (Ph), 111.6 (Ph), 76.7 (O*C*H$_2$CH), 73.5 (O*C*H$_2$Ph), 55.7 (CH).
MS (ESI): m/z 489.2 $[M+Na]^+$, 467.2 $[M+H]^+$, 234.1 $[M+2H]^{2+}$.
FTIR: ν cm^{-1} 3057 w, 3027 w, 2854 m, 1603 w, 1488 m, 1452 m, 1420 w, 1355 w, 1248 w, 1208 m, 1189 m, 1083 s, 1027 m, 876 m, 850 m, 808 m, 759 m, 700 s.
Elemental analysis found (calculated for $C_{30}H_{30}N_2O_3$): % C 76.36 (77.23) H 6.83 (6.48) N 5.83 (6.00).

(*S*,*S*)-2,8-*bis*[(2-amino-2-phenylethoxy)methyl]-dibenzofuran [(*S*,*S*)-3h]

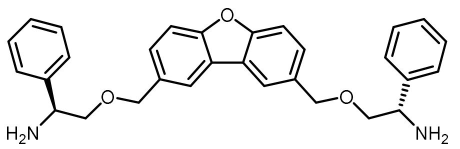

Synthesised according to the procedure described for (*R*,*R*)-**3h**; with the substitution of (*R*)-2-phenylglycinol for (*S*)-2-phenylglycinol.

Yield: 0.82 g, 1.8 mmol, 78 %.
^{1}H NMR (300 MHz, 298 K, $CDCl_3$): δ_H 7.81 (2H, s, Ph), 7.45 (2H, d, $^{3}J_{HH}$ = 8.5 Hz, Ph), 7.36-7.17 (12H, m, Ph), 4.62 (4H, s, OC*H*$_2$Ph), 4.20 (2H, dd, $^{2}J_{HH}$ = 8.9 Hz, $^{3}J_{HH}$ = 3.7 Hz, CH), 3.59 (2H, dd, $^{2}J_{HH}$ = 8.9 Hz, $^{3}J_{HH}$ = 3.7 Hz, OC*H*$_2$CH), 3.44 (2H, t, $^{2}J_{HH}$/$^{3}J_{HH}$ = 8.9, OC*H*$_2$CH), 1.74 (4H, br s, NH_2).

^{13}C{^{1}H} NMR (75 MHz, 298 K, $CDCl_3$): δ_C 156.2 (Ph), 142.6 (Ph), 132.9 (Ph), 128.5 (Ph), 127.5 (Ph), 127.3 (Ph), 126.9 (Ph), 124.3 (Ph), 120.3 (Ph), 111.6 (Ph), 76.7 (O*C*H$_2$CH), 73.5 (O*C*H$_2$Ph), 55.7 (CH).

MS (ESI): m/z 489.2 $[M+Na]^+$, 467.2 $[M+H]^+$, 234.1 $[M+2H]^{2+}$.

FTIR: ν cm^{-1} 3057 w, 3027 w, 2854 m, 1603 w, 1488 m, 1452 m, 1420 w, 1355 w, 1248 w, 1208 m, 1189 m, 1083 s, 1027 m, 876 m, 850 m, 808 m, 759 m, 700 s.

Elemental analysis found (calculated for $C_{30}H_{30}N_2O_3$): % C 76.36 (77.23) H 6.83 (6.48) N 5.83 (6.00).

Synthesis and characterisation of $[Zn_2L_3][ClO_4]_4$ complexes - General Procedure

The appropriate optically pure diamine (3.0 eq.) and 2-pyridinecarboxaldehyde (6.0 eq.) were dissolved in acetonitrile (30 ml) and stirred for 30 min at ambient temperature to form a yellow solution containing the ligand. Zinc(II) perchlorate hexahydrate (2.0 eq.) was added was added and no colour change was observed as the solution was stirred at ambient temperature for 4 h. The volume of the solution was reduced to ~10 ml under reduced pressure, and ethyl acetate was added dropwise to cause precipitation of a white solid, which was collected by filtration, washed with ethyl acetate (20 ml), and dried in air.

Δ_{Zn}-$[Zn_2L^b{}_3][ClO_4]_4$·$3H_2O$ (Δ-6b)

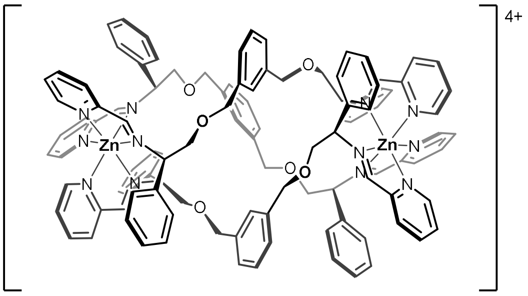

Δ_{Zn}-$[Zn_2\mathbf{L^b}_3]^{4+}$

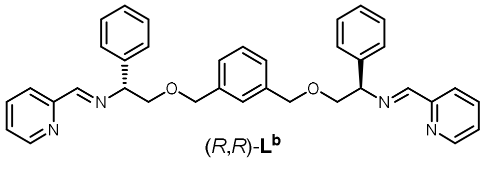

Yield: 64 %.

^{1}H NMR (500 MHz, 298 K, CD_3CN): δ_H 8 .65 (6H, s, HC=N), 8.30 (3H, s, Ph), 7.82 (6H, t, $^3J_{HH}$ = 7.8 Hz, Py), 7.65-7.60 (12H, m, Ph/Py), 7.53 (3H, t, $^3J_{HH}$ = 7.5, Ph), 7.34 (6H, dd , $^3J_{HH}$ = 7.5 Hz, 5.3 Hz, Py), 7.25 (6H, d, $^3J_{HH}$ = 7.8 Hz, Py), 6.95 (6H, t, $^3J_{HH}$ = 7.6 Hz, Ph), 6.75 (12H, t, $^3J_{HH}$ = 7.6 Hz, Ph), 6.36 (12H, d, $^3J_{HH}$ = 7.6 Hz, Ph), 5.63 (6H, dd, $^3J_{HH}$ = 11.1 Hz, 2.6 Hz, CH),

5.01 (6H, d, $^{2}J_{HH}$ = 10.7 Hz, OCH_2Ph), 4.86 (6H, d, $^{2}J_{HH}$ = 10.7 Hz, OCH_2Ph), 4.11 (6H, t, $^{2}J_{HH}$ /$^{3}J_{HH}$ = 11.4 Hz, OCH_2CH), 3.17 (6H, dd, $^{2}J_{HH}$ = 11.4 Hz, $^{3}J_{HH}$ = 3.0 Hz, OCH_2CH).

^{13}C{^{1}H} NMR (125 MHz, 298 K, CD_3CN): δ_C 163.3 (C=N), 148.9 (Ar), 147.1 (Ar), 142.7 (Ar), 139.3 (Ar), 135.6 (Ar), 130.5 (Ar), 130.1 (Ar), 130.0 (Ar), 129.6 (Ar), 129.5 (Ar), 129.0 (Ar), 128.1 (Ar), 127.2 (Ar), 75.2 (O*C*H_2Ph), 73.0 (O*C*H_2CH), 67.8 (CH).

MS (ESI): m/z 448.67 [Zn_2**L**$^{b}{}_3$]$^{4+}$, 309.10 [Zn_2**L**^{b}]$^{2+}$.

Elemental analysis found (calculated for $C_{108}H_{102}Cl_4N_{12}O_{22}Zn_2.3H_2O$): % C 57.49 (57.74) H 4.46 (4.85) N 7.62 (7.48).

Δ_{Zn}-[$Zn_2L^{c}{}_3$][ClO_4]$_4$·10H_2O (Δ-6c)

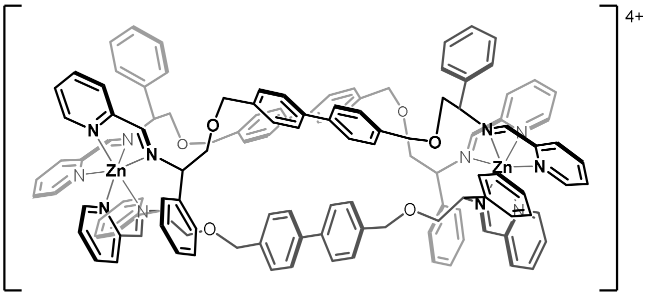

Δ_{Zn}-[Zn_2**L**$^{c}{}_3$]$^{4+}$

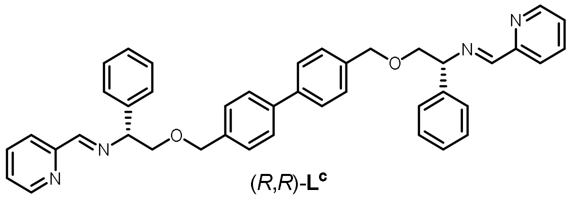

(*R*,*R*)-**L**^{c}

Yield: 53 %.

^{1}H NMR (500 MHz, 298 K, CD_3CN): δ_H 8.76 (6H, s, HC=N), 7.93 (6H, t, $^{3}J_{HH}$ = 7.8 Hz, Py), 7.72 (6H, d, $^{3}J_{HH}$ = 4.9 Hz, Py), 7.45 (6H, dd , $^{3}J_{HH}$ = 7.6 Hz, 5.5 Hz, Py), 7.40-7.35 (18H, m, Ph/Py), 7.25 (12H, d, $^{3}J_{HH}$ = 8.1 Hz, Ph), 7.08 (6H, t, $^{3}J_{HH}$ = 7.4 Hz, Ph), 6.96 (12H, t, $^{3}J_{HH}$ = 7.6 Hz, Ph), 6.74 (12H, d, $^{3}J_{HH}$ = 7.6 Hz, Ph), 5.75 (6H, dd, $^{3}J_{HH}$ = 10.3 Hz, 2.9 Hz, CH), 5.11 (6H, d, $^{2}J_{HH}$ = 13.5 Hz, OCH_2Ph), 4.61 (6H, d, $^{2}J_{HH}$ = 13.5 Hz, OCH_2Ph), 4.09 (6H, t, $^{2}J_{HH}$ /$^{3}J_{HH}$ = 10.9 Hz, OCH_2CH), 3.81 (6H, dd, $^{2}J_{HH}$ = 10.9 Hz, $^{3}J_{HH}$ = 3.2 Hz, OCH_2CH).

^{13}C{^{1}H} NMR (125 MHz, 298 K, CD_3CN): δ_C 164.1 (C=N), 149.1 (Ar), 147.3 (Ar), 142.8 (Ar), 140.7 (Ar), 137.9 (Ar), 136.2 (Ar), 130.6 (Ar), 129.8 (Ar), 129.15 (Ar(, 129.1 (Ar), 128.1 (Ar), 127.4 (Ar), 72.9 (O*C*H_2Ph), 71.8 (O*C*H_2CH), 68.2 (CH).

MS (ESI): m/z 631.3 [**L**^{d}+H]$^{+}$.

Elemental analysis found (calculated for $C_{126}H_{114}Cl_4N_{12}O_{22}Zn_2 \cdot 10H_2O$): % C 57.79 (58.18) H 4.44 (5.19) N 6.15 (6.46).

Λ_{Zn}-[$Zn_2L^d{}_3$][ClO_4]$_4$·8H_2O (Λ-6d)

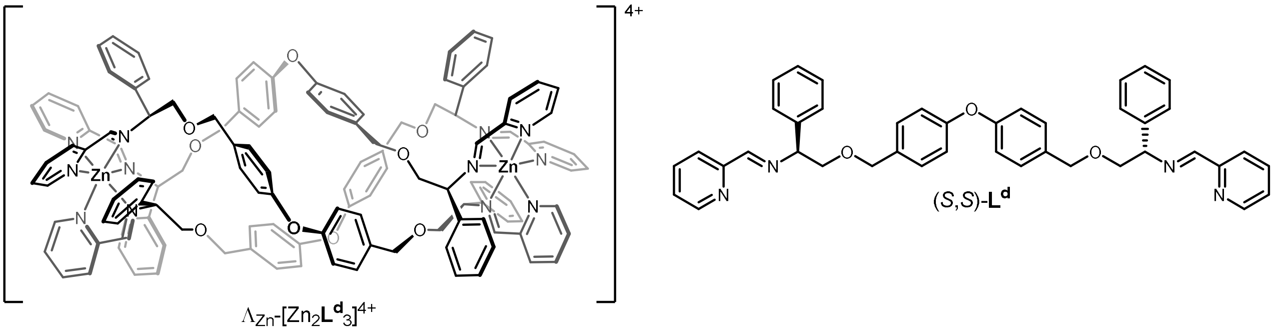

Yield: 78 %.

^{1}H NMR (500 MHz, 298 K, CD_3CN): δ_H 8.74 (6H, s, HC=N), 7.85 (6H, t, $^3J_{HH}$ = 7.8 Hz, Py), 7.65 (6H, d, $^3J_{HH}$ = 4.9 Hz, Py), 7.45 (12H, d, $^3J_{HH}$ = 8.5 Hz, Ph), 7.36 (6H, dd , $^3J_{HH}$ = 7.0 Hz, 5.2 Hz, Py), 7.31 (6H, d, $^3J_{HH}$ = 7.7 Hz, Py), 7.02 (6H, t, $^3J_{HH}$ = 7.4 Hz, Ph), 6.88 (12H, t, $^3J_{HH}$ = 7.6 Hz, Ph), 6.73 (12H, d, $^3J_{HH}$ = 8.5 Hz, Ph), 6.60 (12H, d, $^3J_{HH}$ = 7.6 Hz, Ph), 5.75 (6H, dd, $^3J_{HH}$ = 5.7 Hz, $^3J_{HH}$ = 2.0 Hz, CH), 4.95 (6H, d, $^2J_{HH}$ = 12.2 Hz, OCH_2Ph), 4.42 (6H, d, $^2J_{HH}$ = 12.2 Hz, OCH_2Ph), 4.19 (6H, t, $^2J_{HH}$ /$^3J_{HH}$ = 11.0 Hz, OCH_2CH), 3.36 (6H, dd, $^2J_{HH}$ = 11.0 Hz, $^3J_{HH}$ = 2.6 Hz, OCH_2CH).

^{13}C{^{1}H} NMR (125 MHz, 298 K, CD_3CN): δ_C 163.4 (C=N), 157.6 (Ar), 148.9 (Ar), 147.2 (Ar), 142.7 (Ar), 136.0 (Ar), 134.3 (Ar), 130.6 (Ar), 129.8 (Ar), 129.7 (Ar), 129.6 (Ar), 129.1 (Ar), 127.2 (Ar), 119.5 (Ar), 73.1 (O*C*H2Ph), 72.3 (O*C*H2CH), 68.3 (CH).

MS (ESI): m/z 669.3 [$\mathbf{L}^e$+Na]$^+$, 647.3 [$\mathbf{L}^e$+H]$^+$.

Elemental analysis found (calculated for $C_{126}H_{114}Cl_4N_{12}O_{25}Zn_2.8H_2O$): % C 57.87 (57.98) H 4.25 (4.90) N 6.33 (6.44).

Λ_{Zn}-[$Zn_2L^e{}_3$][ClO_4]$_4$.5H_2O (Λ-6e)

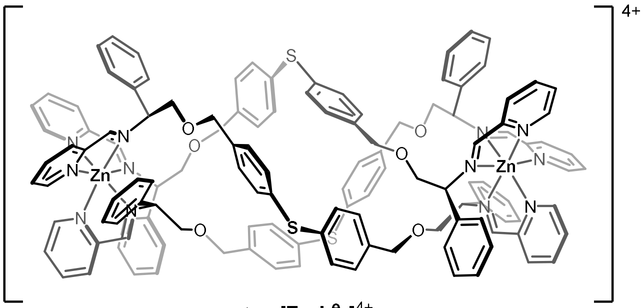

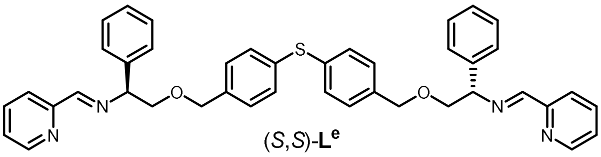

(S,S)-$\mathbf{L}^e$

Λ_{Zn}-$[Zn_2\mathbf{L}^e{}_3]^{4+}$

Yield: 76 %.

^{1}H NMR (500 MHz, 298 K, CD_3CN): δ_H 8.72 (6H, s, HC=N), 7.83 (6H, t, $^3J_{HH}$ = 7.8 Hz, Py), 7.60 (6H, d, $^3J_{HH}$ = 4.8 Hz, Py), 7.45 (12H, d, $^3J_{HH}$ = 8.2 Hz, Ph), 7.33 (6H, dd , $^3J_{HH}$ = 7.5 Hz, 5.6 Hz, Py), 7.28 (6H, d, $^3J_{HH}$ = 8.0 Hz, Py), 7.07 (12H, d, $^3J_{HH}$ = 8.2 Hz, Ph), 7.01 (6H, t, $^3J_{HH}$ = 7.4 Hz, Ph), 6.88 (12H, t, $^3J_{HH}$ = 7.6 Hz, Ph), 6.56 (12H, d, $^3J_{HH}$ = 7.6 Hz, Ph), 5.66 (6H, d, $^3J_{HH}$ = 10.4 Hz, CH), 4.92 (6H, d, $^2J_{HH}$ = 12.1 Hz, OCH_2Ph), 4.39 (6H, d, $^2J_{HH}$ = 12.1 Hz, OCH_2Ph), 4.20 (6H, t, $^2J_{HH}$ /$^3J_{HH}$ = 11.0 Hz, OCH_2CH), 3.13 (6H, dd, $^2J_{HH}$ = 11.0 Hz, $^3J_{HH}$ = 2.5 Hz, OCH_2CH).

$^{13}C\{^1H\}$ NMR (125 MHz, 298 K, CD_3CN): δ_C 163.1 (C=N), 148.8 (Ar), 145.2 (Ar), 142.7 (Ar), 138.1 (Ar), 135.9 (Ar), 135.6 (Ar), 131.0 (Ar), 130.6 (Ar), 129.8 (Ar), 129.5 (Ar), 129.2 (Ar), 127.1 (Ar), 73.2 (O*C*H_2Ph), 72.7 (O*C*H_2CH), 68.2 (CH).

MS (ESI): m/z 739.0 $[Zn_2\mathbf{L}^f{}_3][ClO_4]^{3+}$, 684.9 $[\mathbf{L}^f+Na]_+$, 529.4 $[Zn_2\mathbf{L}^f{}_3]^{4+}$.

Elemental analysis found (calculated for $C_{126}H_{114}Cl_4N_{12}O_{22}S_3Zn_2.5H_2O$): C 57.71 (57.98) H 4.36 (4.90) N 6.23 (6.44).

Λ_{Zn}-$[Zn_2L^f{}_3][ClO_4]_4\cdot 2H_2O\cdot 2EtOAc$ (Λ-6f)

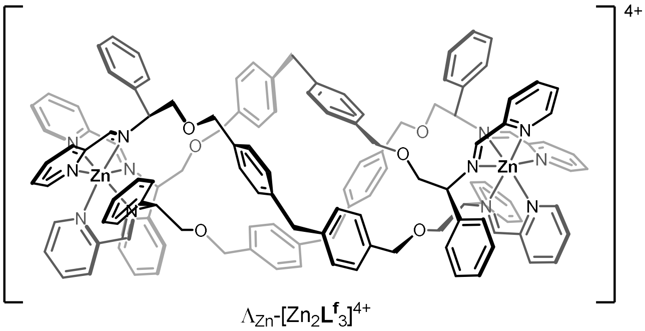

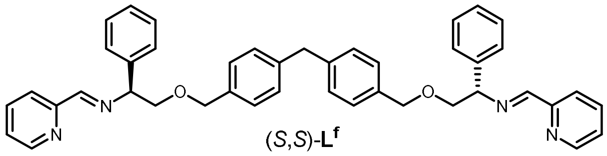

(S,S)-$\mathbf{L}^f$

Λ_{Zn}-$[Zn_2\mathbf{L}^f{}_3]^{4+}$

Yield: 68 %.

^{1}H NMR (500 MHz, 298 K, CD_3CN): δ_H 8.71 (6H, s, HC=N), 7.85 (6H, t, $^3J_{HH}$ = 7.8 Hz, Py), 7.64 (6H, d, $^3J_{HH}$ = 4.8 Hz, Py), 7.37-7.31 (24H, m, Ph/Py), 7.02 (6H, t, $^3J_{HH}$ = 7.4 Hz, Ph), 6.91 (12H, d, $^3J_{HH}$ = 8.0 Hz, Ph), 6.88 (12H, t, $^3J_{HH}$ = 7.7 Hz, Ph), 6.58 (12H, d, $^3J_{HH}$ = 7.6 Hz, Ph), 5.69 (6H, dd, $^3J_{HH}$ = 10.3 Hz, $^3J_{HH}$ = 2.2 Hz, CH), 4.83 (6H, d, $^2J_{HH}$ = 12.0 Hz, OCH_2Ph), 4.37 (6H, d, $^2J_{HH}$ = 12.0 Hz, OCH_2Ph), 4.13 (6H, t, $^2J_{HH}$ /$^3J_{HH}$ = 10.9 Hz, OCH_2CH), 3.83 (6H, s, PhCH_2Ph), 3.32 (6H, dd, $^2J_{HH}$ = 10.9 Hz, $^3J_{HH}$ = 2.9 Hz, OCH_2CH).

^{13}C{^{1}H} NMR (125 MHz, 298 K, CD_3CN): δ_C 163.5 (C=N), 148.9 (Ar), 147.2 (Ar), 142.7 (Ar), 141.4 (Ar), 136.6 (Ar), 136.1 (Ar), 130.5 (Ar), 129.8 (Ar), 129.7 (Ar), 129.6 (Ar), 129.1 (Ar), 128.4 (Ar), 127.3 (Ar), 73.6 (O*C*H_2Ph), 72.5 (O*C*H_2CH), 68.1 (CH), 41.0 (Ph*C*H_2Ph).

MS (ESI): m/z 667.3 [**L**^{g}+Na]$^+$, 645.3 [**L**^{g}+H]$^+$.

Elemental analysis found (calculated for $C_{129}H_{120}Cl_4N_{12}O_{22}Zn_2 \cdot 2H_2O \cdot 2EtOAc$): % C 61.47 (61.51) H 4.75 (5.28) N 6.03 (6.28).

Λ_{Zn}-[$Zn_2L^g_3$][ClO_4]$_4$ (Λ-6g)

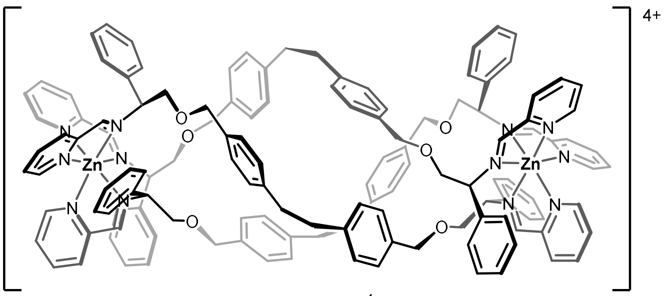

Λ_{Zn}-[Zn_2**L**$^g_3$]$^{4+}$

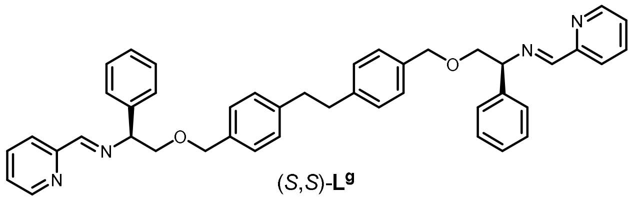

(*S*,*S*)-**L**^{g}

^{1}H NMR (500 MHz, 298 K, CD_3CN): δ_H 8.67 (6H, s, HC=N), 7.89 (6H, t, $^3J_{HH}$ = 7.7 Hz, Py), 7.65 (6H, d, $^3J_{HH}$ = 4.9 Hz, Py), 7.42-7.33 (24H, m, Ph/Py), 7.12 (12H, d, $^3J_{HH}$ = 7.9 Hz, Ph), 7.04 (6H, t, $^3J_{HH}$ = 7.4 Hz, Ph), 6.89 (12H, t, $^3J_{HH}$ = 7.7 Hz, Ph), 6.63 (12H, d, $^3J_{HH}$ = 7.6 Hz, Ph), 5.63 (6H, dd, $^3J_{HH}$ = 10.3 Hz, $^3J_{HH}$ = 2.7 Hz, CH), 4.86 (6H, d, $^2J_{HH}$ = 12.2 Hz, OCH_2Ph), 4.50 (6H, d, $^2J_{HH}$ = 12.2 Hz, OCH_2Ph), 4.02 (6H, t, $^2J_{HH}$ /$^3J_{HH}$ = 10.7 Hz, OCH_2CH), 3.59 (6H, dd, $^2J_{HH}$ = 11.2 Hz, $^3J_{HH}$ = 3.2 Hz, OCH_2CH), 2.79 (12H, s, CH_2CH_2).

^{13}C{^{1}H} NMR (125 MHz, 298 K, CD_3CN): δ_C 163.8 (C=N), 149.0 (Ar), 147.2 (Ar), 142.7 (Ar), 143.6 (Ar), 136.4 (Ar), 136.2 (Ar), 130.6 (Ar), 129.7 (Ar), 129.6 (Ar), 129.2 (Ar), 129.1 (Ar), 127.4 (Ar), 73.8 (O*C*H_2Ph), 72.1 (O*C*H_2CH), 68.1 (CH), 37.9 (CH_2CH_2).

MS (ESI): m/z 681.3 [**L**^{h}+Na]$^+$, 659.3 [**L**^{h}+H]$^+$.

Δ_{Zn}-[$Zn_2L^h{}_3$][ClO_4]$_4$·$8H_2O$ (Δ-6h)

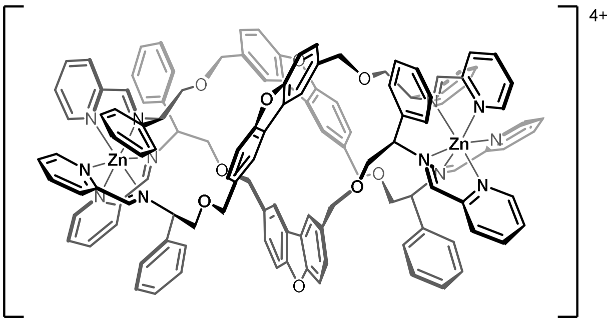

Δ_{Zn}-[Zn_2**L**$^h{}_3$]$^{4+}$

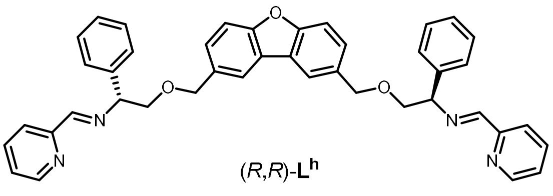

(*R*,*R*)-**L**^{h}

Yield: 53 %.

^{1}H NMR (500 MHz, 298 K, CD_3CN): δ_H 8.95 (6H, s, Ph), 8.47 (6H, s, HC=N), 7.77 (6H, t, $^3J_{HH}$ = 7.8 Hz, Py), 7.72 (6H, d, $^3J_{HH}$ = 8.4 Hz, Ph), 7.64 (6H, d, $^3J_{HH}$ = 8.4 Hz, Ph), 7.48 (6H, d, $^3J_{HH}$ = 4.7 Hz, Py), 7.27 (6H, dd , $^3J_{HH}$ = 7.2 Hz, 5.2 Hz, Py), 7.20 (6H, d, $^3J_{HH}$ = 7.8 Hz, Py), 6.93 (6H, t, $^3J_{HH}$ = 7.4 Hz, Ph), 6.75 (12H, t, $^3J_{HH}$ = 7.6 Hz, Ph), 6.18 (12H, d, $^3J_{HH}$ = 7.6 Hz, Ph), 5.26 (6H, dd, $^3J_{HH}$ = 10.5 Hz, $^3J_{HH}$ = 2.6 Hz, CH), 4.44 (6H, d, $^2J_{HH}$ = 9.6 Hz, OC*H*$_2$Ph), 3.81 (6H, d, $^2J_{HH}$ = 9.6 Hz, OC*H*$_2$Ph), 3.70 (6H, t, $^2J_{HH}$ /$^3J_{HH}$ = 10.9 Hz, OC*H*$_2$CH), 2.59 (6H, d, $^2J_{HH}$ = 9.9 Hz, OC*H*$_2$CH).

^{13}C{^{1}H} NMR (125 MHz, 298 K, CD_3CN): δ_C 163.3 (C=N), 157.5 (Ar), 148.7 (Ar), 147.1 (Ar), 142.6 (Ar), 135.6 (Ar), 134.1 (Ar), 130.7 (Ar), 130.5 (Ar), 129.6 (Ar), 129.4 (Ar), 129.0 (Ar), 127.3 (Ar), 125.0 (Ar), 122.0 (Ar), 112.8 (Ar), 74.2 (O*C*H$_2$Ph), 72.8 (O*C*H$_2$CH), 67.9 (CH).

MS (ESI): m/z 667.3 [**L**^{i}+Na]$^+$, 645.3 [**L**^{i}+H]$^+$.

Elemental analysis found (calculated for $C_{126}H_{108}Cl_4N_{12}O_{25}Zn_2$·$8H_2O$): % C 58.02 (58.05) H 4.17 (4.79) N 6.32 (6.45).

Synthesis and characterisation of [Fe_2L_3]Cl_4 complexes - General procedure

The appropriate optically pure diamine (3.0 eq.) and 2-pyridinecarboxaldehyde (6.0 eq.) were dissolved in methanol (50 ml) and stirred for 2 h at ambient temperature to form a yellow solution containing the ligand. Anhydrous iron (II) chloride (2.0 eq.) was added, and an immediate colour change to deep purple was observed. The solution was then heated at reflux

(80 °C) for 48 h. After filtering through fluted filter paper, the solvent was removed under reduced pressure to give the desired product as a dark purple solid, which was dried overnight at 50 °C *in vacuo.* Note that the presence of water of crystallisation in these compounds was confirmed by NMR and IR spectroscopy and the absence of other solvents was confirmed also by NMR. The hydration number was then determined by thermogravimetric analysis (section 1.6) and the relevant mass loss was correlated with microanalytical data.

Δ_{Fe}-[$Fe_2L^a_3$]Cl_4·6H_2O (Δ-5a)

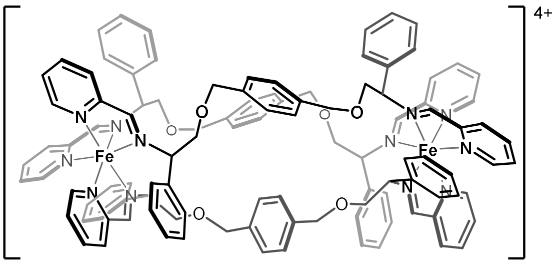

Δ_{Fe}-[Fe_2**L**$^a_3$]$^{4+}$

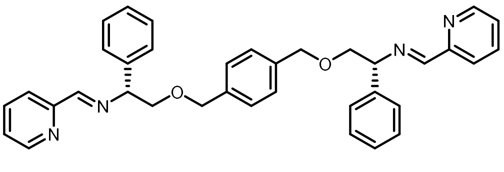

(*R*,*R*)-**L**^{a}

Yield: 1.18 g, 0.58 mmol, 92 % (0.7 g (1.86 mmol) of diamine used).

^{1}H NMR (400 MHz, 298 K, CD_3OD): δ_H 9.30 (6H, s, HC=N), 7.80 (6H, t, $^3J_{HH}$ = 7.0 Hz, Py), 7.58 (6H, d, $^3J_{HH}$ = 7.0 Hz, Py), 7.55 (12H, s, Ph), 7.29 (6H, t, $^3J_{HH}$ = 7.0 Hz, Py), 7.11 (6H, t, $^3J_{HH}$ = 7.5 Hz, Ph), 7.02 (12H, t, $^3J_{HH}$ = 7.5 Hz, Ph), 6.85-6.80 (18H, m, Py/Ph), 5.97 (6H, dd, $^3J_{HH}$ = 11.0 Hz, $^3J_{HH}$ = 3.5 Hz, CH), 5.12 (6H, d, $^2J_{HH}$ = 13.0 Hz, OC*H*$_2$Ph), 4.61 (6H, d, $^2J_{HH}$ = 13.0 Hz, OC*H*$_2$Ph), 4.34 (6H, t, $^2J_{HH}$/$^3J_{HH}$ = 11.0 Hz, OC*H*$_2$CH), 3.58 (6H, dd, $^2J_{HH}$ = 11.0 Hz, $^3J_{HH}$ = 3.5 Hz, OC*H*$_2$CH).

^{13}C{^{1}H} NMR (125 MHz, 298 K, CD_3OD): δ_C 173.3 (C=N), 160.4 (Ar), 154.6 (Ar), 139.9 (Ar), 138.7 (Ar), 136.5 (Ar), 130.5 (Ar), 129.5 (Ar), 129.4 (Ar), 128.9 (Ar), 127.1 (Ar), 73.7 (O*C*H$_2$Ph), 73.1 (O*C*H$_2$CH), 73.0 (CH).

MS (ESI): m/z 443.8 [Fe_2**L**$^a_3$]$^{4+}$, 577.3 [**L**^{a}+Na]$^+$.

FTIR: ν cm^{-1} 3346 br, 3024 w, 2860 m, 1635 w, 1612 m, 1591 w, 1494 w, 1472 m, 1452 m, 1358 w, 1298 w, 1241 w, 1104 m, 1074 s, 1019 m, 1000 m, 936 w, 837 w, 757 s, 700 s.

Elemental analysis found (calculated for $C_{108}H_{106}Cl_4Fe_2N_{12}O_6{\cdot}6H_2O$): % C 64.08 (63.91) H 5.40 (5.86) N 7.99 (8.28).

Λ_{Fe}-[Fe$_2$L^a$_3$]Cl$_4$·6H$_2$O (Λ-5a)

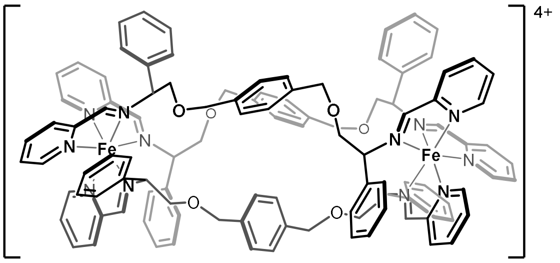

Λ_{Fe}-[Fe$_2$**L**^{a}$_3$]$^{4+}$

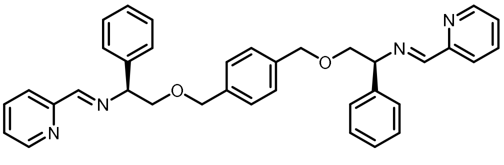

(*S*,*S*)-**L**^{a}

Yield: 1.18 g, 0.58 mmol, 93 % (0.7 g (1.86 mmol) of diamine used).

^{1}H NMR (400 MHz, 298 K, CD$_3$OD): δ_H 9.30 (6H, s, HC=N), 7.80 (6H, t, $^3J_{HH}$ = 7.0 Hz, Py), 7.58 (6H, d, $^3J_{HH}$ = 7.0 Hz, Py), 7.55 (12H, s, Ph), 7.29 (6H, t, $^3J_{HH}$ = 7.0 Hz, Py), 7.11 (6H, t, $^3J_{HH}$ = 7.5 Hz, Ph), 7.02 (12H, t, $^3J_{HH}$ = 7.5 Hz, Ph), 6.85-6.80 (18H, m, Py/Ph), 5.97 (6H, dd, $^3J_{HH}$ = 11.0 Hz, $^3J_{HH}$ = 3.5 Hz, CH), 5.12 (6H, d, $^2J_{HH}$ = 13.0 Hz, OC*H*$_2$Ph), 4.61 (6H, d, $^2J_{HH}$ = 13.0 Hz, OC*H*$_2$Ph), 4.34 (6H, t, $^2J_{HH}$/$^3J_{HH}$ = 11.0 Hz, OC*H*$_2$CH), 3.58 (6H, dd, $^2J_{HH}$ = 11.0 Hz, $^3J_{HH}$ = 3.5 Hz, OC*H*$_2$CH).

^{13}C{^{1}H} NMR (125 MHz, 298 K, CD$_3$OD): δ_C 173.3 (C=N), 160.4 (Ar), 154.6 (Ar), 139.9 (Ar), 138.7 (Ar), 136.5 (Ar), 130.5 (Ar), 129.5 (Ar), 129.4 (Ar), 128.9 (Ar), 127.1 (Ar), 73.7 (O*C*H$_2$Ph), 73.1 (O*C*H$_2$CH), 73.0 (CH).

MS (ESI): m/z 443.8 [Fe$_2$**L**^{a}$_3$]$^{4+}$, 577.3 [**L**^{a}+Na]$^+$.

FTIR: ν cm^{-1} 3346 br, 3024 w, 2860 m, 1635 w, 1612 m, 1591 w, 1494 w, 1472 m, 1452 m, 1358 w, 1298 w, 1241 w, 1104 m, 1074 s, 1019 m, 1000 m, 936 w, 837 w, 757 s, 700 s.

Elemental analysis found (calculated for $C_{108}H_{106}Cl_4Fe_2N_{12}O_6{\cdot}6H_2O$): % C 63.41 (63.91) H 5.61 (5.86) N 7.96 (8.28).

Δ_{Fe}-[Fe$_2$L^b$_3$]Cl$_4$·6.5H$_2$O (Δ-5b)

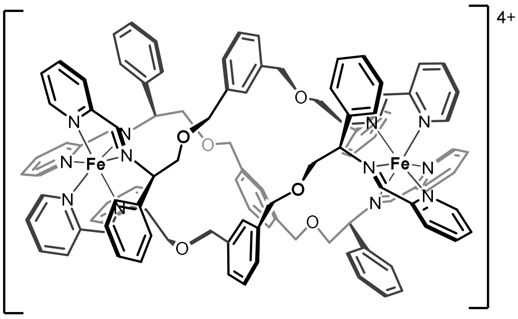

Δ_{Fe}-[Fe_2**L**$^b{}_3$]$^{4+}$

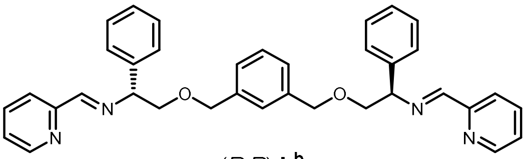

(*R*,*R*)-**L**^{b}

Yield: 1.21 g, 0.59 mmol, 96 % (0.7 g (1.86 mmol) of diamine used).
^{1}H NMR (400 MHz, 298 K, CD_3OD): δ_H 9.22 (6H, s, HC=N), 8.37 (3H, s, Ph), 7.73 (6H, t, $^3J_{HH}$ = 7.6 Hz, Py), 7.66 (6H, d, $^3J_{HH}$ = 7.6 Hz, Py), 7.52-7.47 (9H, m, Ph), 7.22 (6H, t, $^3J_{HH}$ = 6.5 Hz, Py), 7.02 (6H, t, $^3J_{HH}$ = 7.3 Hz, Ph), 6.86 (12H, t, $^3J_{HH}$ = 6.5 Hz, Ph), 6.78 (6H, d, $^3J_{HH}$ = 5.3 Hz, Py), 6.55 (12H, br s, Ph), 5.86 (6H, dd, $^3J_{HH}$ = 11.0 Hz, $^3J_{HH}$ = 2.1 Hz, CH), 5.03 (6H, d, $^2J_{HH}$ = 10.8 Hz, OCH_2Ph), 4.40 (6H, t, $^2J_{HH}$/$^3J_{HH}$ = 11.4 Hz, OCH_2CH), 3.05 (6H, dd, $^2J_{HH}$ = 11.4 Hz, $^3J_{HH}$ = 2.5 Hz, OCH_2CH). The presence of water (δ_H 4.91-4.85) obscures the second OCH_2Ph peak, which could nonetheless be detected by 2D-NMR (HSQC).
^{13}C NMR (125 MHz, 298 K, CD_3OD): δ_C 172.9 (C=N), 160.3 (Ar), 154.5 (Ar), 139.9 (Ar), 139.4 (Ar), 135.8 (Ar), 130.5 (Ar), 130.4 (Ar), 130.2 (Ar), 129.3 (Ar), 128.8 (Ar), 75.9 (O*C*H_2Ph), 74.4 (O*C*H_2CH), 72.4 (CH).

MS (ESI): m/z 443.8 [Fe_2**L**$^b{}_3$]$^{4+}$, 577.3 [**L**^{b}+Na]$^+$.
FTIR: ν cm^{-1} 3356 br, 3028 w, 2864 w, 1612 w, 1590 w, 1494 w, 1472 m, 1451 m, 1385 w, 1357 w, 1298 m, 1240 w, 1156 w, 1105 m, 1072 s, 1003 m, 930 w, 887 w, 834 w, 756 s.
Elemental analysis found (calculated for $C_{108}H_{106}Cl_4Fe_2N_{12}O_6\cdot6.5H_2O$): % C 63.65 (63.63) H 5.84 (5.88) N 8.09 (8.24).

Λ_{Fe}-[Fe_2L$^b{}_3$]$Cl_4\cdot6.5H_2O$ (Λ-5b)

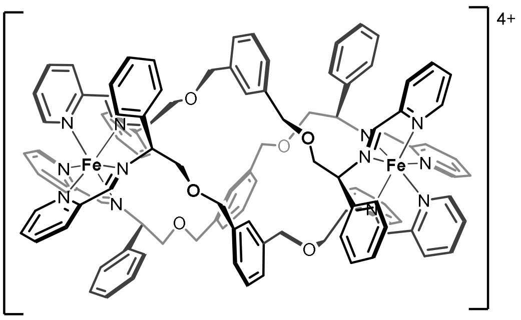

Λ_{Fe}-[Fe_2**L**$^{b}{}_3$]$^{4+}$

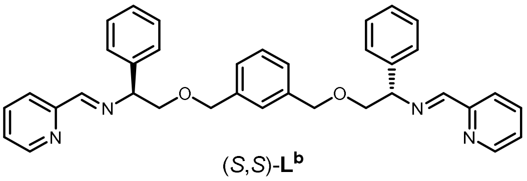

(S,S)-**L**^{b}

Yield: 1.19 g, 0.58 mmol, 94 % (0.7 g (1.86 mmol) of diamine used).

^{1}H NMR (400 MHz, 298 K, CD_3OD): δ_H 9.22 (6H, s, HC=N), 8.37 (3H, s, Ph), 7.73 (6H, t, $^3J_{HH}$ = 7.6 Hz, Py), 7.66 (6H, d, $^3J_{HH}$ = 7.6 Hz, Py), 7.52-7.47 (9H, m, Ph), 7.22 (6H, t, $^3J_{HH}$ = 6.5 Hz, Py), 7.02 (6H, t, $^3J_{HH}$ = 7.3 Hz, Ph), 6.86 (12H, t, $^3J_{HH}$ = 6.5 Hz, Ph), 6.78 (6H, d, $^3J_{HH}$ = 5.3 Hz, Py), 6.55 (12H, br s, Ph), 5.86 (6H, dd, $^3J_{HH}$ = 11.0 Hz, $^3J_{HH}$ = 2.1 Hz, CH), 5.03 (6H, d, $^2J_{HH}$ = 10.8 Hz, OCH_2Ph), 4.40 (6H, t, $^2J_{HH}$/$^3J_{HH}$ = 11.4 Hz, OCH_2CH), 3.05 (6H, dd, $^2J_{HH}$ = 11.4 Hz, $^3J_{HH}$ = 2.5 Hz, OCH_2CH). The presence of water (δ_H 4.91-4.85) obscures the second OCH_2Ph peak, which could nonetheless be detected by 2D-NMR (HSQC).

^{13}C{^{1}H} NMR (125 MHz, 298 K, CD_3OD): δ_C 172.9 (C=N), 160.3 (Ar), 154.5 (Ar), 139.9 (Ar), 139.4 (Ar), 135.8 (Ar), 130.5 (Ar), 130.4 (Ar), 130.2 (Ar), 129.3 (Ar), 128.8 (Ar), 75.9 (O*C*H_2Ph), 74.4 (O*C*H_2CH), 72.4 (CH).

MS (ESI): m/z 443.8 [Fe_2**L**$^{b}{}_3$]$^{4+}$, 577.3 [**L**^{b}+Na]$^{+}$.

FTIR: ν cm^{-1} 3356 br, 3028 w, 2864 w, 1612 w, 1590 w, 1494 w, 1472 m, 1451 m, 1385 w, 1357 w, 1298 m, 1240 w, 1156 w, 1105 m, 1072 s, 1003 m, 930 w, 887 w, 834 w, 756 s.

Elemental analysis found (calculated for $C_{108}H_{106}Cl_4Fe_2N_{12}O_6{\cdot}6.5H_2O$): % C 64.08 (63.63) H 5.68 (5.88) N 7.98 (8.24).

Δ_{Fe}-[Fe_2L$^{b'}{}_3$]$Cl_4{\cdot}8H_2O$ (Δ-5b′)

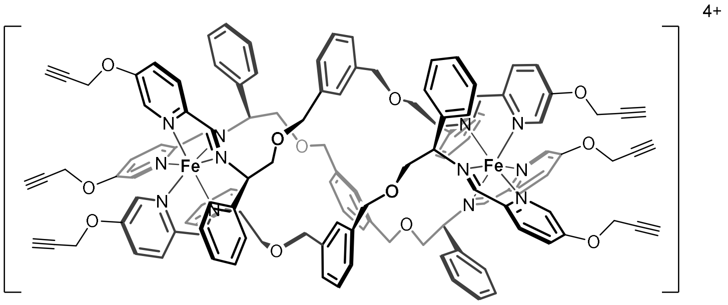

Δ_{Fe}-[$Fe_2$$\mathbf{L}^{b'}_3$]$^{4+}$

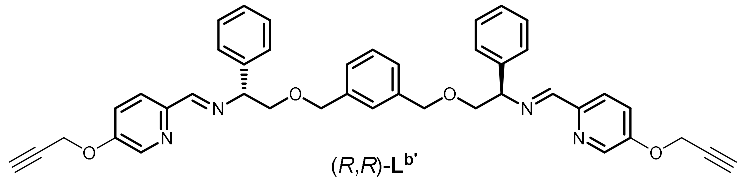

(R,R)-$\mathbf{L}^{b'}$

Yield = 0.275 g, 0.12 mmol, 65 %. (0.2 g (0.53 mmol) of diamine used).

^{1}H NMR (500 MHz, 298 K, CD_3OD) δ_H 9.16 (6H, s, N=CH), 8.37 (3H, s, Ph), 7.67 (6H, d, $^3J_{HH}$ = 7.5 Hz, Py), 7.51 (18H, m, Ph), 7.33 (6H, dd, J = 9.0 Hz, J = 2.0 Hz, Py), 7.05 (6H, t, $^3J_{HH}$ = 7.5 Hz, Ph), 6.93 (12H, t, $^3J_{HH}$ = 7.5 Hz, Ph), 6.61 (9H, br s, Ph), 6.47 (6H, d, $^4J_{HH}$= 2.0 Hz, Py), 5.78 (6H, dd, $^3J_{HH}$ = 11.0 Hz, $^4J_{HH}$ = 2.0 Hz, CH), 5.03 (6H, d, $^2J_{HH}$ = 11.0 Hz, OCH_2Ph), 4.71 (12H, q, $^2J_{HH}$ = 16.5 Hz, CH_2C≡C), 4.40 (6H, t, $^2J_{HH}$/$^3J_{HH}$ = 11.5 Hz, OCH_2CH), 3.20 (6H, s, C≡CH), 3.06 (6H, dd, $^2J_{HH}$ = 11.0 Hz, $^3J_{HH}$ = 3.0 Hz, OCH_2CH). The presence of water (δ_H 4.91-4.85) obscures the second OCH_2Ph peak, but this was detected by 2D-NMR (HSQC).

^{13}C{^{1}H} NMR (125 MHz, 298K, CD_3OD) δ_C ppm 170.1 (C=N), 155.7 (Ar), 152.6 (Ar), 141.0 (Ar), 138.1 (Ar), 134.81 (Ar), 129.8 (Ar), 129.0 (Ar), 128.6 (Ar), 127.9 (Ar), 127.4 (Ar), 123.9 (Ar), 78.2 (C≡*C*H),76.6 (*C*≡CH), 74.5 (CH_2Ph), 73.0 (CH_2*C*H), 70.3 (CH), 55.9 (*C*H_2C≡C).

MS (ESI): m/z 525.0 [$Fe_2$$\mathbf{L}^{b'}_3$]$^{4+}$, 685.4 [$\mathbf{L}^{b'}$+Na]$^+$

FTIR: ν cm^{-1} 3348 br, 3029 w, 2866 w, 1665 w, 1591 w, 1495 m, 1452 m, 1360 w, 1278 m, 1233 m, 1156 w, 1107 m, 1075 s, 1003 m, 928 w, 889 w, 843 w, 757 s.

Elemental analysis found (calculated for $C_{126}H_{144}Cl_4Fe_2N_{12}O_{12}\cdot 8H_2O$) % C 63.51 (63.43), H 4.84 (5.49), N 6.92 (7.04)

Λ_{Fe}-[$Fe_2L^{b'}{}_3$]$Cl_4\cdot 8H_2O$ (Λ-5b′)

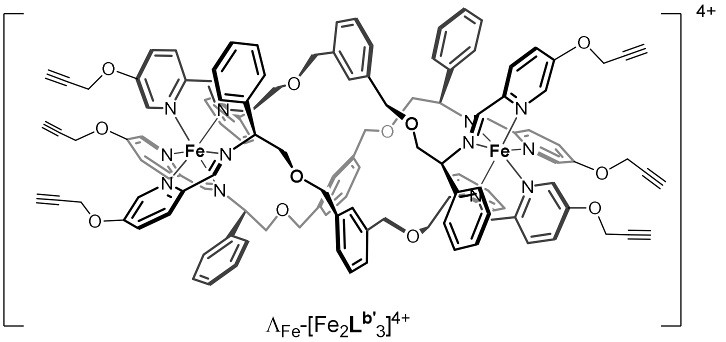

Λ_{Fe}-[Fe_2**L**$^{b'}{}_3$]$^{4+}$

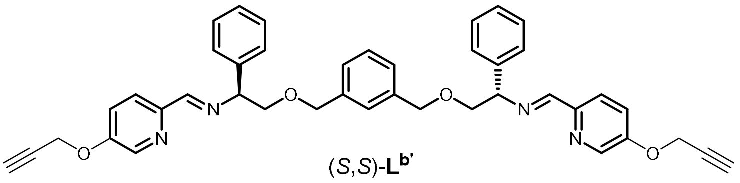

(S,S)-**L**$^{b'}$

Yield: 0.32 g, 0.13 mmol, 75 %. (0.2 g (0.53 mmol) of diamine used).

^{1}H NMR (500 MHz, 298 K, CD_3OD) δ_H 9.16 (6H, s, N=CH), 8.37 (3H, s, Ph), 7.67 (6H, d, $^3J_{HH}$ = 7.5 Hz, Py), 7.51 (18H, m, Ph), 7.33 (6H, dd, $^3J_{HH}$ = 9.0 Hz, $^4J_{HH}$ = 2.0 Hz, Py), 7.05 (6H, t, $^3J_{HH}$ = 7.5 Hz, Ph), 6.93 (12H, t, $^3J_{HH}$ = 7.5 Hz, Ph), 6.61 (9H, br s, Ph), 6.47 (6H, d, $^4J_{HH}$ = 2.0 Hz, Py), 5.78 (6H, dd, $^3J_{HH}$ = 11.0 Hz, $^4J_{HH}$ = 2.0 Hz, CH), 5.03 (6H, d, $^2J_{HH}$ = 11.0 Hz, OCH_2Ph), 4.71 (12H, q, $^2J_{HH}$ = 16.5 Hz, CH_2C≡C), 4.39 (6H, t, $^2J_{HH}$/$^3J_{HH}$ = 11.5 Hz, OCH_2CH), 3.20 (6H, s, C≡CH), 3.06 (6H, dd, $^2J_{HH}$ = 11.0 Hz, $^3J_{HH}$ = 3.0 Hz, OCH_2CH). The presence of water (δ_H 4.91-4.85) obscures the second OCH_2Ph peak, but this was detected by 2D-NMR (HSQC).

^{13}C{^{1}H} NMR (125 MHz, 298K, CD_3OD) δ_C ppm 170.1 (C=N), 155.7 (Ar), 152.6 (Ar), 141.1 (Ar), 138.1 (Ar), 134.81 (Ar), 129.8 (Ar), 129.1 (Ar), 128.6 (Ar), 127.9 (Ar), 127.4 (Ar), 123.9 (Ar), 78.1 (C≡*C*H),76.6 (*C*≡CH), 74.5 (CH_2Ph), 73.0 (CH_2*C*H), 70.3 (CH), 55.9 (*C*H_2C≡C).

MS (ESI): m/z 525.0 [Fe_2**L**$^{b'}{}_3$]$^{4+}$, 685.4 [**L**$^{b'}$+Na]$^+$

FTIR: ν cm^{-1} 3348 br, 3029 w, 2866 w, 1665 w, 1591 w, 1495 m, 1452 m, 1360 w, 1278 m, 1233 m, 1156 w, 1107 m, 1075 s, 1003 m, 928 w, 889 w, 843 w, 757 s.

Elemental analysis found (calculated for $C_{126}H_{144}Cl_4Fe_2N_{12}O_{12}\cdot6H_2O$) % C 63.55 (63.58), H 5.00 (6.61), N 6.93 (7.06)

Δ_{Fe}-[$Fe_2L^c_3$]$Cl_4\cdot12H_2O$ (Δ-5c)

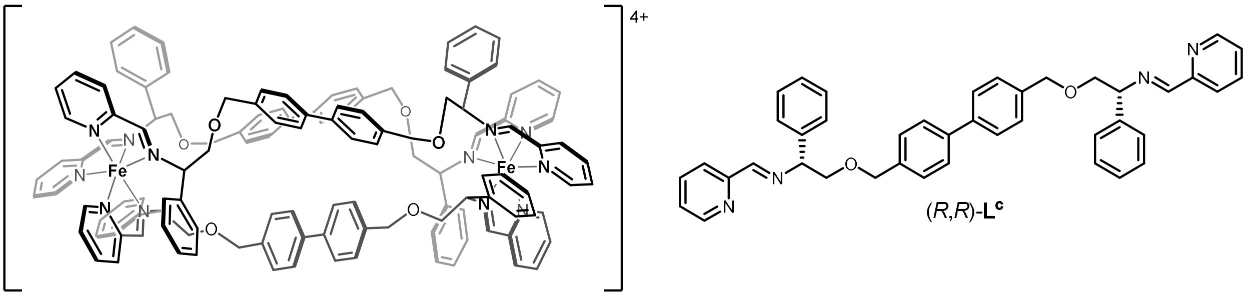

Δ_{Fe}-[Fe_2**L**$^c_3$]$^{4+}$

Yield: 0.364 g, 0.15 mmol, 84 % (0.25 g (0.55 mmol) of diamine used).

^{1}H NMR (500 MHz, 298 K, CD_3OD): δ_H 9.39 (6H, s, HC=N), 7.82 (6H, t, $^3J_{HH}$ = 6.8 Hz, Py), 7.62 (6H, d, $^3J_{HH}$ = 6.3 Hz, Py), 7.33-7.26 (18H, m, Ph/Py), 7.16-7.06 (30H, m, Ph), 6.92-6.87 (18H, m, Ph/Py), 6.09 (6H, d, $^3J_{HH}$ = 9.8 Hz, CH), 5.21 (6H, d, $^2J_{HH}$ = 9.6 Hz, OC*H*$_2$Ph), 4.70 (6H, d, $^2J_{HH}$ = 13.7 Hz, OC*H*$_2$Ph/H_2O), 4.39 (6H, t, $^2J_{HH}/^3J_{HH}$ = 10.6 Hz, OC*H*$_2$CH), 3.82 (6H, d, $^2J_{HH}$ = 9.8 Hz, OC*H*$_2$CH).

^{13}C{^{1}H} NMR (100 MHz, 298 K, CD_3OD): δ_C 173.5 (C=N), 160.6 (Ar), 154.7 (Ar), 141.4 (Ar), 139.9 (Ar), 137.7 (Ar), 136.5 (Ar), 130.6 (Ar), 130.5 (Ar), 129.5 (Ar), 128.9 (Ar) 128.6 (Ar), 127.1 (Ar), 73.1 (O*C*H$_2$Ph), 72.9 (CH), 72.8 (O*C*H$_2$CH).

MS (ESI): m/z 500.9 [Fe_2**L**$^d_3$]$^{4+}$.

FTIR: ν cm^{-1} 3350 br, 2862 w, 1612 w, 1556 w, 1494 m, 1471 m, 1451 m, 1382 w, 1360 w, 1298 w, 1207 w, 1102 m, 1073 s, 1023 m, 1004 m, 934 w, 804 m, 758 s, 700s.

Elemental analysis found (calculated for $C_{126}H_{118}Cl_4Fe_2N_{12}O_6\cdot12H_2O$): % C 63.86 (63.96) H 5.46 (6.05) N 6.88 (7.10).

Λ_{Fe}-[$Fe_2L^c_3$]$Cl_4\cdot12H_2O$ (Λ-5c)

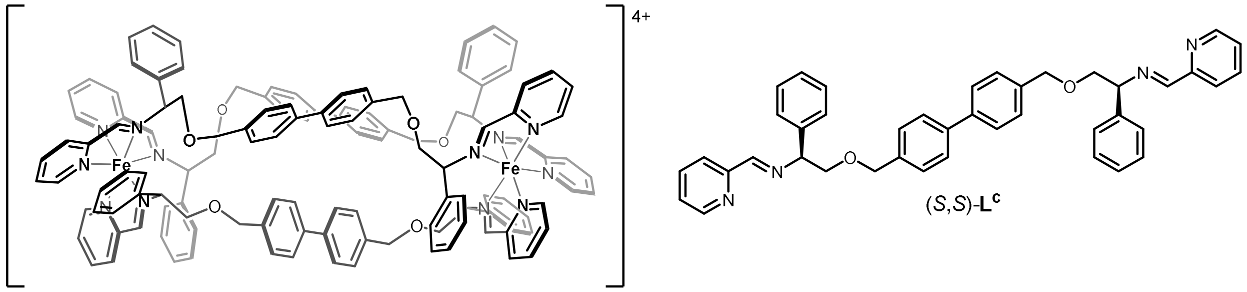

Λ_{Fe}-$[Fe_2\mathbf{L^c}_3]^{4+}$

Yield: 0.347 g, 0.15 mmol, 80 % (0.25 g (0.55 mmol) of diamine used).

^{1}H NMR (500 MHz, 298 K, CD_3OD): δ_H 9.39 (6H, s, HC=N), 7.82 (6H, t, $^3J_{HH}$ = 6.8 Hz, Py), 7.62 (6H, d, $^3J_{HH}$ = 6.3 Hz, Py), 7.33-7.26 (18H, m, Ph/Py), 7.16-7.06 (30H, m, Ph), 6.92-6.87 (18H, m, Ph/Py), 6.09 (6H, d, $^3J_{HH}$ = 9.8 Hz, CH), 5.21 (6H, d, $^2J_{HH}$ = 9.6 Hz, OCH_2Ph), 4.70 (6H, d, $^2J_{HH}$ = 13.7 Hz, OCH_2Ph/H_2O), 4.39 (6H, t, $^2J_{HH}$/$^3J_{HH}$ = 10.6 Hz, OCH_2CH), 3.82 (6H, d, $^2J_{HH}$ = 9.8 Hz, OCH_2CH).

^{13}C{^{1}H} NMR (100 MHz, 298 K, CD_3OD): δ_C 173.5 (C=N), 160.6 (Ar), 154.7 (Ar), 141.4 (Ar), 139.9 (Ar), 137.7 (Ar), 136.5 (Ar), 130.6 (Ar), 130.5 (Ar), 129.5 (Ar), 128.9 (Ar) 128.6 (Ar), 127.1 (Ar), 73.1 (O*C*H_2Ph), 72.9 (CH), 72.8 (O*C*H_2CH).

MS (ESI): m/z 500.9 $[Fe_2\mathbf{L^d}_3]^{4+}$.

FTIR: ν cm^{-1} 3350 br, 2862 w, 1612 w, 1556 w, 1494 m, 1471 m, 1451 m, 1382 w, 1360 w, 1298 w, 1207 w, 1102 m, 1073 s, 1023 m, 1004 m, 934 w, 804 m, 758 s, 700s.

Elemental analysis found (calculated for $C_{126}H_{118}Cl_4Fe_2N_{12}O_6 \cdot 12H_2O$): % C 63.97 (63.96) H 5.28 (6.05) N 6.86 (7.10).

Δ_{Fe}-$[Fe_2L^d_3]Cl_4 \cdot 10.5H_2O$ (Δ-5d)

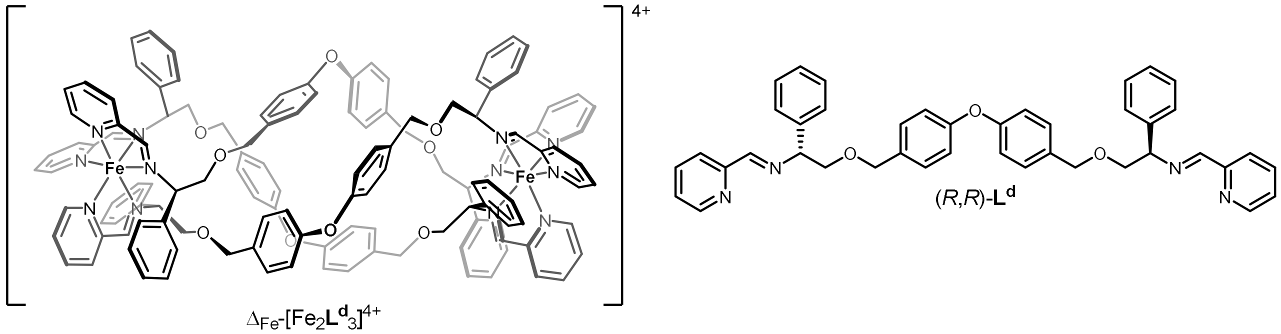

Δ_{Fe}-$[Fe_2\mathbf{L^d}_3]^{4+}$

Yield: 0.347 g, 0.15 mmol, 82 % (0.25 g (0.53 mmol) of diamine used).

^{1}H NMR (500 MHz, 298 K, CD_3OD): δ_H 9.33 (6H, s, HC=N), 7.76 (6H, t, $^{3}J_{HH}$ = 7.6 Hz, Py), 7.55 (6H, d, $^{3}J_{HH}$ = 7.6 Hz, Py), 7.45 (12H, d, $^{3}J_{HH}$ = 8.3 Hz, Ph), 7.25 (6H, t, $^{3}J_{HH}$ = 6.5 Hz, Py), 7.08 (6H, t, $^{3}J_{HH}$ = 7.3 Hz, Ph), 6.97 (12H, t, $^{3}J_{HH}$ = 7.5 Hz, Ph), 6.80-6.68 (30H, m, Py/Ph), 6.00 (6H, dd, $^{3}J_{HH}$ = 10.9 Hz, $^{3}J_{HH}$ = 1.8 Hz, CH), 5.03 (6H, d, $^{2}J_{HH}$ = 11.9 Hz, OCH_2Ph), 4.52 (6H, t, $^{2}J_{HH}$/$^{3}J_{HH}$ = 10.9 Hz, OCH_2CH), 4.40 (6H, d, $^{2}J_{HH}$ = 11.9 Hz, OCH_2Ph), 3.25 (6H, dd, $^{2}J_{HH}$ = 10.9 Hz, $^{3}J_{HH}$ = 2.0 Hz, OCH_2CH).

^{13}C{^{1}H} NMR (125 MHz, 298 K, CD_3OD): δ_C 172.8 (C=N), 160.4 (Ar), 158.4 (Ar), 154.5 (Ar), 139.8 (Ar), 136.4 (Ar), 134.2 (Ar), 130.5 (Ar), 130.4 (Ar), 130.1 (Ar), 129.4 (Ar), 128.8 (Ar), 127.0 (Ar), 74.1 (O*C*H_2Ph), 73.7 (O*C*H_2CH), 73.0 (CH).

MS (ESI): m/z 512.7 [Fe_2**L**$^{e}_{3}$]$^{4+}$.

FTIR: ν cm^{-1} 3339 br, 2860 w, 1601 m, 1500 s, 1472 m, 1451 m, 1385 w, 1358 w, 1299 w, 1231 s, 1166 w, 1103 m, 1077 s, 1013 m, 1001 m, 873 m, 836 m, 817 w, 758 s, 700 s.

Elemental analysis found (calculated for $C_{126}H_{118}Cl_4Fe_2N_{12}O_9 \cdot 10.5H_2O$): % C 63.21 (63.40) H 5.48 (5.87) N 6.73 (7.04).

Λ_{Fe}-[Fe_2L$^{d}_{3}$]Cl_4·10.5H_2O (Λ-5d)

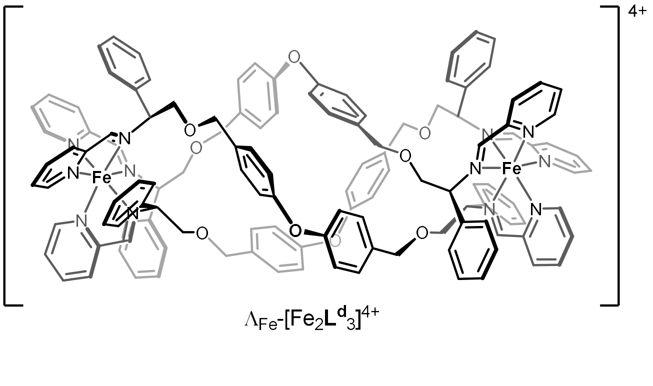

Λ_{Fe}-[Fe_2**L**$^{d}_{3}$]$^{4+}$

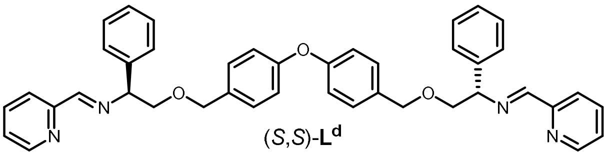

(*S*,*S*)-**L**^{d}

Yield: 0.332 g, 0.14 mmol, 78 % (0.25 g (0.53 mmol) of diamine used).

^{1}H NMR (500 MHz, 298 K, CD_3OD): δ_H 9.33 (6H, s, HC=N), 7.76 (6H, t, $^{3}J_{HH}$ = 7.6 Hz, Py), 7.55 (6H, d, $^{3}J_{HH}$ = 7.6 Hz, Py), 7.45 (12H, d, $^{3}J_{HH}$ = 8.3 Hz, Ph), 7.25 (6H, t, $^{3}J_{HH}$ = 6.5 Hz, Py), 7.08 (6H, t, $^{3}J_{HH}$ = 7.3 Hz, Ph), 6.97 (12H, t, $^{3}J_{HH}$ = 7.5 Hz, Ph), 6.80-6.68 (30H, m, Py/Ph), 6.00

(6H, dd, $^3J_{HH}$ = 10.9 Hz, $^3J_{HH}$ = 1.8 Hz, CH), 5.03 (6H, d, $^2J_{HH}$ = 11.9 Hz, OC*H*$_2$Ph), 4.52 (6H, t, $^2J_{HH}$/$^3J_{HH}$ = 10.9 Hz, OC*H*$_2$CH), 4.40 (6H, d, $^2J_{HH}$ = 11.9 Hz, OC*H*$_2$Ph), 3.25 (6H, dd, $^2J_{HH}$ = 10.9 Hz, $^3J_{HH}$ = 2.0 Hz, OC*H*$_2$CH).

$^{13}C\{^1H\}$ NMR (125 MHz, 298 K, CD_3OD): δ_C 172.8 (C=N), 160.4 (Ar), 158.4 (Ar), 154.5 (Ar), 139.8 (Ar), 136.4 (Ar), 134.2 (Ar), 130.5 (Ar), 130.4 (Ar), 130.1 (Ar), 129.4 (Ar), 128.8 (Ar), 127.0 (Ar), 74.1 (O*C*H$_2$Ph), 73.7 (O*C*H$_2$CH), 73.0 (CH).

MS (ESI): m/z 512.7 [Fe_2**L**$^e{}_3$]$^{4+}$.

FTIR: ν cm^{-1} 3339 br, 2860 w, 1601 m, 1500 s, 1472 m, 1451 m, 1385 w, 1358 w, 1299 w, 1231 s, 1166 w, 1103 m, 1077 s, 1013 m, 1001 m, 873 m, 836 m, 817 w, 758 s, 700 s.

Elemental analysis found (calculated for $C_{126}H_{118}Cl_4Fe_2N_{12}O_9\cdot 10.5H_2O$): % C 63.05 (63.40) H 5.17 (5.87) N 6.71 (7.04).

Δ_{Fe}-[$Fe_2L^e{}_3$]$Cl_4\cdot 11H_2O$ (Δ-5e)

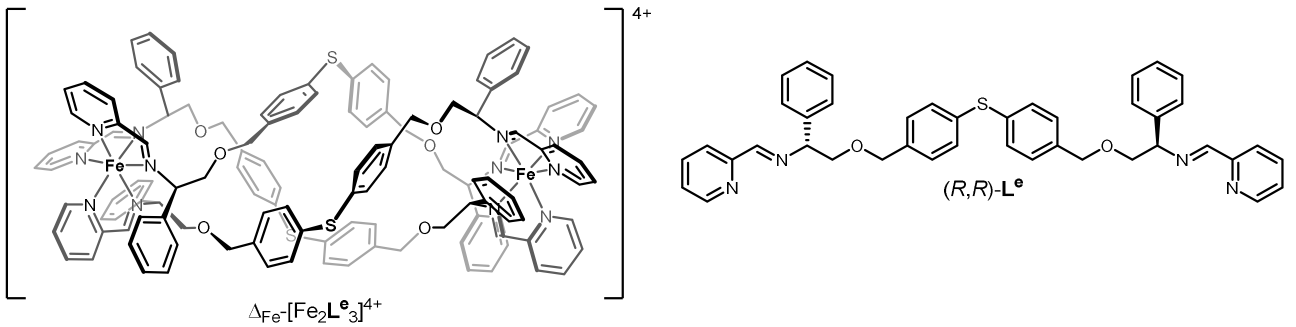

Δ_{Fe}-[Fe_2**L**$^e{}_3$]$^{4+}$

Yield: 0.359 g, 0.15 mmol, 85 % (0.25 g (0.52 mmol) of diamine used).

^1H NMR (500 MHz, 298 K, CD_3OD): δ_H 9.34 (6H, s, HC=N), 7.74 (6H, t, $^3J_{HH}$ = 7.4 Hz, Py), 7.53 (6H, d, $^3J_{HH}$ = 7.4 Hz, Py), 7.45 (12H, d, $^3J_{HH}$ = 7.8 Hz, Ph), 7.25 (6H, t, $^3J_{HH}$ = 6.4 Hz, Py), 7.08-7.04 (18H, m, Ph), 6.96 (12H, t, $^3J_{HH}$ = 7.0 Hz, Ph), 6.76-6.66 (18H, m, Ph/Py), 5.93 (6H, d, $^3J_{HH}$ = 10.7 Hz, CH), 4.99 (6H, d, $^2J_{HH}$ = 11.6 Hz, OC*H*$_2$Ph), 4.52 (6H, t, $^2J_{HH}$/$^3J_{HH}$ = 10.7 Hz, OC*H*$_2$CH), 4.38 (6H, d, $^2J_{HH}$ = 11.7 Hz, OC*H*$_2$Ph), 3.04 (6H, d, $^2J_{HH}$ = 10.7 Hz, OC*H*$_2$CH).

$^{13}C\{^1H\}$ NMR (125 MHz, 298 K, CD_3OD): δ_C 172.6 (C=N), 160.3 (Ar), 154.4 (Ar), 139.8 (Ar), 138.1 (Ar), 136.5 (Ar), 136.2 (Ar), 131.4 (Ar), 130.5 (Ar), 130.4 (Ar), 129.7 (Ar), 129.5 (Ar), 128.8 (Ar), 127.1 (Ar), 73.9 (O*C*H$_2$Ph), 73.7 (O*C*H$_2$CH), 73.0 (CH).

MS (ESI): m/z 524.7 $[Fe_2\mathbf{L^f}_3]^{4+}$.

FTIR: ν cm^{-1} 3360 br, 3027 w, 2862 w, 1613 w, 1592 w, 1492 m, 1472 m, 1452 m, 1384 w, 1353 w, 1299 w, 1242 w, 1102 m, 1079 s, 1015 s, 1001 m, 841 w, 804 m, 758 s, 700 s.

Elemental analysis found (calculated for $C_{126}H_{118}Cl_4Fe_2N_{12}O_6S_3\cdot11H_2O$): % C 61.66 (61.92) H 5.20 (5.77) N 6.61 (6.88).

Λ_{Fe}-$[Fe_2L^e{}_3]Cl_4\cdot11H_2O$ (Λ-5e)

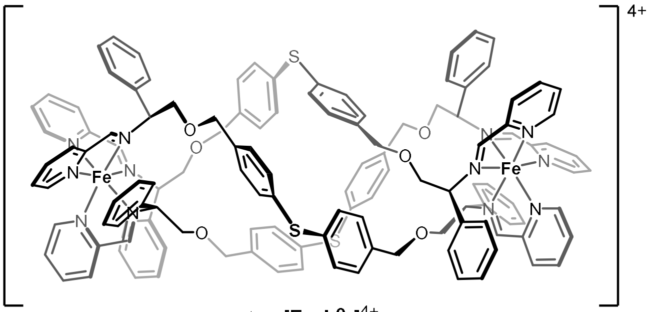

Λ_{Fe}-$[Fe_2\mathbf{L^e}_3]^{4+}$

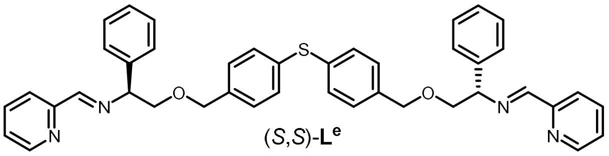

(*S*,*S*)-$\mathbf{L^e}$

Yield: 0.29 g, 0.12 mmol, 69 % (0.25 g (0.52 mmol) of diamine used).

^{1}H NMR (500 MHz, 298 K, CD_3OD): δ_H 9.34 (6H, s, HC=N), 7.74 (6H, t, $^3J_{HH}$ = 7.4 Hz, Py), 7.53 (6H, d, $^3J_{HH}$ = 7.4 Hz, Py), 7.45 (12H, d, $^3J_{HH}$ = 7.8 Hz, Ph), 7.25 (6H, t, $^3J_{HH}$ = 6.4 Hz, Py), 7.08-7.04 (18H, m, Ph), 6.96 (12H, t, $^3J_{HH}$ = 7.0 Hz, Ph), 6.76-6.66 (18H, m, Ph/Py), 5.93 (6H, d, $^3J_{HH}$ = 10.7 Hz, CH), 4.99 (6H, d, $^2J_{HH}$ = 11.6 Hz, OCH_2Ph), 4.52 (6H, t, $^2J_{HH}$/$^3J_{HH}$ = 10.7 Hz, OCH_2CH), 4.38 (6H, d, $^2J_{HH}$ = 11.7 Hz, OCH_2Ph), 3.04 (6H, d, $^2J_{HH}$ = 10.7 Hz, OCH_2CH).

^{13}C{^{1}H} NMR (125 MHz, 298 K, CD_3OD): δ_C 172.6 (C=N), 160.3 (Ar), 154.4 (Ar), 139.8 (Ar), 138.1 (Ar), 136.5 (Ar), 136.2 (Ar), 131.4 (Ar), 130.5 (Ar), 130.4 (Ar), 129.7 (Ar), 129.5 (Ar), 128.8 (Ar), 127.1 (Ar), 73.9 (O*C*H_2Ph), 73.7 (O*C*H_2CH), 73.0 (CH).

MS (ESI): m/z 524.7 $[Fe_2\mathbf{L^f}_3]^{4+}$.

FTIR: ν cm^{-1} 3360 br, 3027 w, 2862 w, 1613 w, 1592 w, 1492 m, 1472 m, 1452 m, 1384 w, 1353 w, 1299 w, 1242 w, 1102 m, 1079 s, 1015 s, 1001 m, 841 w, 804 m, 758 s, 700 s.

Elemental analysis found (calculated for $C_{126}H_{118}Cl_4Fe_2N_{12}O_6S_3\cdot11H_2O$): % C 61.81 (61.92) H 5.22 (5.77) N 6.57 (6.88).

Δ_{Fe}-$[Fe_2L^f{}_3]Cl_4\cdot11H_2O$ (Δ-5f)

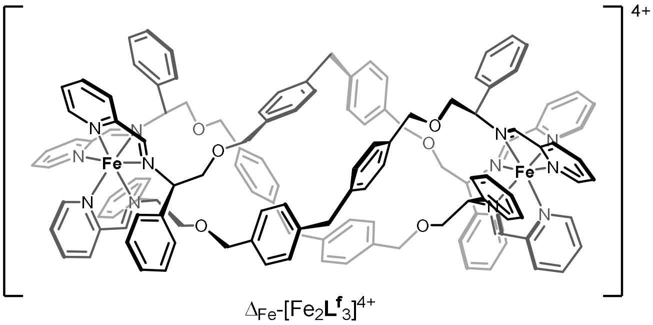

Δ_{Fe}-$[Fe_2\mathbf{L^f}_3]^{4+}$

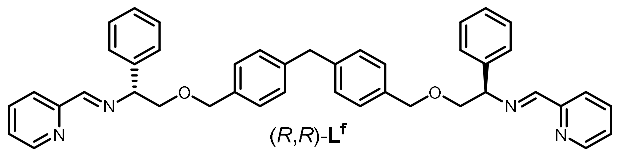

(*R*,*R*)-$\mathbf{L^f}$

Yield: 0.25 g, 0.10 mmol, 60 % (0.25 g (0.52 mmol) of diamine used).

^{1}H NMR (500 MHz, 298 K, CD_3OD): δ_H 9.28 (6H, s, HC=N), 7.75 (6H, t, $^3J_{HH}$ = 7.1 Hz, Py), 7.53 (6H, d, $^3J_{HH}$ = 7.1 Hz, Py), 7.36 (12H, d, $^3J_{HH}$ = 7.6 Hz, Ph), 7.23 (6H, t, $^3J_{HH}$ = 6.2 Hz, Py), 7.06 (6H, t, $^3J_{HH}$ = 7.0 Hz, Ph), 6.94 (12H, t, $^3J_{HH}$ = 7.1 Hz, Ph), 6.89 (12H, d, $^3J_{HH}$ = 7.6 Hz, Ph), 6.77 (6H, d, $^3J_{HH}$ = 5.0 Hz, Py), 6.69 (12H, d, $^3J_{HH}$ = 7.0 Hz, Ph), 5.96 (6H, d, $^3J_{HH}$ = 9.8 Hz, CH), 4.45 (6H, t, $^2J_{HH}$/$^3J_{HH}$ = 10.9 Hz, OCH_2CH), 4.37 (6H, d, $^2J_{HH}$ = 11.5 Hz, OCH_2Ph), 3.86 (6H, s, PhCH_2Ph), 3.23 (6H, d, $^2J_{HH}$ = 9.8 Hz, OCH_2CH).

^{13}C{^{1}H} NMR (125 MHz, 298 K, CD_3OD): δ_C 172.8 (C=N), 160.4 (Ar), 154.5 (Ar), 141.9 (Ar), 139.8 (Ar), 136.6 (Ar), 136.4 (Ar), 130.4 (Ar), 130.2 (Ar), 129.3 (Ar), 128.8 (Ar), 127.1 (Ar), 74.2 (O*C*H_2Ph), 73.9 (O*C*H_2CH), 72.9 (CH), 41.5 (Ph*C*H_2Ph).

MS (ESI): m/z 511.4 $[Fe_2\mathbf{L}_3]^{4+}$.

FTIR: ν cm^{-1} 3360 br, 3043 w, 2915 w, 2858 m, 1612 w, 1512 w, 1494 w, 1471 m, 1451 m, 1360 w, 1299 w, 1240 w, 1105 m, 1076 s, 1019 m, 1001 m, 757 s, 700 s.

Elemental analysis found (calculated for $C_{129}H_{124}Cl_4Fe_2N_{12}O_6{\cdot}11H_2O$): % C 65.34 (64.83) H 5.91 (6.16) N 6.52 (7.03).

Λ_{Fe}-$[Fe_2L^f{}_3]Cl_4{\cdot}11H_2O$ (Λ-5f)

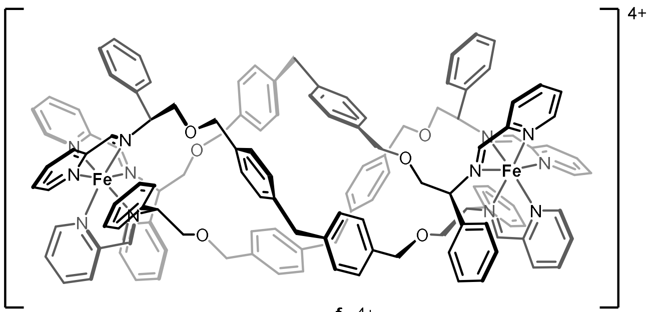

Λ_{Fe}-$[Fe_2\mathbf{L^f}_3]^{4+}$

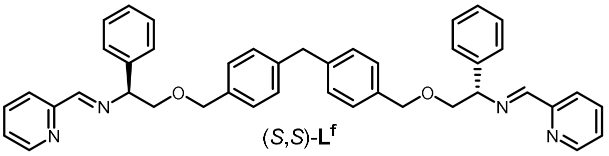

(*S*,*S*)-$\mathbf{L^f}$

Yield: 0.25 g, 0.10 mmol, 60 % 90.25 g (0.52 mmol) of diamine used).

^{1}H NMR (500 MHz, 298 K, CD_3OD): δ_H 9.28 (6H, s, HC=N), 7.75 (6H, t, $^3J_{HH}$ = 7.1 Hz, Py), 7.53 (6H, d, $^3J_{HH}$ = 7.1 Hz, Py), 7.36 (12H, d, $^3J_{HH}$ = 7.6 Hz, Ph), 7.23 (6H, t, $^3J_{HH}$ = 6.2 Hz, Py), 7.06 (6H, t, $^3J_{HH}$ = 7.0 Hz, Ph), 6.94 (12H, t, $^3J_{HH}$ = 7.1 Hz, Ph), 6.89 (12H, d, $^3J_{HH}$ = 7.6 Hz, Ph), 6.77 (6H, d, $^3J_{HH}$ = 5.0 Hz, Py), 6.69 (12H, d, $^3J_{HH}$ = 7.0 Hz, Ph), 5.96 (6H, d, $^3J_{HH}$ = 9.8 Hz, CH), 4.45 (6H, t, $^2J_{HH}$/$^3J_{HH}$ = 10.9 Hz, OCH_2CH), 4.37 (6H, d, $^2J_{HH}$ = 11.5 Hz, OCH_2Ph), 3.86 (6H, s, PhCH_2Ph), 3.23 (6H, d, $^2J_{HH}$ = 9.8 Hz, OCH_2CH).

$^{13}C\{^1H\}$ NMR (125 MHz, 298 K, CD_3OD): δ_C 172.8 (C=N), 160.4 (Ar), 154.5 (Ar), 141.9 (Ar), 139.8 (Ar), 136.6 (Ar), 136.4 (Ar), 130.4 (Ar), 130.2 (Ar), 129.3 (Ar), 128.8 (Ar), 127.1 (Ar), 74.2 (O*C*H_2Ph), 73.9 (O*C*H_2CH), 72.9 (CH), 41.5 (Ph*C*H_2Ph).

MS (ESI): m/z 511.4 $[Fe_2\mathbf{L^g}_3]^{4+}$.

FTIR: ν cm^{-1} 3360 br, 3043 w, 2915 w, 2858 m, 1612 w, 1512 w, 1494 w, 1471 m, 1451 m, 1360 w, 1299 w, 1240 w, 1105 m, 1076 s, 1019 m, 1001 m, 757 s, 700 s.

Elemental analysis found (calculated for $C_{129}H_{124}Cl_4Fe_2N_{12}O_6\cdot 11H_2O$): % C 64.76 (64.83) H 5.50 (6.16) N 6.39 (7.03).

Δ_{Fe}-$[Fe_2L^g_3]Cl_4\cdot 11.5H_2O$ (Δ-5g)

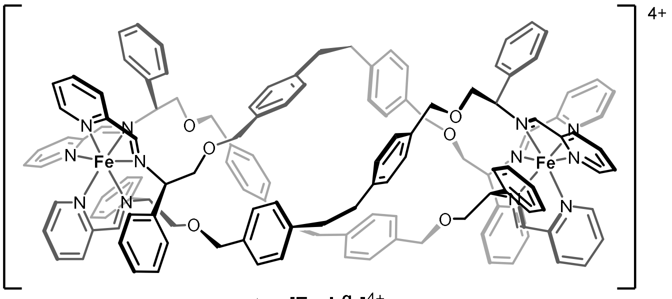

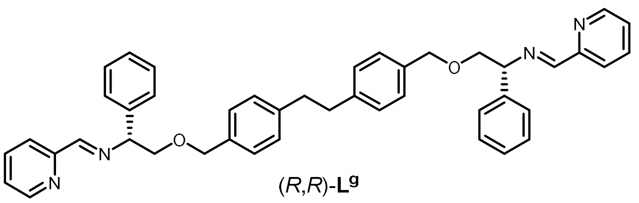

(*R*,*R*)-**L^g**

Δ_{Fe}-[Fe_2**L**$^g_3$]$^{4+}$

Yield: 0.32 g, 0.13 mmol, 77 % (0.25 g (0.52 mmol) of diamine used).

$^{13}C\{^1H\}$ NMR (125 MHz, 298 K, CD_3OD): δ_C 173.3 (C=N), 160.4 (Ar), 154.6 (Ar), 143.2 (Ar), 139.8 (Ar), 136.5 (Ar), 130.3 (Ar), 130.0 (Ar), 129.8 (Ar), 129.7 (Ar), 129.3 (Ar), 128.8 (Ar), 127.2 (Ar), 74.5 (O*C*H_2Ph), 73.3 (O*C*H_2CH), 72.7 (CH), 38.1 (CH_2CH_2).

MS (ESI): m/z 521.9 [Fe_2**L**$^h_3$]$^{4+}$.

FTIR: ν cm^{-1} 3372 br, 3023 w, 2921 w, 2855 m, 1612 w, 1589 w, 1513 w, 1494 w, 1471 m, 1451 m, 1385 1241 w, 1103 m, 1074 s, 1018 m, 1002 m, 815 w, 158 s, 700 s.

Elemental analysis found (calculated for $C_{132}H_{126}Cl_4Fe_2N_{12}O_6{\cdot}11.5H_2O$): % C 65.44 (65.05) H 5.87 (6.16) N 6.69 (6.90).

Λ_{Fe}-[$Fe_2L^g_3$]$Cl_4{\cdot}11.5H_2O$ (Λ-5g)

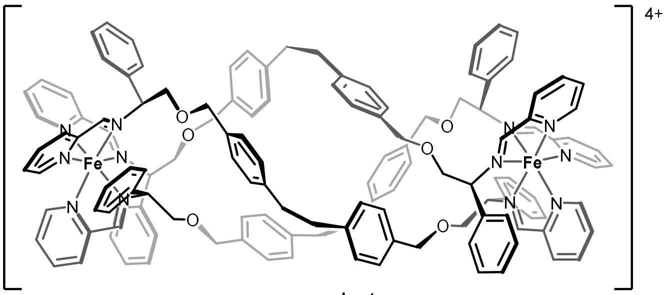

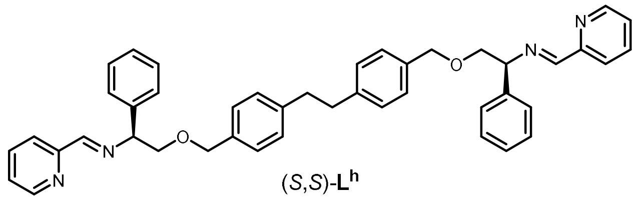

(*S*,*S*)-**L^h**

Λ_{Fe}-[Fe_2**L**$^h_3$]$^{4+}$

Yield: 0.32 g, 0.13 mmol, 78 % (0.25 g (0.52 mmol) of diamine used).

$^{13}C\{^1H\}$ NMR (125 MHz, 298 K, CD_3OD): δ_C 173.3 (C=N), 160.4 (Ar), 154.6 (Ar), 143.2 (Ar), 139.8 (Ar), 136.5 (Ar), 130.3 (Ar), 130.0 (Ar), 129.8 (Ar), 129.7 (Ar), 129.3 (Ar), 128.8 (Ar), 127.2 (Ar), 74.5 (O*C*H_2Ph), 73.3 (O*C*H_2CH), 72.7 (CH), 38.1 (CH_2CH_2).

MS (ESI): m/z 521.9 $[Fe_2\mathbf{L}^{\mathbf{h}}_3]^{4+}$.

FTIR: ν cm^{-1} 3372 br, 3023 w, 2921 w, 2855 m, 1612 w, 1589 w, 1513 w, 1494 w, 1471 m, 1451 m, 1385 1241 w, 1103 m, 1074 s, 1018 m, 1002 m, 815 w, 158 s, 700 s.

Elemental analysis found (calculated for $C_{132}H_{126}Cl_4Fe_2N_{12}O_6{\cdot}11.5H_2O$): % C 64.48 (65.05) H 5.66 (6.16) N 6.69 (6.90).

Δ_{Fe}-$[Fe_2\mathbf{L}^{\mathbf{h}}_3]Cl_4{\cdot}13H_2O$ (Δ-5h)

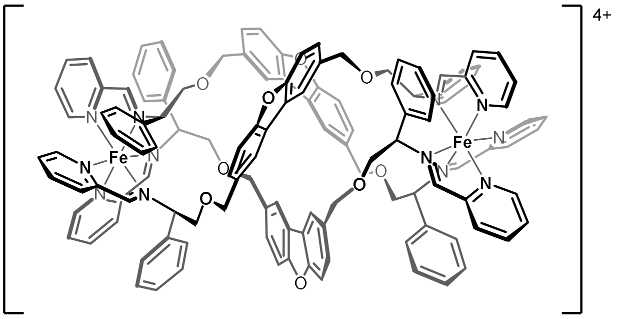

Δ_{Fe}-$[Fe_2\mathbf{L}^{\mathbf{h}}_3]^{4+}$

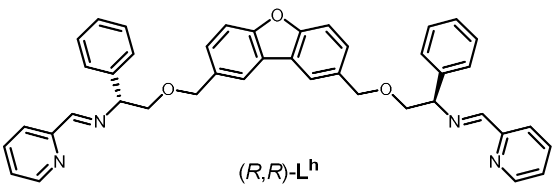

(*R*,*R*)-$\mathbf{L}^{\mathbf{h}}$

Yield: 0.38 g, 0.16 mmol, 88 % (0.25 g (0.54 mmol) of diamine used).

^1H NMR (500 MHz, 298 K, CD_3OD): δ_H 9.10 (6H, s, HC=N), 9.00 (6H, s, DBF), 7.76 (6H, d, $^3J_{HH}$ = 8.3 Hz, DBF), 7.69 (6H, t, $^3J_{HH}$ = 7.7 Hz, Py), 7.59 (6H, d, $^3J_{HH}$ = 8.3 Hz, DBF), 7.46 (6H, d, $^3J_{HH}$ = 7.7 Hz, Py), 7.14 (6H, t, $^3J_{HH}$ = 6.6 Hz, Py), 7.05 (6H, t, $^3J_{HH}$ = 7.4 Hz, Ph), 6.95 (12H, t, $^3J_{HH}$ = 7.4 Hz, Ph), 6.65 (6H, d, $^3J_{HH}$ = 5.6 Hz, Py), 6.50 (6H, d, $^3J_{HH}$ = 5.3 Hz, Ph), 5.55 (6H, dd, $^3J_{HH}$ = 10.8 Hz, $^3J_{HH}$ = 2.7 Hz, CH), 4.51 (6H, d, $^2J_{HH}$ = 9.2 Hz, OC*H*$_2$Ph), 4.02 (6H, t, $^2J_{HH}/^3J_{HH}$ = 11.0 Hz, OC*H*$_2$CH), 3.75 (6H, d, $^2J_{HH}$ = 9.2 Hz, OC*H*$_2$Ph), 2.34 (6H, dd, $^2J_{HH}$ = 10.8 Hz, $^3J_{HH}$ = 2.7 Hz, OC*H*$_2$CH).

$^{13}C\{^1H\}$ NMR (125 MHz, 298 K, CD_3OD): δ_C 172.7 (C=N), 160.4 (Ar), 158.2 (Ar), 154.3 (Ar), 139.7 (Ar), 135.8 (Ar), 133.6 (Ar), 131.7 (Ar), 130.3 (Ar), 130.1 (Ar), 129.4 (Ar), 128.6 (Ar), 125.0 (Ar), 123.2 (Ar), 113.1 (Ar), 74.8 (O*C*H_2Ph), 73.9 (O*C*H_2CH), 72.3 (CH).

MS (ESI): m/z 645.2 [$\mathbf{L^i}$+H]$^+$, 511.3 [$Fe_2\mathbf{L^i}_3$]$^{4+}$.

FTIR: ν cm^{-1} 3378 br, 3030 w, 2856 m, 1612 w, 1591 w, 1488 w, 1472 m, 1452 m, 1385 m, 1360 w, 1242 w, 1212 m, 1185 m, 1104 m, 1073 s, 1027 m, 1002 m, 830 w, 806 w, 758 s, 700s.

Elemental analysis found (calculated for $C_{126}H_{112}Cl_4Fe_2N_{12}O_9\cdot13H_2O$): % C 62.60 (62.38) H 5.13 (5.73) N 6.69 (6.93).

Λ_{Fe}-[$Fe_2L^h_3$]$Cl_4\cdot13H_2O$ (Λ-5h)

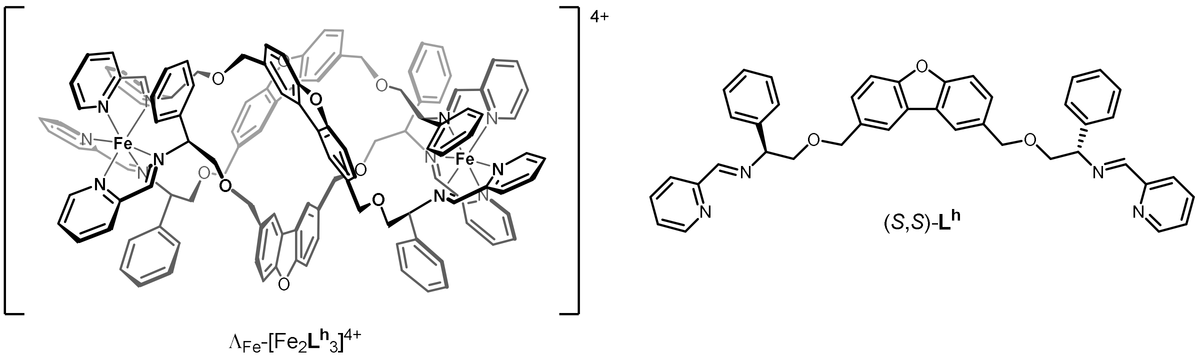

Yield: 0.36 g, 0.15 mmol, 83 % (0.25 g (0.54 mmol) of diamine used).

^1H NMR (500 MHz, 298 K, CD_3OD): δ_H 9.10 (6H, s, HC=N), 9.00 (6H, s, DBF), 7.76 (6H, d, $^3J_{HH}$ = 8.3 Hz, DBF), 7.69 (6H, t, $^3J_{HH}$ = 7.7 Hz, Py), 7.59 (6H, d, $^3J_{HH}$ = 8.3 Hz, DBF), 7.46 (6H, d, $^3J_{HH}$ = 7.7 Hz, Py), 7.14 (6H, t, $^3J_{HH}$ = 6.6 Hz, Py), 7.05 (6H, t, $^3J_{HH}$ = 7.4 Hz, Ph), 6.95 (12H, t, $^3J_{HH}$ = 7.4 Hz, Ph), 6.65 (6H, d, $^3J_{HH}$ = 5.6 Hz, Py), 6.50 (6H, d, $^3J_{HH}$ = 5.3 Hz, Ph), 5.55 (6H, dd, $^3J_{HH}$ = 10.8 Hz, $^3J_{HH}$ = 2.7 Hz, CH), 4.51 (6H, d, $^2J_{HH}$ = 9.2 Hz, OC*H*$_2$Ph), 4.02 (6H, t, $^2J_{HH}$/$^3J_{HH}$ = 11.0 Hz, OC*H*$_2$CH), 3.75 (6H, d, $^2J_{HH}$ = 9.2 Hz, OC*H*$_2$Ph), 2.34 (6H, dd, $^2J_{HH}$ = 10.8 Hz, $^3J_{HH}$ = 2.7 Hz, OC*H*$_2$CH).

$^{13}C\{^1H\}$ NMR (125 MHz, 298 K, CD_3OD): δ_C 172.7 (C=N), 160.4 (Ar), 158.2 (Ar), 154.3 (Ar), 139.7 (Ar), 135.8 (Ar), 133.6 (Ar), 131.7 (Ar), 130.3 (Ar), 130.1 (Ar), 129.4 (Ar), 128.6 (Ar), 125.0 (Ar), 123.2 (Ar), 113.1 (Ar), 74.8 (O*C*H_2Ph), 73.9 (O*C*H_2CH), 72.3 (CH).

MS (ESI): m/z 645.2 $[\mathbf{L^i}+H]^+$, 511.3 $[Fe_2\mathbf{L^i}_3]^{4+}$.

FTIR: ν cm^{-1} 3378 br, 3030 w, 2856 m, 1612 w, 1591 w, 1488 w, 1472 m, 1452 m, 1385 m, 1360 w, 1242 w, 1212 m, 1185 m, 1104 m, 1073 s, 1027 m, 1002 m, 830 w, 806 w, 758 s, 700s.

Elemental analysis found (calculated for $C_{126}H_{112}Cl_4Fe_2N_{12}O_9 \cdot 13H_2O$): % C 62.18 (62.38) H 4.95 (5.73) N 6.71 (6.93).

1.4 Optical purity of amines: synthesis and analysis of Mosher amides

The diamines (*R*/*S*)-**3a** and (*R*/*S*)-**3b** were synthesised using racemic phenylglycinol. These mixtures could not be distinguished from the nominally optically pure products *e.g.* (*R*,*R*)-**3a**, by NMR spectroscopy. Therefore a chiral derivatisation method was employed[4] whereby samples of nominally *S,S*-, *R,R*- and racemic (*i.e. S,S*/*R,R*/*S,R*) diamine were converted to the corresponding diamides using (*R*)-(+)-Mosher's acid (α-methoxy-α-trifluoromethyl-phenylacetic acid) and the products analysed by ^{1}H-NMR spectroscopy as follows.

Mosher's acid (90 mg, 0.38 mmol) was dissolved in anhydrous DCM (8 ml) and cooled to 0 °C. Oxalyl chloride (0.4 ml, 0.59 g, 4.7 mmol) was added, followed by 1 drop of DMF. After stirring for 1 hour the reaction mixture was concentrated *in vacuo* and the residue was suspended in hexane (10 ml) and concentrated *in vacuo* [^{19}F NMR (400 MHz, $CDCl_3$) δ_F -70.2]. The product was dissolved in anhydrous DCM (10 ml) to give a 38 mM solution of Mosher's acid chloride. The diamine (0.01 g, 20-30 µmol) was dissolved in DCM (5 ml) and 2.5 ml of the Mosher's acid chloride solution in DCM (95 µmol >3 eq.) was added, followed by saturated aqueous sodium carbonate (2 ml). After stirring overnight the phases are separated and the organic phase dried (magnesium sulfate), filtered and concentrated *in vacuo* to give crude Mosher's diamide. Kügelrohr distillation (120 °C, 20 min) was used to remove excess Mosher's acid and Mosher's acid chloride from the product, which was analysed without further purification.

The spectra for **3b** isomers are shown in Figure S1. The diamide derivative of the racemic diamine gave two resonances in the ratio 1:1 (green line) indicating again that the stereogenic centres are isolated as far as can be determined by ^{1}H-NMR spectroscopy at 400 MHz, but also that local diastereomeric units containing *S*- and *R*-amines could be distinguished *via* the ^{1}H chemical shift of the methoxy group in (*R*)-(+)-Mosher's acid. The diastereomeric diamides of (*R,R*)-**3b** (blue line) and (*S,S*)-**3b** (red line) gave essentially a single resonance for the OMe unit. Thus, the total enantiomeric excess of all amine-derived chiral centres could be measured for a given sample (found to be >98 % e.e.).

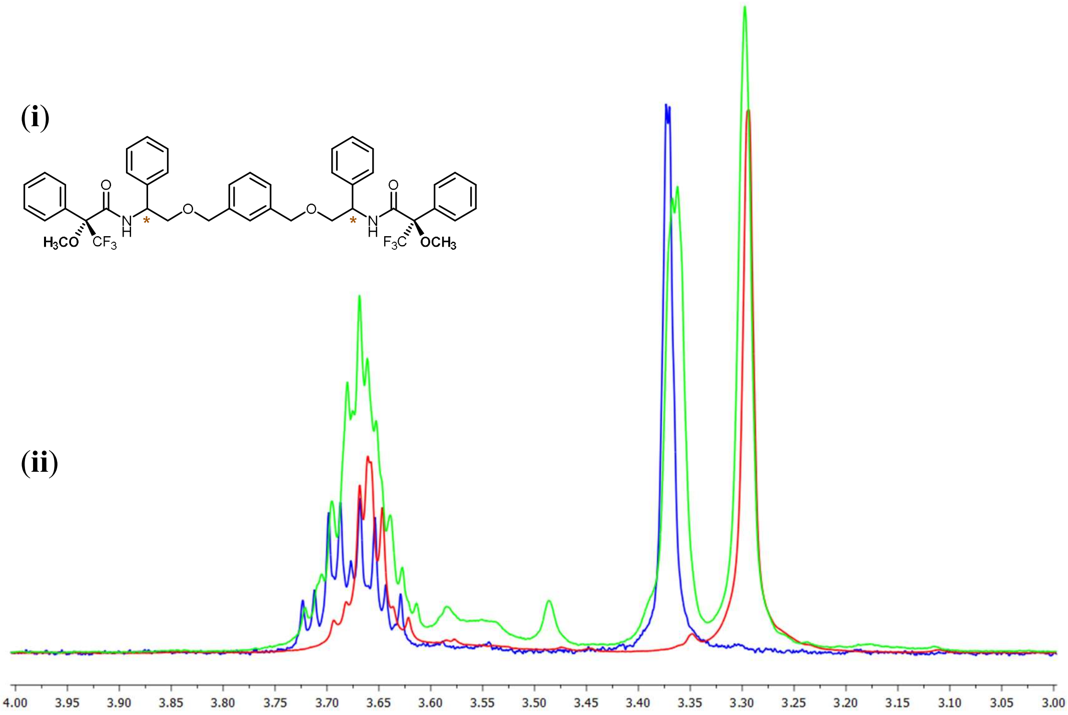

Fig. S1. NMR spectra of Mosher's amides (i) structure of **3b**-derived compounds with benzylic stereogenic centres labelled *. Overlaid 400 MHz ^{1}H-NMR spectra in $CDCl_3$ are shown for three samples: (ii) (*R*,*R*)-**3b** derivative in blue, (*S*,*S*)-**3b** derivative in red, isomeric mixture in green. Spectra were recorded at 298 K.

1.5 Electronic spectra

UV-Visible absorbance spectra (Fig. S2) were recorded using a Jasco V-660 spectrometer. Measurements were collected from a 1 cm path-length quartz cuvette and, unless otherwise mentioned, the standard parameters used were: bandwidth 1 nm, response time 1 sec, wavelength scan range 200-700 nm, data pitch 0.2 nm, scanning speed 200 nm/min, with four accumulation taken per sample to give an average spectrum with reduced noise.

Circular Dichroism CD spectra (Fig. S3) were measured on a Jasco J-815 spectrometer calibrated conventionally using 0.060% ACS a holmium filter. Measurements were collected using a 1 cm path-length quartz cuvette and unless otherwise mentioned the standard parameters used were: bandwidth 1 nm, response time 1 sec, wavelength scan range 200-700 nm, data pitch 0.2 nm, scanning speed 100 nm/min, with four accumulations.

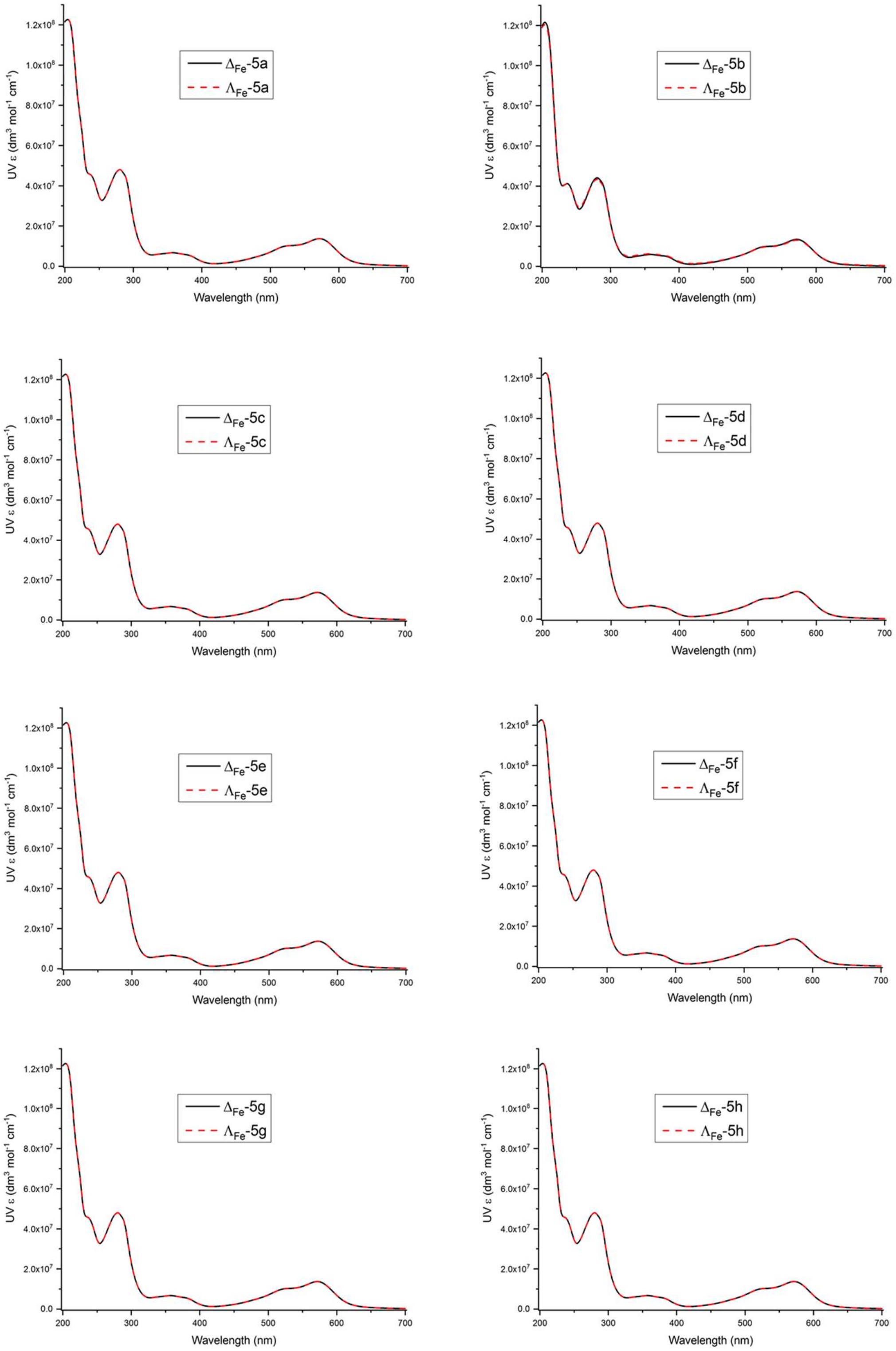

Fig. S2. UV spectra of enantiomers of metallohelices 5a-h (0.02 mM in water).

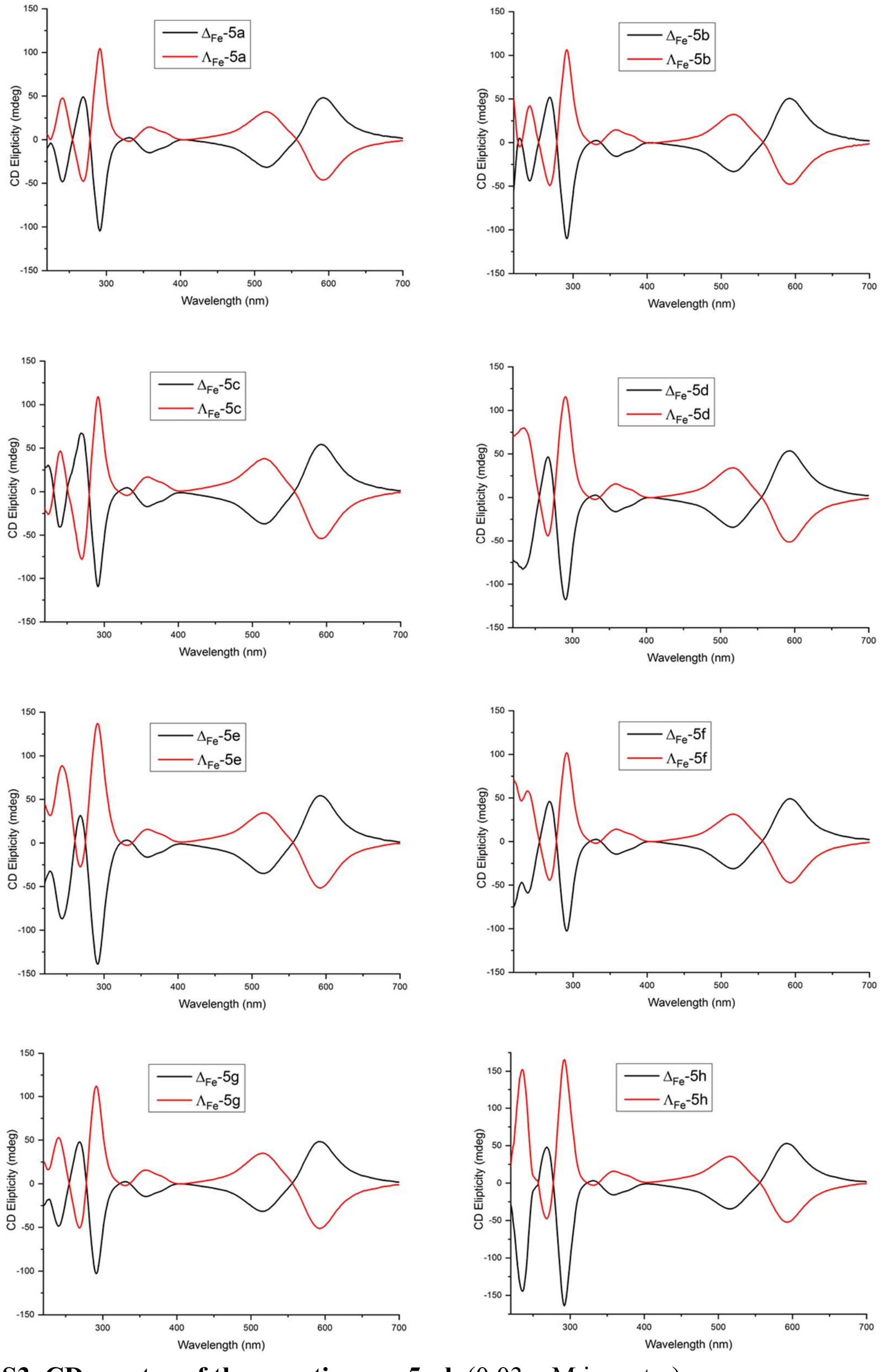

Fig. S3. CD spectra of the enantiomers 5a-h (0.03 mM in water).

1.6 Thermogravimetric analysis

Thermogravimetric analysis (TGA) was performed using a Mettler Toledo TGA/DSC 1 STAR® system instrument. Samples were weighed accurately into a pre-weighed 40 µl TGA/DSC aluminium crucible (DSC consumables Inc.) and heated 298 to 573 K (25 to 300 °C), at 5 K/min under a nitrogen atmosphere. The mass of the sample was recorded at various temperature points along this range. Mass decrease due to loss of water of crystallisation was correlated with microanalytical data.

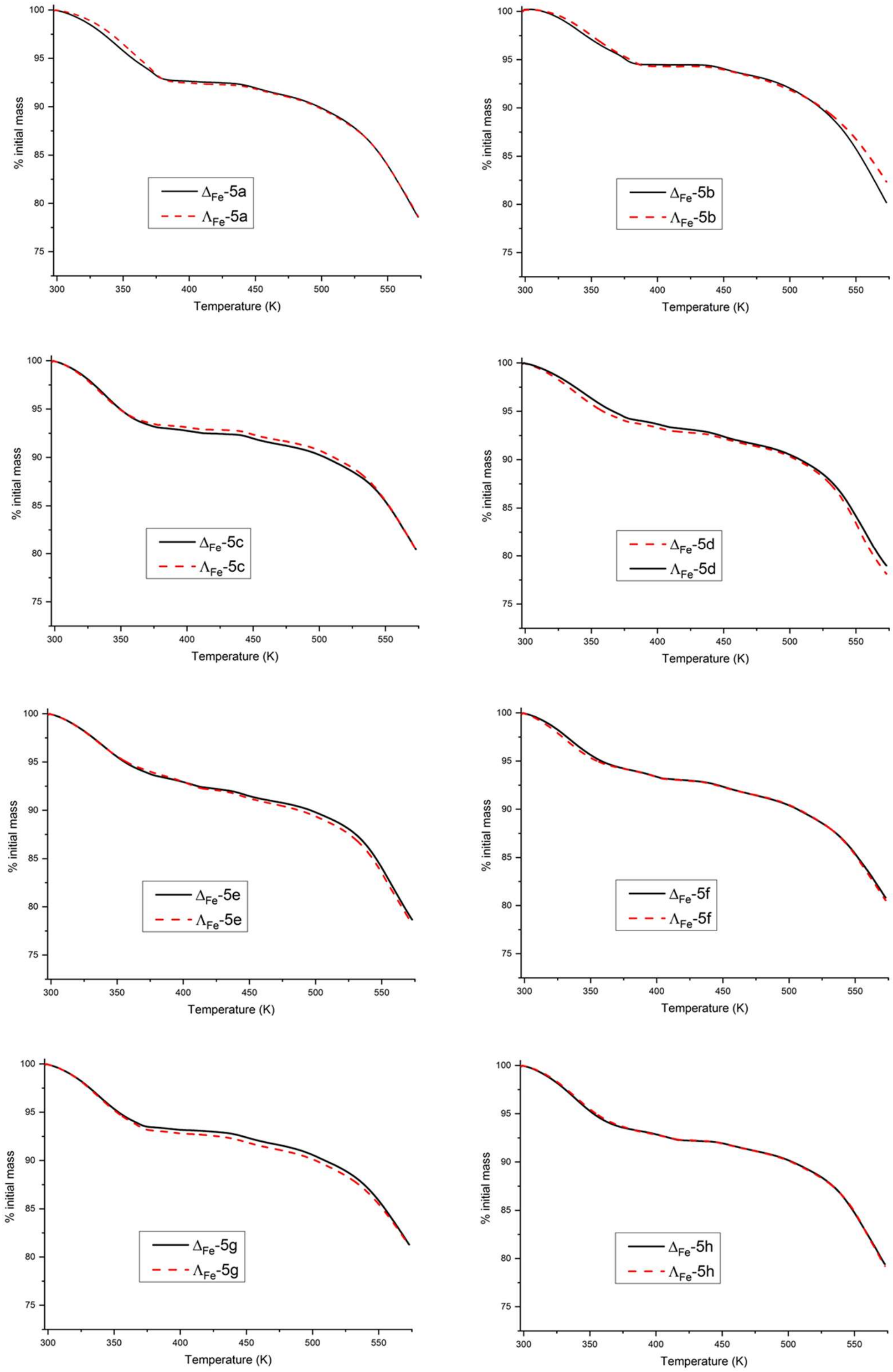

Fig. S4. Thermogravimetric analysis (TGA) of metallohelices 5a-h mass loss due to water of crystallization at lower temperatures was correlated with results of microanalysis.

1.7 Crystallography

Crystallographic data is uploaded to the Cambridge Crystallographic Data Centre (CCDC): 1904782, 1904783 and 1904784 with outline comments below. While the Zn(II) metallohelices with perchlorate counter-ions generally gave higher quality crystals, many challenges in refinement nevertheless resulted from disorder, twinning etc. In two instances, higher quality although still problematic crystals were grown by deliberately mixing the two enantiomers (as synthesised) in acetonitrile, then crystallising.

Compound 6b

Single crystals of $C_{112}H_{108}Cl_4N_{14}O_{22}Zn_2$ were grown from an optically pure sample in acetonitrile using a diffusion chamber with ethyl acetate. A suitable crystal was selected and mounted on a glass fibre with Fomblin oil and placed on an Xcalibur Gemini diffractometer with a Ruby CCD area detector. Using Olex2,[5] the structure was solved with the ShelXS[6] structure solution program using Direct Methods and refined with the ShelXL[7] refinement package using Least Squares minimisation.

Given the importance of this structure type, many crystals were investigated. Diffraction was weak and the data were recorded in the orthorhombic cell but refined finally in the lower symmetry monoclinic, and while this leads to a lower Friedel pair coverage the handedness of the sample was already known. The structure is twinned about the b axis swapping the a and c axis. The twin components refine to 0.5276(14). Since this is about a two-fold axis it does not affect the absolute configuration. The asymmetric unit contains the Zn helix, four ClO_4^- counter ions and two molecules of acetonitrile.

The structure was extensively disordered. Occupancy of the disordered components was originally linked to free variables and fixed at set values at the latter stages of the refinement. Xylenyl group C51 to C58 was modeled as disordered over two positions related by a small shift. The occupancy of the two components was fixed at 60:40. Similarly C87 to C94 was modeled over two positions at 50% occupancy. Perchlorates Cl30-O34 and Cl40-O44 where modeled over two positions related by a small shift in the anion. The occupancy of the two components of Cl30/Cl3A was fixed at 60:40. The occupancy of the two components of Cl40/Cl4A was fixed at 50:50. All atoms were refined anisotropically. Several DFIX, DANG and SIMU restraints were

used to give the disordered components reasonably bond lengths, angles and thermal parameters.

Crystal Data for $C_{112}H_{108}Cl_4N_{14}O_{22}Zn_2$ (*M* =2274.66 g/mol): monoclinic, space group $P2_1$ (no. 4), a = 12.3149(4) Å, b = 40.4691(4) Å, c = 12.3281(2) Å, β = 117.839(3)°, V = 5432.9(2) $Å^3$, Z = 2, T = 150(2) K, μ(CuKα) = 2.091 mm^{-1}, *Dcalc* = 1.390 g/cm^3, 19477 reflections measured (8.118° ≤ 2Θ ≤ 152.936°), 13837 unique (R_{int} = 0.0262, R_{sigma} = 0.0327) which were used in all calculations. The final R_1 was 0.0482 (I > 2σ(I)) and wR_2 was 0.1346 (all data).

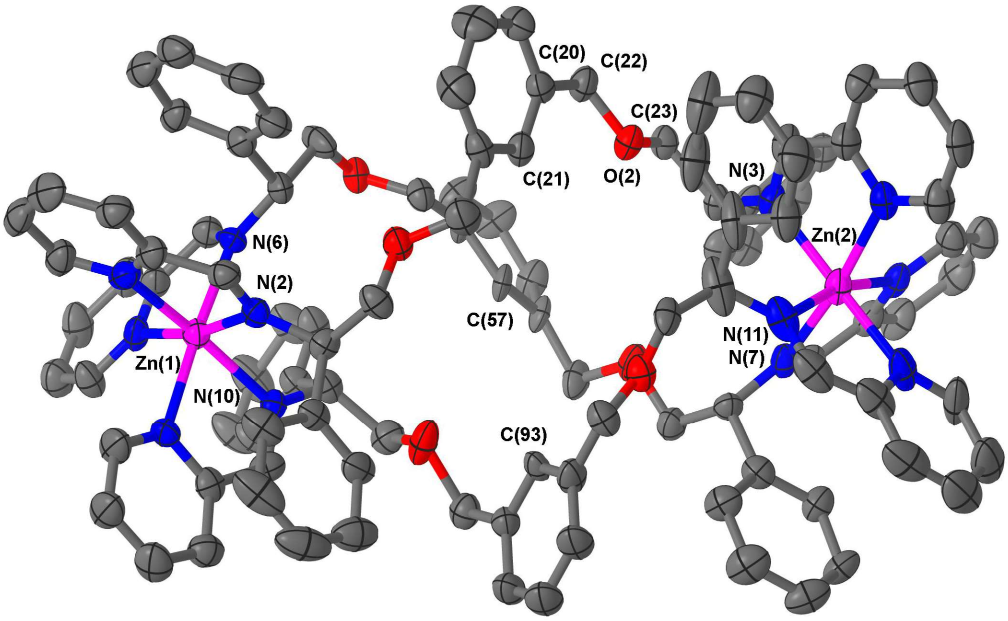

Fig. S5. Structure of the cation unit of **Δ-6b**. Ellipsoids modelled at 50% probability. Hydrogen atoms, solvent molecules and counterions removed for clarity. Zn(II) ions are shown in pink, nitrogen atoms in blue, oxygen atoms in red, and carbon atoms in dark grey.

Compound 6e

We are grateful to the EPSRC UK National Crystallography Service (NCS) for recording the data for this compound.[8]
Single crystals of C137.5H129.25Cl4N17.75O23S3Zn2 were grown from an acetonitrile solution of a deliberate mixture of the two enantiomers, layered with ethyl acetate. A suitable crystal was selected and mounted on a Mitegen head with Fomblin oil and placed on an AFC10 goniometer with a Rigaku FRE+ with VHF Varimax confocal mirrors and equipped with an HG Saturn 724+ CCD detector. The crystal was kept at 100(2) K during data collection. Using Olex2 [5], the structure was solved with the ShelXT [9] structure solution program using Direct Methods and refined with the ShelXL [7] refinement package using Least Squares minimisation.
The asymmetric unit contains the metallohelix, four perchlorates and seven molecules of acetonitrile. Acetonitrile molecules N1A-C2A, N1B-C2B andN1C-C2C were modelled at full occupancy. Acetonitrile N1D-C2D was modelled at 75% occupancy. Acetonitrile N1F-C2F and N1G-C2G were modelled at a half occupancy and refined iostropically. Another acetonitrile was modelled over two closely related positions (N1E-C2E and N1H-C2H) at 50% occupancy and refined isotropically. Perchlorate Cl20 was modelled as disordered over two positions about Cl20. The occupancies were linked to a free variable which refined to a ratio of 82:18. The minor component oxygen atoms were refined isotropically. Perchlorate Cl30 was modelled as disordered over two positions about Cl30. The occupancies were originally linked to a free variable but once this had settled were fixed at a ratio of 75:25 for the final stages of the refinement. The minor component oxygen atoms were refined isotropically. Perchorate Cl40 was modelled over two positions. The occupancy of these two perchlorate positions (Cl40 and l4A) was fixed at 50%. This disordered model was also composed of water molecules that shared that space when a perchlorate was not present. One perchlorate component Cl40 occupied the same location as water O1C (50% occupancy). The other perchlorate Cl4A occupied the same location as two closely related waters O1A (25% occupancy) and O1B (50%) occupancy. To clarify, one part of the disordered model has perchlorate Cl40 and waters O1A and O1B and the other part has perchlorate Cl4A and water O1B. No hydrogen atoms were located on these water molecules and the oxygen atoms were refined isotropically. Several DFIX, DANG and SIMU restraints were used to give the disordered components reasonably bond lengths, angles and thermal

parameters. Further, the structure contained large solvent accessible voids; the solvent masking algorithm in Olex2 was used to treat the electron density from solvent molecules that were not located as a diffuse contribution to the overall scattering without specific atom positions.

Crystal Data for C137.5H129.25Cl4N17.75O23S3Zn2 (M =2767.05 g/mol): monoclinic, space group C2/c (no. 15), a = 38.9929(5) Å, b = 22.5983(2) Å, c = 36.9118(6) Å, β = 104.4640(10)°, V = 31494.8(7) Å3, Z = 8,T = 100(2) K, μ(CuKα) = 1.904 mm-1, Dcalc = 1.167 g/cm3, 210313 reflections measured (4.556° ≤ 2Θ ≤ 134.798°), 28259 unique (Rint = 0.0614, Rsigma = 0.0286) which were used in all calculations. The final R1 was 0.0892 (I > 2σ(I)) and wR2 was 0.2800 (all data).

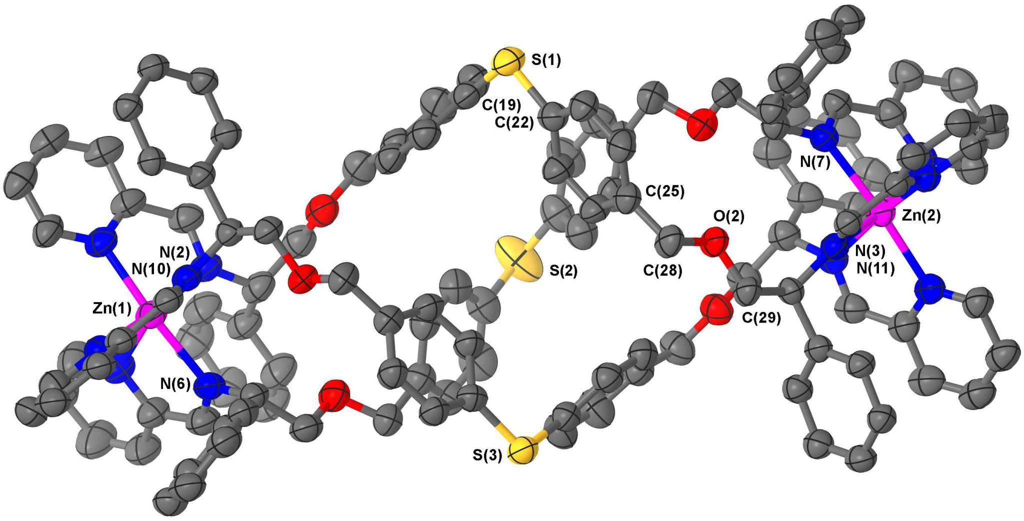

Fig. S6. Structure of the cation unit of **6e**. Ellipsoids modelled at 50% probability. Hydrogen atoms, solvent molecules and counterions removed for clarity. Zn(II) ions are shown in pink, nitrogen atoms in blue, oxygen atoms in red, sulfur atoms in yellow, and carbon atoms in dark grey.

Compound 6h

Single crystals of $C_{257}H_{223.5}Cl_6N_{26.5}O_{42}Zn_4$ were grown from acetonitrile solution of a deliberate mixture of the two enantiomers, layered with ethyl acetate. A suitable crystal was selected and mounted on a glass fibre with Fomblin oil and placed on an Xcalibur Gemini diffractometer with a Ruby CCD area detector. The crystal was kept at 100(2) K during data collection. Using Olex2 [5], the structure was solved with the ShelXS[6] structure solution program using Direct Methods and refined with the ShelXL [7] refinement package using Least Squares minimisation. The asymmetric unit contains two crystallographically independent metallohelices of opposite handedness. Six perchlorate anions and three acetonitrile molecules were located and refined but the other two perchlorates and additional solvent were not located (see below). Acetonitrile N900-C902 was refined at half occupancy. Several of the perchlorates and part of a ligand were modeled as disordered. The occupancy of the disordered components was originally assigned to a free variable but fixed for the final refinement cycles. Perchlorate Cl50 was modeled as disordered over two positions related by a rotation about the O51-Cl50 bond. The occupancies were fixed at 50:50. Perchlorate Cl70 was modeled as disordered over two positions related by a rotation about the O71-Cl70 bond. The occupancies were fixed at 50:50. Perchlorate Cl60 was modeled over two closely related positions. The occupancy was fixed at 70:30 Cl60-O64 to Cl6A to O64A.

The minor component oxygen atoms were refined isotropically. Phenyl C218-C223 was modeled as disordered over two positions related by a small displacement. The disorder was traced back to the benzylic carbon C217. The occupancy of the two components was fixed at 60:40 C217-C223 to C1A-C1G. Several DFIX, DANG and SIMU restraints were used to give the disordered components reasonably bond lengths, angles and thermal parameters. The solvent masking algorithm in OLEX2 was used to treat the electron density from those anions and solvent molecules that were not located as a diffuse contribution to the overall scattering without specific atom positions.

Weak data led to a B alert in the cifcheck. The fragile crystals were very weakly diffracting containing voids filled with disordered solvent. Crystals were measured at 150K with long exposure times but had little diffraction intensity out further than 165 degrees 2 Theta. This does not affect the structure determination.

Crystal Data for $C_{257}H_{223.5}Cl_6N_{26.5}O_{42}Zn_4$ (M =4829.29 g/mol): triclinic, space group P-1 (no. 2), a = 19.4539(5) Å, b = 23.9678(9) Å, c = 30.5016(15) Å, α = 90.746(3)°, β = 99.035(3)°, γ = 90.078(2)°, V = 14044.2(9) Å^3, Z = 2, T = 100(2) K, μ(CuKα) = 1.470 mm^{-1}, $Dcalc$ = 1.142 g/cm^3, 94201 reflections measured (4.68° ≤ 2Θ ≤ 133.192°), 48750 unique (R_{int} = 0.1177, R_{sigma} = 0.1930) which were used in all calculations. The final R_1 was 0.1034 (I > 2σ(I)) and wR_2 was 0.3181 (all data).

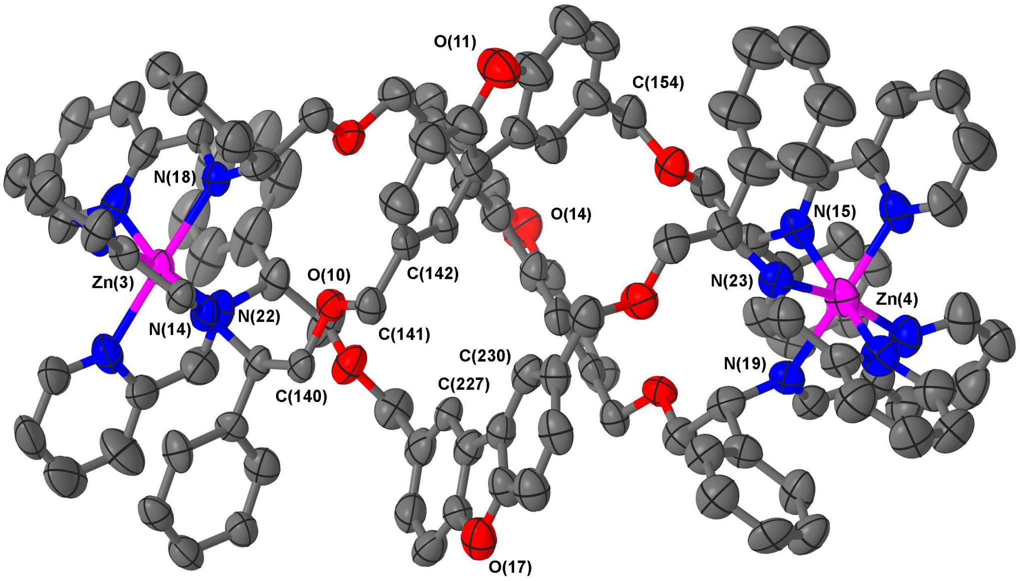

Fig. S7. Structure of the cation unit of **6h**. Ellipsoids modelled at 50% probability. Hydrogen atoms, solvent molecules and counterions removed for clarity. Zn(II) ions are shown in pink, nitrogen atoms in blue, oxygen atoms in red, and carbon atoms in dark grey.

Intermetallic distance

In Figure 2 of manuscript the horizontal axis has the metallohelices in order of increasing Fe-Fe distance. These were determined by crystallography, or where this was unavailable, by calculation (vide infra).

The crystallographic data comes exclusively from the Zn(II) structures, as described above. While there will be slight differences between the inner-sphere structures of Fe(II) and Zn(II) compounds, the size and shape of the metallohelix is determined by the nature and folding of the ligand strands.

Starting points for geometry optimisations were taken from adapted crystallographic structures. So as to capture possible conformational local minima, structures were optimised using ligand field molecular mechanics (LFMM)[10] as implemented in the DommiMOE program[11] before being annealed at 500 K for 1 ns prior to re-optimisation. Structures were further optimised using the Firefly quantum chemistry package[12] which is partially based on the GAMESS(US) source code[13] using B3LYP-D3(BJ)[14] functional and the 6-31g basis set with convergence criteria of 0.0001 a.u. For the compounds **5c** and **5g** a number of conformations were located.

Table S1. Intermetallic distance of metallohelices.

**Compound**	**Fe-Fe distance (Å)**	**Comments**
**5b**	12.4	From crystallography
**5a**	14.0	From crystallography
**5h**	14.4	From crystallography
**5d**	17.1	From molecular mechanics/DFT
**5f**	17.2	From molecular mechanics/DFT
**5e**	17.4	From crystallography
**5c**	>17.5	From molecular mechanics/DFT
**5g**	>17.5	From molecular mechanics/DFT

1.8 Stability testing

No decomposition was observed for 5b in aqueous solution over a period of weeks by NMR spectroscopy. In order to give an indication of stability, the compound was dissolved in KCl/HCl buffer (pH 1.5) in a UV-vis cell and the MLCT band at 550 nm was measured over time. While decomposition was still very slow, at 5 h ca 2% loss in signal was observed reproducibly (see figure). This corresponds to a first-order rate constant of 4.2×10^{-5} min^{-1}, or $t_{1/2} = 11$ d.

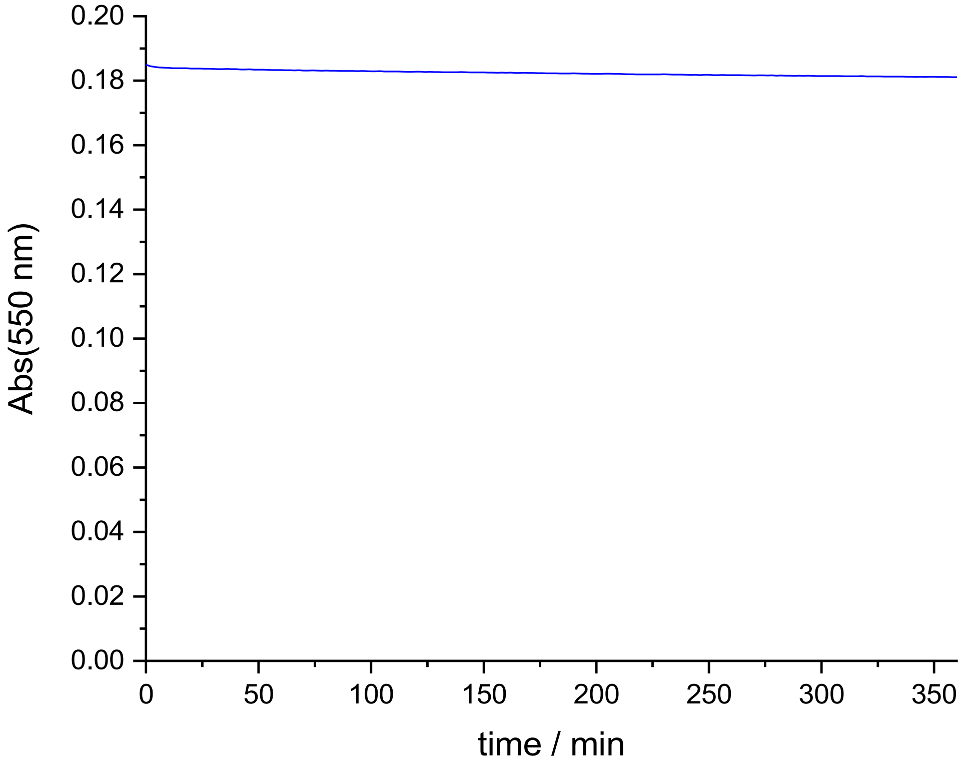

Fig. S8. Stability test. Single wavelength absorption measured (in MLCT band) of **Δ-5b** (0.02 mg mL-1). Aqueous KCl/HCl buffer, at pH 1.5.

2. DNA interaction assays

2.1 Ethidium bromide displacement

A Varian Cary Eclipse spectrofuorophotometer with a 1 cm quartz cell was used to perform fluorescence measurements at room temperature. Excitation of the CT-DNA- ethidium bromide complexes was performed at 546 nm and fluorescence emission was measured at 595 nm. Aliquots of 1mM metallohelix stock solutions were added to a solution of 1.3 μM ethidium bromide and 3.9 μM CT-DNA in 10 mM Tris buffer (pH 7.4) containing 1 mM EDTA, in a total volume of 2.5 mL. Fluorescence was measured after each addition until it was reduced to 50 %.

2.2 UV melting assays

A Varian Cary 4000 UV-Visible spectrophotometer, equipped with a Peltier controlled 6-sample cell-changer, was used to conduct the DNA stability assays. The absorbance at 260 nm was measured as a function of temperature using a heating rate of 0.4 ˚C/min, a 1 nm bandwidth and an average time of 2 s. The experiments were performed using masked 1.2 ml cuvettes of 1 cm pathlength. The T_m of CT-DNA was measured using a concentration of 7.5×10^{-5} M (per base) in 10 mM Tris buffer (pH 7.4).

2.3 Linear dichroism spectroscopy

A Jasco J-720 spectrometer adapted for LD spectroscopy was used to record flow LD spectra using a large volume (1 ml) Couette flow cell built by Crystal Precision Optics. The following parameters were used: 2 nm bandwidth, 0.25 s response time, wavelength scan range 200-800 nm, data pitch 0.5 nm, 500 nm/min scanning speed and accumulation 2. 0.2 mM CT-DNA was prepared in Tris buffer (10 mM, pH 7.4), and small aliquots of complex were added.

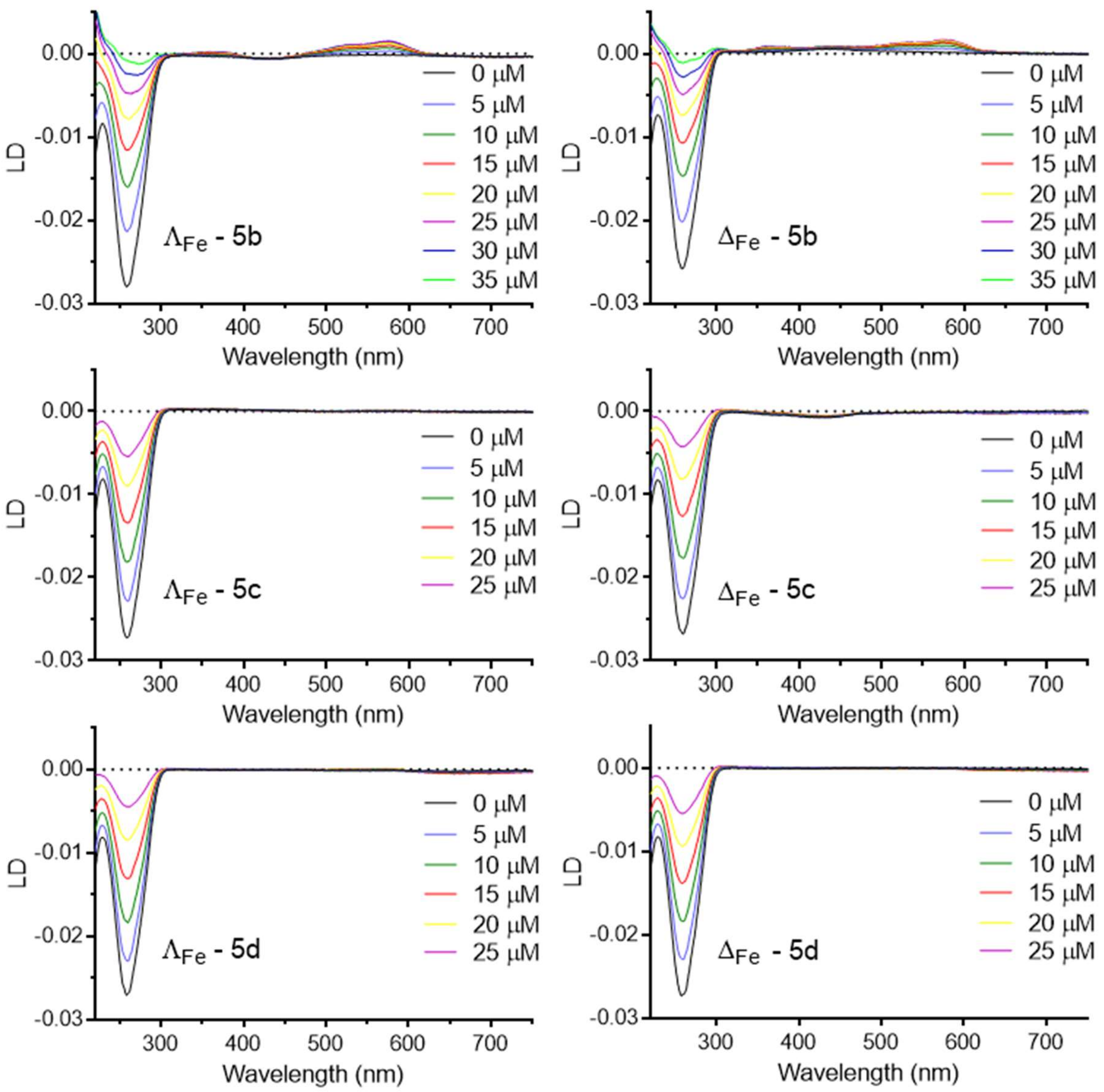

Fig. S9. Linear dichroism spectra of CT-DNA in the presence of metallohelices Λ-5b, **Δ-5b**, **Λ-5c**, **Δ-5c** and **Λ-5d** and **Δ-5d**. (Spectra in the presence of **Λ/Δ-5e-h** are similar to those of **Λ/Δ-5c-d**).

2.4 Atomic Force Microscopy

NdeI digestion was used to linearize plasmid pSP73, which was then purified using the Promega Wizard SV Gel clean-up kit. The compounds **Λ-5b** and **Δ-5b** were dissolved in Milli-Q water and mixed with 20 ng of the plasmid at the required ratio in the presence of buffer containing 4 mM HEPES (pH 7.4), 5 mM KCl and 5 mM $MgCl_2$ and in a total volume of 10 µl. The mixture was incubated at room temperature for 10 min and 4 µl of the sample were then applied onto freshly cleaved mica (SPI, West Chester, PA). Following a 2 min incubation the mica was washed with MilliQ water (1 ml) and dried using compressed air. A Bruker MultiMode 8 atomic force microscope was used for imaging. Scanning was performed in ScanAsyst mode in air using ScanAsyst-Air probes from Bruker (Camarillo, CA, USA).

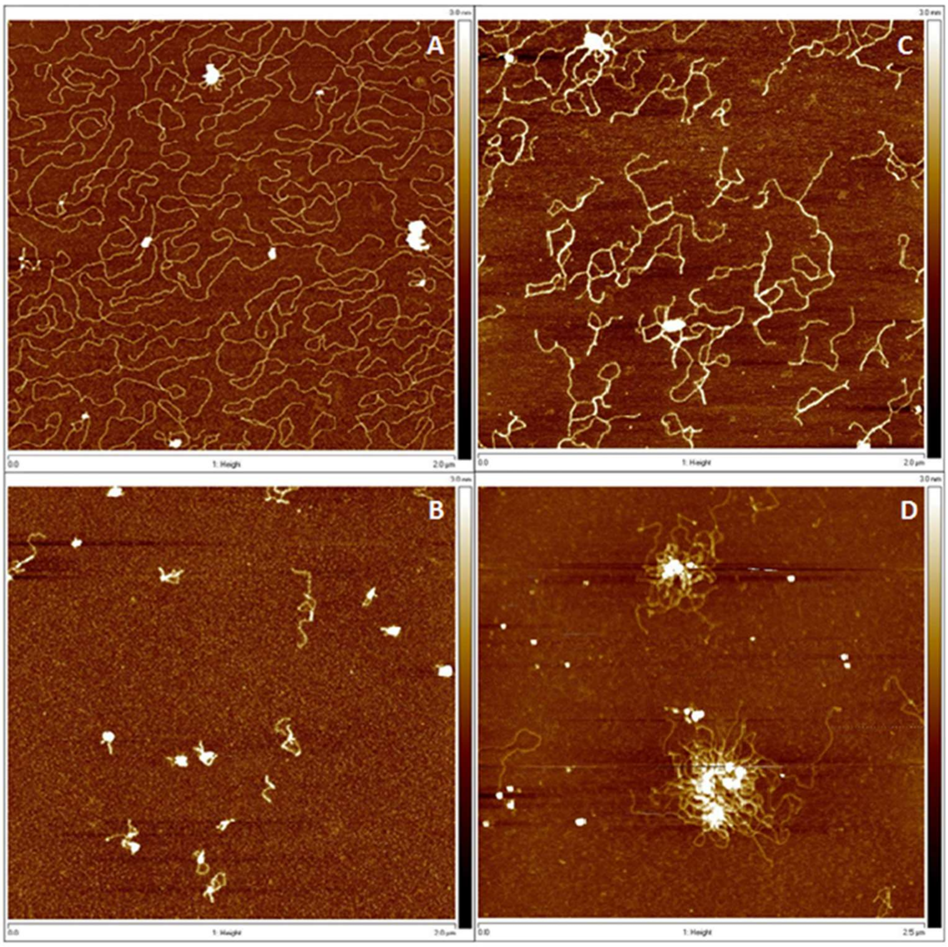

Fig. S10. AFM images of pSP73 linear plasmid DNA in the presence metallohelices **Λ/Δ-5b**. DNA mixed with **Λ-5b** at concentrations of 12.5 (A) and 25 μM (B), which correspond to DNA : metallohelix ratios 1:2 and 1:4, respectively, and in the presence of **Δ-5b** at concentrations of 25 (C) and 50 μM (D), which correspond to DNA : metallohelix ratios 1:4 and 1:8, respectively.

2.5 DNA Quadruplex binding FRET melting assay

The synthetic oligodeoxyribonucleotides were purchased from Eurofins Genomics (Ebersberg, Germany). The double-labelled (donor fluorophore FAM, 6-carboxyfluorescein; acceptor fluorophore TAMRA, 6-carboxytetramethylrhodamine) oligonucleotides:
F21T_HTelo, 5'-FAM-GGGTTAGGGTTAGGGTTAGGG-TAMRA-3';
F20T_c-kit, 5'-FAM-GGGCGGGCGCGAGGGAGGGG-TAMRA-3';
F21T_c-myc, 5'-FAM-GAGGGTGGGTAGGGTGGGTAA-TAMRA-3';
and F26T_ds-hairpin, 5'-FAM-CAATCGGATCGAATTCGATCCGATTG-TAMRA-3',
were annealed at a 4 µM concentration in 10 mM potassium phosphate buffer (pH 7) at 95 °C for 5 min and allowed to cool to room temperature overnight. DNA oligonucleotides at the concentration of 0.4 µM were mixed with 1.6 µM metallohelix in the absence of CT-DNA or the helicates were added at the concentration of 1.6 µM in the presence of 60 µM CT-DNA. Samples were prepared in 200 µL micro Eppendorf tubes in a total volume of 40 µL. Experiments with F21T_HTelo and F26T_ds-hairpin were performed at 40 mM K^+ concentration (additional 30 mM KCl). Measurements were performed on a real-time PCR instrument RotorGene 6000 (Corbett Research) with excitation at 470±10 nm and detection at 510±5 nm. Fluorescence readings were taken at intervals of 0.7 °C/min. The melting temperatures (T_m) were calculated within the RotorGene 6000 application program by applying a first derivative calculation.

2.6 Topoisomerase I and gyrase inhibition assays

For the enzyme inhibition assays, the compound **Λ-5b** was dissolved in 10 % DMSO at a concentration of 500 µM, and 3 µl of that were used in a total reaction volume of 30 µl, thus resulting in a final compound concentration of 50 µM. For the Topoisomerase I inhibition assay, supercoiled plasmid pBR322 was purified using the Qiagen Plasmid Maxi kit and 480 ng of plasmid were mixed with either 10 % DMSO or compound. The buffer used was 1 x Cutsmart buffer (NEB) and the *E. coli* enzyme was obtained from NEB (catalogue number M0301S). The enzyme was diluted in 1x Cutsmart buffer and 3 µl, corresponding to 1 U of enzyme, were added to the reaction mix. The reaction was allowed to proceed for 30 min at 37 ˚C. For the gyrase supercoiling assay, the enzyme and relaxed plasmid pBR322 were obtained from Inspiralis (catalogue number K0001). 500 ng of plasmid were again mixed with either compound or 10 %

DMSO and added to 1 x Inspiralis gyrase assay buffer. The enzyme was diluted 1/6 in dilution buffer and 5 µl of that were added to the reaction mix. The reaction was allowed to proceed for 30 min at 37 ˚C. The reactions were terminated by addition of 30 µl of loading buffer (40 % (w/v) sucrose, 100 mM Tris-HCl pH8, 10 mM EDTA, 0.5 mg/ml bromophenol blue), followed by 30 µl of chloroform-isoamyl alcohol (v/v, 24:1). The mixtures were shaken and the organic and aqueous phases were separated by centrifugation. The upper phase was then transferred to a 1 % agarose gel in TAE buffer. The gels were run at 85 V for 1 h 50 min, stained with SybrSafe and visualised using a GeneSys Syngene gel imager.

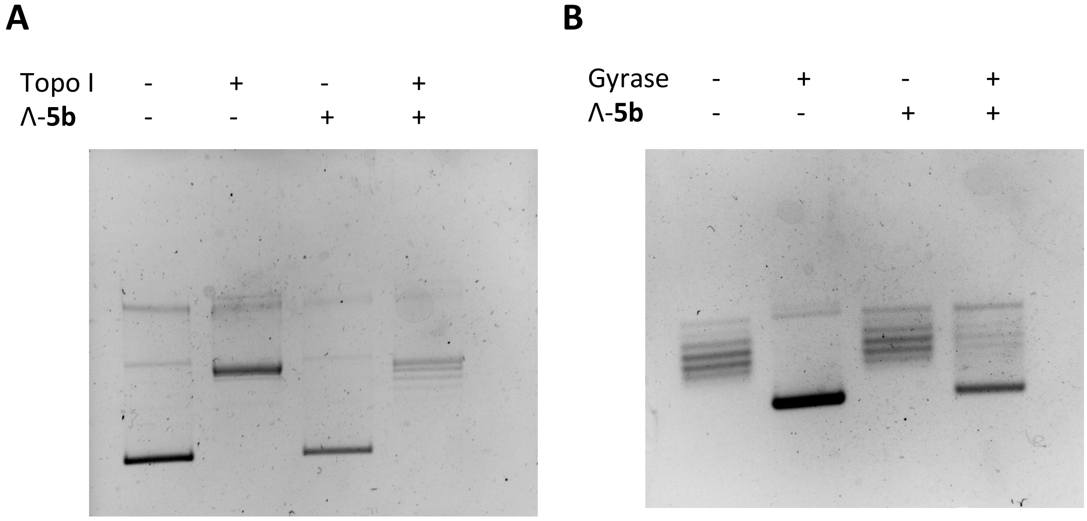

Fig. S11 Inhibition of plasmid pBR322. A) relaxation by topoisomerase I and B) supercoiling by gyrase in the presence of **Λ-5b**. Compound **Λ-5b** was dissolved in 10 % DMSO at a concentration of 500 µM, and 3 µl of that were used in a total reaction volume of 30 µl, thus resulting in a final compound concentration of 50 µM.

3. Microbiology methods

3.1 Determination of minimum inhibitory concentrations (MIC) and minimum bactericidal concentrations (MBC)

Table S2. Bacterial strains used in this study

Strain	Other characteristics	Source
B. subtilis 168		ATCC 6051 (Dowson laboratory – University of Warwick)
S. aureus USA300	MRSA	ATCC BAA-1717
E. coli MG1655	K12 strain	ATCC 700926
E. coli TOP10	DH10β strain	ATCC PTA-10989 (Invitrogen)
E. coli CFT073	UPEC O6:H1:K2	ATCC 700928 (Constantinidou laboratory - University of Warwick)
E. coli EDL933	EHEC O157:H7 Δ*stx*1-2 (derived from ATCC 700927)	Constantinidou laboratory
E. coli Sakai	EHEC O157:H7 Δ*stx*1-2 (derived from ATCC BAA-460)	Constantinidou laboratory
K. pneumonia K6	Reference strain, SHV-18 positive	ATCC 700603
K. pneumonia KP02	OXA-48 positive isolate	NCTC 13442
E. cloacae 684	AmpC positive isolate	NCTC 13405
A. baumannii NCTC 13420	Reference MDR strain	NCTC 13420
P. aeruginosa PAO1	Legacy strain	ATCC 15692

Table S3. *In vitro* antimicrobial activity (MICs) of metallohelices and controls against *B. subtilis 168*.

Group	Compound	MIC/ µg/ml (µM)
Metallohelices	Λ-**5a**	**2** (1.0)
	Δ-**5a**	**1** (0.5)
	Λ-**5b**	**4** (2.0)
	Δ-**5b**	**1** (0.5)
	Λ-**5c**	**8** (3.4)
	Δ-**5c**	**8** (3.4)
	Λ-**5d**	**1** (0.4)
	Δ-**5d**	**2** (0.8)
	Λ-**5e**	**4** (1.6)
	Δ-**5e**	**8** (3.3)
	Λ-**5f**	**8** (3.3)
	Δ-**5f**	**4** (1.7)
	Λ-**5g**	**8** (3.3)
	Δ-**5g**	**8** (3.3)
	Λ-**5h**	**2** (0.8)
	Δ-**5h**	**2** (0.8)
Antibiotics	Kanamycin	**0.5** (1.0)
	Tetracycline	**8** (18.0)

3.2 Λ-5b activity in stationary phase cells

Cultures of *E. coli* Sakai were grown overnight to stationary phase, collected by centrifugation, and re-suspended to an OD_{600} of 0.1, in 20 ml PBS containing either 8 µg/ml **Λ5b** or 20 % isopropanol before incubation for a further three hours at 37 °C. Cells were subsequently harvested by centrifugation and washed twice in clean sterile PBS. The final cell pellet was re-suspended to an OD_{600} of 0.01 in 50 ml CAMHB. These cultures were incubated at 37 °C and monitored for growth by taking periodic OD_{600} measurements and viability counts over five hours.

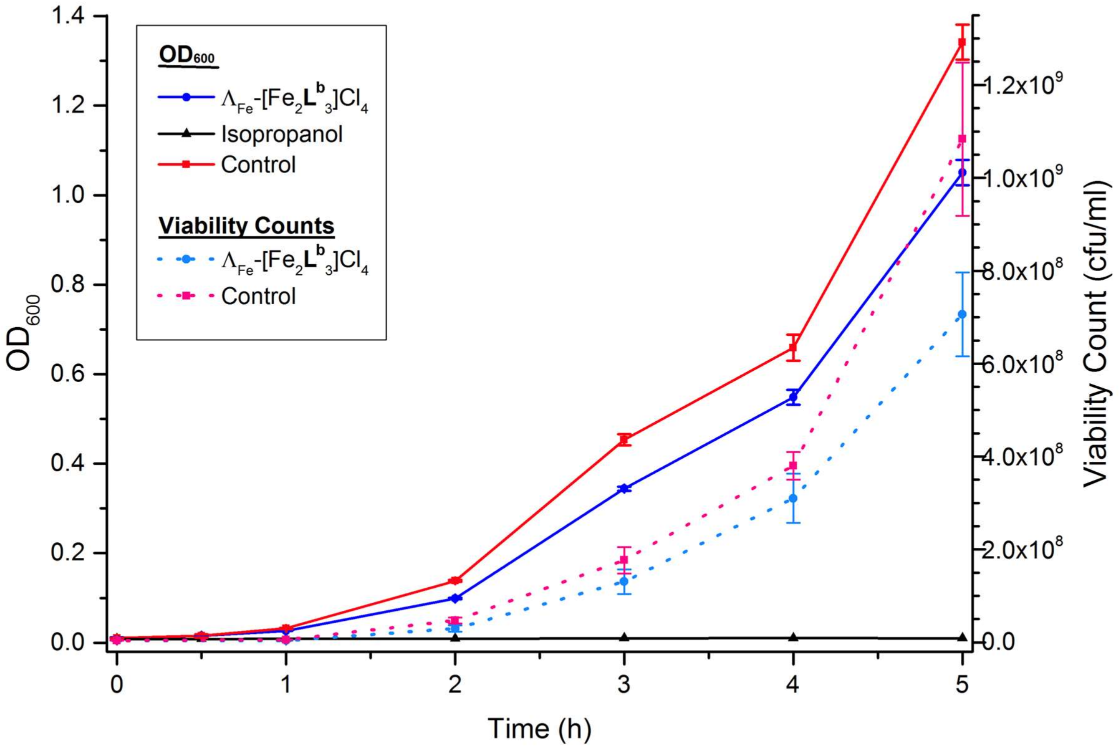

Fig. S12. Growth of *E. coli* O157:H7 Sakai in CAMHB after a 3 h exposure of stationary phase cells to Λ-5b at 4 × MIC. A control containing no compound, and severely lytic isopropanol (20 vol%) are included for comparison. OD600 measurements (solid lines) and viability counts (dotted lines) are shown. Error bars represent 95 % confidence intervals.

3.3 *BacLight*™ LIVE/DEAD assay – further details

Overnight cultures of the appropriate bacterial strain in CAMHB were divided into two and cells collected by centrifugation. One pellet was resuspended in fresh saline solution (10 ml of 0.85 % NaCl by weight), the other in 70 % aqueous isopropanol. Both suspensions were incubated, with gentle shaking, for 1 h at room temperature, before harvesting by centrifugation. Pellets were resuspended in fresh saline solution to yield a cell suspension of OD_{600} = 0.2. Metallohelix solutions of 10× the desired final concentration were prepared in saline containing 10 % methanol, and they were added to the live (intact) cell suspension at a ratio of 9:1 (cells:metallohelix). These were distributed into separate wells of a sterile Falcon™ 96-well plate (100 µl each). Additionally, different proportions of live and dead cells were mixed to obtain cell suspensions containing different proportions of live/intact cells (100 %, 90 %, 50 %, 10 % and 0 %). Aliquots of these mixtures (100 µl) were applied to each plate as controls. Plates were incubated for 40 min at 37 °C, then 100 µL of BacLight™ LIVE/DEAD dye solution (prepared according to the manufacturer's instructions) was added. After 15 min incubation in a darkroom at ambient temperature, fluorescence output of the samples was measured using a VarioSkan Flash (ThermoFisher) plate reader, set to measure the output at wavelength 530 nm and 630 nm respectively (12 nm bandwidth, 100 ms measurement time), from an excitation at wavelength 485 nm. A delay of 200 ms was included between measurements. Cell membrane integrity was quantitatively determined using the controls to plot a standard curve. Experimental samples were repeated five times, and controls were repeated in triplicate.

Table S4. Percentage membrane integrity of *E. coli* exposed to metallohelices 5b for 45 min, either at the corresponding MIC or at 4 × MIC. Errors represent 95 % confidence intervals. Absolute concentrations in µg/ml are included in parentheses. The value obtained for ampicillin at 4 µg/ml is included for reference.

	E. coli TOP10	
**Compound**	**1 × MIC**	**4 × MIC**
Λ-**5b**	**100.5** ± 8.9 (2 µg/ml)	**96.7** ± 5.7 (8 µg/ml)
Δ-**5b**	**99.1** ± 8.3 (4 µg/ml)	**95.7** ± 3.2 (16 µg/ml)
Ampicillin	**93.2%** ± 6.2 (4 µg/ml)	

3.4 Isolation of tolerant mutants – further details

Overnight cultures of 7 independent biological replicates of E. coli Sakai (serving as parent lines a-f) were grown in CAMHB, and 25 µl of each culture was applied to MH agar containing 80 µg/ml of **Λ-5b** (a high concentration was used here as the compound does not appear to diffuse well in solid media) and incubated at 37 ˚C overnight. Additionally, the cultures were serially diluted to obtain viable counts which were calculated to be 1.78×10^9 cfu/ml on average ($\sigma = 0.13 \times 10^9$ cfu/ml). An average of 11.1 ($\sigma = 3.3$) colonies were observed, which would suggest a positively selected mutation frequency of 2.49×10^{-7} ($\sigma = 0.78 \times 10^{-7}$) under these conditions, assuming all colonies represented genuine resistant clones. Four colonies from each replicate were then re-streaked onto MH agar both with and without supplementation with the lead compound. These were used to seed fresh cultures in MH broth to a cell density of 5×10^5 cfu/ml, whereupon the lead compound was added to a final concentration of 2 µg/ml, representing the Wild Type (WT) MIC. Growth of these cultures was then monitored (OD_{600}) for 20 h at 37 °C. Only 17 of the isolates were able to grow to an $OD_{600} > 0.5$ under these selective conditions, with at least one isolate arising from each of the seven original parent line. The MIC of the **Λ-5b** for the isolates was measured as described above and the bacteria were studied by whole-genome sequencing.

Table S5. Characterisation of EHEC Sakai tolerant mutants. Shown is the MIC of the lead compound for each isolate as well as genomic changes identified via EnteroBase. Relative plasmid (pO157) levels calculated through comparing depths of coverage of the chromosome with that of the plasmid are also listed. Those identified later via PCR/Sanger sequencing are marked *.

**Class**	***E. coli* line**	**MIC** (µg/ml)	**Mutation type**	**Chromosome position** (NCBI reference)	**Change relative to WT**	**Gene(s) affected**	**Plasmid to Chromosome coverage ratio** (relative to WT)
	**WT**	**2**	**-**	**-**	**-**	**-**	**1**
I	a1	4	Deletion		550 bases lost	*waaG, waaQ*	1.53
	b2	4	Deletion	4550911	11 bases lost	*waaG*	0.56
II	b1	8	SNP	1736847	G to T		1.27
	c2	8	SNP	1737380	G to A		1.37
	e1	4	SNP	1737192	A to C	*galU*	1.07
	f1	8	SNP	1737192	A to C		0.67
III	a4	4	Deletion*	4971120	~1900 bases lost		0.01
	c3	4	SNP	4974478	C to G		0.03
	g1	4	Deletion	4974320	1 base (T) lost		0.56
	g2	4	Insertion	4973226	G inserted	*btuB*	0.43
	g3	8	Deletion*	4973227	1 base (G) lost		0.49
	g4	4	SNP	4974212	C to T		0.55
	d4	4					0.21
IV	e2	8		N/A			0.20
	e4	8					0.07
	f3	4					0.55
	f4	4					0.05

3.5 DNA extraction and whole genome sequencing

Bacteria to be sequenced were collected from 500 µl of overnight cultures in CAMHB and DNA was purified using a DNeasy® blood and tissue genomic DNA extraction kit (Qiagen). DNA concentrations were determined using the Qubit® high-sensitivity dsDNA quantification kit (ThermoFisher Scientific). Genomic DNA libraries were prepared using the Illumina Nextera® XT kit using 1ng of input DNA. Following PCR clean up, the DNA concentration of each library was measured using the Qubit® high-sensitivity dsDNA and the libraries were normalised to 4 nM and pooled together. The pool was prepared for loading following the Illumina Nexteria® XT guidelines. Sequencing was performed using a MiSeq Reagent Kit v2 (500-cycles) on an Illumina MiSeq™ instrument.

3.6 Extraction and purification of microbial RNA

EHEC Sakai was grown overnight and used to seed three 50 ml cation-adjusted MH broth subcultures. They were grown with shaking aeration at 37°C to mid-exponential phase (OD600 = 0.5) whereupon they were split into two paired samples. Into one of each of the paired samples we added a sub-MIC dose of metallohelix to a final concentration of 0.5 µg/ml. Into the other we added an equivalent volume of compound solvent only as a negative control. Paired samples were then returned to shaking incubation at 37°C for 40 min before harvesting the total RNA. Then, 1 ml of each sample was collected, added to 2 ml of RNAprotect reagent (Qiagen) and incubated for 5 min at room temperature. Enzymatic lysis was performed using TE buffer with proteinase K and 1 mg/ml lysozyme. RNA was then purified using the miRNeasy kit (Qiagen) with the inclusion of a double on-column DNA digestion. Absence of contaminating DNA was confirmed by PCR using primers for the E. coli 16S ribosomal subunit gene (forward: AGAGTTTGATCMTGGCTCAG, reverse: GGTTACCTTGTTACGACTT). RNA integrity was verified using the RNA 6000 pico kit (Agilent) on the Agilent 2100 Bioanalyzer. To deplete rRNA, The Ribo-Zero™ bacterial rRNA removal kit (Illumina) was used according to the manufacturer's instructions with an input RNA of 4 µg per sample. Elution of the depleted RNA was performed using 5.5 µl of water and 0.5 µl of that were again run on the Agilent 2100 Bioanalyzer to confirm rRNA depletion.

3.7 Generation of cDNA Libraries and sequencing

The remaining 5 µl of rRNA-depleted sample were used as input for cDNA library preparation using the Illumina TruSeq^{TM} stranded mRNA kit with a slightly modified library preparation protocol. Specifically, the 5 µl of input RNA were directly mixed with 13 µl of TruSeq^{TM} 'Fragment, Prime, Finish mix' and incubated at 94 °C for 8 min. Then, 17 µl of the reaction mix were transferred to a new tube for cDNA synthesis and the TruSeq Stranded mRNA sample preparation guidelines were followed from this point onwards. cDNA libraries were quantified using the Qubit® High Sensitivity DNA assay kit and fragment sizes were determined using the High Sensitivity DNA kit on the Agilent 2100 Bioanalyzer. The libraries were normalised to 4 nM and pooled together. Paired-end sequencing was performed using two Miseq reagent kits v3 (150-cycle) on an Illumina MiSeq^{TM} sequencer.

3.8 Bacterial sub-cellular compound localization- further details

For visualisation of the sub-cellular localisation of the target compound we used the Click-IT cell reaction buffer kit (Invitrogen) as per the manufacturer's instructions and following the procedure described below. To observe the dividing cells, overnight culture of EHEC Sakai Δ*stx 1-2* was diluted in cation-adjusted Mueller Hinton broth and grown to mid-exponential (OD600 = 0.52). Then, **Λ-5b'**, at the MIC of 8 µg/ml or at a quarter of the MIC (2 µg/ml), or methanol were added to the culture and incubated for 30 min at 37 ˚C. Ten minutes before the end of the incubation period, 5 µg/ml of the membrane dye FM™ 4-64FX (Invitrogen) was added to stain the cell membrane. Then, the cells (1 ml for each treatment) were collected by centrifugation and fixed with 4 % PFA for 15 min at 4 ˚C. They were then washed with PBS by centrifugation and permeabilised with 0.5 % Triton X-100 in PBS by incubating at room temperature for 30 min. Cells were washed with PBS followed by 2 % BSA by centrifugation at 5000 g and resuspended in 180 µl of the Click reaction mix (containing 5 µM AF-488 azide). They were incubated at room temperature in the dark for 30 min, washed with 2% BSA in PBS and stained with 1 µg/ml DAPI for 1 min. They were finally washed with PBS and used for microscopy. For stationary

phase cells, the same procedure was followed with the following modifications. A 24 h culture of EHEC Sakai Δ*stx 1-2* at an OD600 of 2.9 was used. Cells were treated with the compound only at the MIC concentration of 8 µg/ml, or methanol. Following incubation with the membrane dye, 700 µl of the culture were collected (an OD600 equivalent of 2.0) and 360 µl of the Click reaction mix were used instead, to account for the higher number of bacteria present. Slides were prepared using either 1 % agarose pads (in PBS) or Poly-L-lysine (0.01 % solution) solution. Samples (3 µl) were then immobilised onto the slides, allowed to dry and then 4 µl *SlowFade* Gold antifade reagent (ThermoFisher Scientific) were added and coverslips were mounted for viewing.

Microscopy

Imaging was performed on a Leica DMi8 Inverted Microscope equipped with a Hamamatsu Orca Flash 4.0 v2 CMOS camera and an EL6000 mercury metal halide external light source. The filters used were DAPI (Excitation: 350/50, Emission: 460/50), FITC (Excitation: 480/40, Emission: 527/30) and TXR (Excitation: 560/40, Emission: 630/75). Exposure and intensities were kept the same for all the samples (DAPI: 510.348 ms, FITC: 1553.223 ms, TRX: 410.038 ms). The HC PL FLUOTAR 100x/1.32 oil PH3 objective was used throughout.

Image analysis

Fluorescence microscopy images were analysed using ImageJ/Fiji. For quantitation, individual bacterial cells were selected using the red channel images showing the membrane stain. In brief, the red channel images were processed using the Filter → Convolve command with the default kernel and an automatic median threshold was applied. Then, cells were automatically detected using particle analysis with a specified size range of 0.5-6.0. The regions of interest (ROIs) were manually confirmed and those that did not correspond to individual cells were discarded. These ROIs were then used on the green and blue channels to measure the corresponding intensities per bacterial cell. Based on a histogram of the mean blue intensity values, the cells in the sample treated with the lead compound were split into two populations (of DAPI mean intensity/cell either above or below 11000). A Kolmogorov-Smirnov test was then performed to evaluate if the

mean AF-488 (green) intensity values of these populations were significantly different from each other.

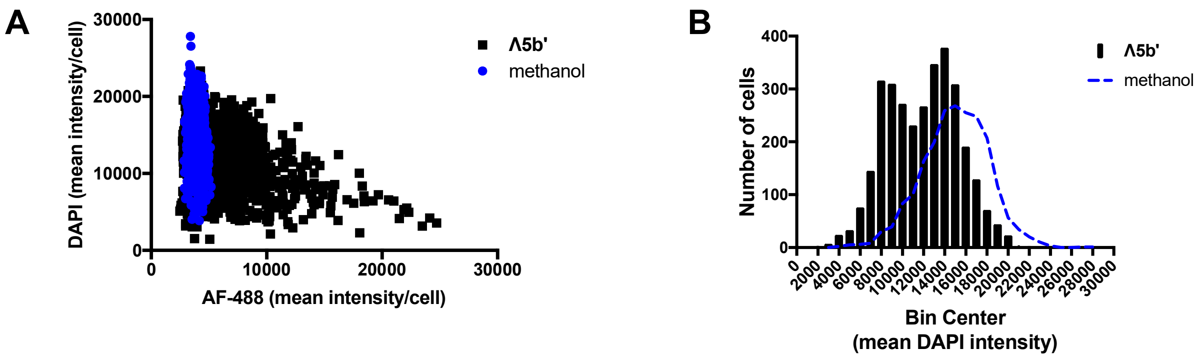

Fig. S13. Quantification of DAPI and AF-488 intensities following bacterial labelling assay. Exponentially growing EHEC Sakai cells were treated with either 8 µg/ml **Λ-5b'** or methanol and stained for membrane (FM 4-64 FX), nucleic acid (DAPI) and the **Λ-5b'** (via click reaction with AF-488 azide). A) Mean intensity values per cell in bacteria treated with either **Λ-5b'** or methanol for DAPI (blue channel) and AF-488 (green channel) fluorescence. B) Histogram analysis of the mean DAPI intensity/cell showing a bimodal distribution in the **Λ-5b'** treated sample compared to the near normal distribution of the methanol treated sample.

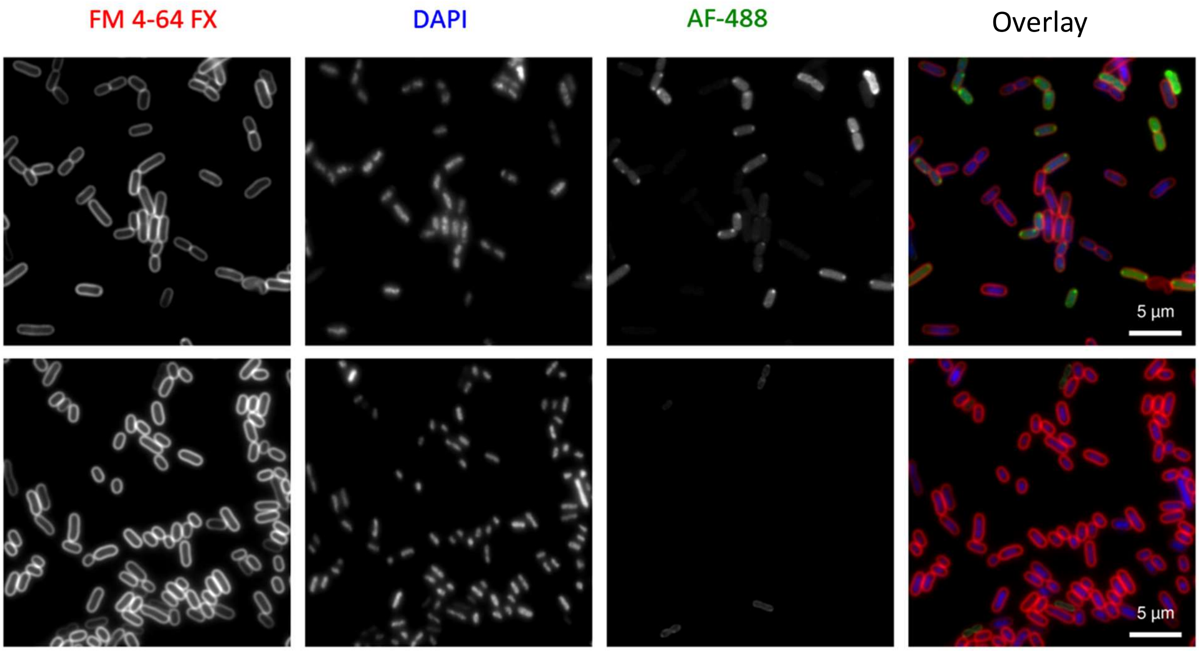

Fig. S14. Cells in exponential phase (upper) or stationary phase (lower) were treated with 8 µg/ml **Λ-5b'** and stained for membrane (FM 4-64 FX), nucleic acid (DAPI) and the **Λ-5b'** (via click reaction with AF-488 azide). Note that in stationary phase, only cells lacking membrane staining showed weak **Λ-5b'** staining.

4. Bioinformatics

4.1 Whole genome analysis

Fastq reads from the MiSeq whole genome sequencing of WT and tolerant clones were uploaded to EnteroBase[15] (http://enterobase.warwick.ac.uk) and assembled into contigs. The genomes of the tolerant clones were then aligned against the WT assembly whilst all genomes including the WT were also aligned against the reference E. coli O157:H7 Sakai (Assembly: GCF_000008865.1) for SNP and insertion/deletion (indel) detection. The EnteroBase analysis containing the assembled genomes and the two SNP projects is available online and can be accessed at http://enterobase.warwick.ac.uk/species/ecoli/search_strains?query=workspace:4462, while raw reads have been deposited to NCBI under project PRJNA517036. In addition, for confirmation of the EnteroBase analysis, alignment of raw reads to the reference E. coli O157:H7 Sakai genome (Assembly: GCF_000008865.1) including plasmids pO157 and pOSAK1 (Accession numbers: NC_002695 for the chromosome, NC_002128 for pOSAK1 and NC_002127 for pO157) was performed using Bowtie2.[16] For the purposes of mapping, the chromosome and two plasmids were used as reference in a single multifasta file. Bam files were sorted and indexed using Samtools[17] and visualised in Artemis genome browser[18] for manual validation. Qualimap[19] was used to calculate mapping statistics. Finally, SNP and small indel detection was also performed using VarScan2.[20] To confirm the mutations by PCR and Sanger sequencing the following primers were used:

btuBF	aggaaaggtgcgatgattg
btuBFin	gttgttgggcgattatgc
btuBR	cgctcaacaataaacgcttc
galUF	tcggctggtggtactatc
galUR	tccctcgacgatttcctg
waaGF	atgagatgtatctttcggttattcc
waaGR	tgcccgccatttcaaatc
waaQF	cttgtggataagccatttcg

4.2 Transcriptomic analysis

The Fastq read outputs from the MiSeq instrument were mapped using Bowtie2 against the E. coli O157:H7 Sakai genome. As for the genome analysis, the chromosomal and pO157 and pOSAK1 plasmid sequences were introduced in a single fasta file, which was used as the reference for Bowtie2. Alignment statistics are shown below. Only mapped reads were then used to create bam files and read counts per gene were calculated using BEDtools coverageBed.[21] Differential expression analysis was performed using DESeq2.[22] For increased accuracy we used multi-factor design to account for sample pairing and hence differences between the samples, whilst measuring the effect of the treatment. The design formula used was thus dds <- DESeqDataSet (se, design = ~replicate + condition), whereby condition refers to treatment). Results show differentially expressed genes with a false discovery rate (FDR) cutoff of 0.05 and a P-adjusted value less than 0.05. No fold-change cut off was employed as we argue this would introduce an artificial assumption bias based on the importance of relative mRNA levels to phenotype. To identify overrepresented biological pathways in the dataset, the KEGG mapper Search&Color Pathway tool [23] was used and STRING [24] was used to visualize connections between differentially expressed genes. Raw reads and normalised count data can be found at the NCBI GEO database under entry GSE125633.

Data S1. (separate file)

DESeq2 analysis of the transcriptomic response of *E. coli* Sakai to **Λ-5b**. The worksheet named "all genes" contains the result of DESeq2 analysis on the expression of all the genes in the *E. coli* Sakai genome (Assembly: GCF_000008865.1; including plasmids pO157 and pOSAK1) in the presence of the compound compared to bacteria treated with solvent only (control). The genes with significantly higher transcript levels ($p_{adj} < 0.05$) when treated with the compound are listed in the worksheet named "up-regulated", while genes with significantly lower transcript levels in the presence of the compound are listed in the worksheet termed "down-regulated".

5. References

1. Howson, S. E.; Allan, L. E. N.; Chmel, N. P.; Clarkson, G. J.; Deeth, R. J.; Faulkner, A. D.; Simpson, D. H.; Scott, P., Origins of stereoselectivity in optically pure phenylethaniminopyridine tris-chelates M(NN ')(3)(n+) (M = Mn, Fe, Co, Ni and Zn). *Dalton Trans.* **2011,** *40* (40), 10416-10433.

2. Yao, S.; Ahn, H. Y.; Wang, X. H.; Fu, J.; Van Stryland, E. W.; Hagan, D. J.; Belfield, K. D., Donor-Acceptor-Donor Fluorene Derivatives for Two-Photon Fluorescence Lysosomal Imaging. *J. Org. Chem.* **2010,** *75* (12), 3965-3974.

3. Mitchell, R. H.; Iyer, V. S., An Improved Procedure for Bromomethylation of Aromatics Using Phase-Transfer Catalysis. Rapid Bis-haloalkylation. *Synlett* **1989,** *1989* (01), 55-57.

4. Fox, D. J.; Parris, S.; Pedersen, D. S.; Tyzack, C. R.; Warren, S., Asymmetric synthesis of cyclopropanes and dihydrofurans based on phosphine oxide chemistry. *Org. Biomol. Chem.* **2006,** *4* (16), 3108-3112.

5. O. V. Dolomanov, L. J. B., R. J. Gildea, J. A. K. Howard, H. Puschmann, OLEX2 : a complete structure solution, refinement and analysis program. *J. App. Cryst.* **2009,** *42* (2), 339--341.

6. Sheldrick, G. M., A short history of SHELX. *Acta Crystallogr., Sect. A* **2008,** *64* (1), 112-122.

7. Sheldrick, G. M., Crystal structure refinement with \it SHELXL. *Acta Cryst. C* **2015,** *71* (1), 3--8.

8. Coles, S. J.; Gale, P. A., Changing and challenging times for service crystallography. *Chem. Sci.* **2012,** *3* (3), 683-689.

9. Sheldrick, G. M., SHELXT - Integrated space-group and crystal-structure determination. *Acta Cryst. A* **2015,** *71*, 3--8.

10. Deeth, R. J.; Anastasi, A.; Diedrich, C.; Randell, K., Molecular modelling for transition metal complexes: Dealing with d-electron effects. *Coord. Chem. Rev.* **2009,** *253* (5-6), 795-816.

11. Deeth, R. J.; Fey, N.; Williams–Hubbard, B., DommiMOE: An implementation of ligand field molecular mechanics in the molecular operating environment. *J. Comput. Chem.* **2005,** *26* (2), 123-130.

12. Granovsky, A. A. Firefly version 8.

13. Schmidt, M. W.; Baldridge, K. K.; Boatz, J. A.; Elbert, S. T.; Gordon, M. S.; Jensen, J. H.; Koseki, S.; Matsunaga, N.; Nguyen, K. A.; Su, S.; Windus, T. L.; Dupuis, M.; Montgomery, J. A., General atomic and molecular electronic structure system. *J. Comp. Chem.* **1993,** *14* (11), 1347-1363.

14. Grimme, S.; Ehrlich, S.; Goerigk, L., Effect of the damping function in dispersion corrected density functional theory. *J. Comp. Chem.* **2011,** *32* (7), 1456-1465.

15. Alikhan, N.-F.; Zhou, Z.; Sergeant, M. J.; Achtman, M., A genomic overview of the population structure of Salmonella. *PLOS Genetics* **2018,** *14* (4), e1007261.

16. Langmead, B.; Salzberg, S. L., Fast gapped-read alignment with Bowtie 2. *Nature Methods* **2012,** *9*, 357.

17. Genome Project Data Processing, S.; Wysoker, A.; Handsaker, B.; Marth, G.; Abecasis, G.; Li, H.; Ruan, J.; Homer, N.; Durbin, R.; Fennell, T., The Sequence Alignment/Map format and SAMtools. *Bioinformatics* **2009,** *25* (16), 2078-2079.

18. Barrell, B.; Crook, J.; Parkhill, J.; Rutherford, K.; Rajandream, M.-A.; Rice, P.; Horsnell, T., Artemis: sequence visualization and annotation. *Bioinformatics* **2000,** *16* (10), 944-945.

19. Conesa, A.; García-Alcalde, F.; Dopazo, J.; Carbonell, J.; Okonechnikov, K.; Cruz, L. M.; Tarazona, S.; Götz, S.; Meyer, T. F., Qualimap: evaluating next-generation sequencing alignment data. *Bioinformatics* **2012,** *28* (20), 2678-2679.

20. Koboldt, D. C.; Zhang, Q.; Larson, D. E.; Shen, D.; McLellan, M. D.; Lin, L.; Miller, C. A.; Mardis, E. R.; Ding, L.; Wilson, R. K., VarScan 2: Somatic mutation and copy number alteration discovery in cancer by exome sequencing. *Genome Res.* **2012,** *22* (3), 568-576.

21. Quinlan, A. R.; Hall, I. M., BEDTools: a flexible suite of utilities for comparing genomic features. *Bioinformatics* **2010,** *26* (6), 841-842.

22. Love, M. I.; Huber, W.; Anders, S., Moderated estimation of fold change and dispersion for RNA-seq data with DESeq2. *Genome Biol.* **2014,** *15* (12), 550.

23. Tanabe, M.; Furumichi, M.; Goto, S.; Sato, Y.; Kanehisa, M., KEGG for integration and interpretation of large-scale molecular data sets. *Nucleic Acids Res.* **2011,** *40* (D1), D109-D114.

24. von Mering, C.; Jensen, L. J.; Snel, B.; Hooper, S. D.; Krupp, M.; Foglierini, M.; Jouffre, N.; Huynen, M. A.; Bork, P., STRING: known and predicted protein–protein associations, integrated and transferred across organisms. *Nucleic Acids Res.* **2005,** *33* (suppl_1), D433-D437.
